# Free Amino Group Transfer via α‐Amination of Native Carbonyls

**DOI:** 10.1002/anie.202304990

**Published:** 2023-06-05

**Authors:** Minghao Feng, Anthony J. Fernandes, Ana Sirvent, Eleonora Spinozzi, Saad Shaaban, Nuno Maulide

**Affiliations:** ^1^ Institute of Organic Chemistry University of Vienna Währinger Straße 38 1090 Vienna Austria; ^2^ Christian-Doppler Laboratory for Entropy-Oriented Drug Design Josef-Holaubek-Platz 2 1090 Vienna Austria

**Keywords:** α-Amino Carbonyls, Peptides, Primary Amine, Sulfonium [2,3]-Rearrangement

## Abstract

We report herein a straightforward transfer of a free amino group (NH_2_) from a commercially available nitrogen source to unfunctionalized, native carbonyls (amides and ketones) resulting in direct α‐amination. Primary α‐amino carbonyls are readily produced under mild conditions, further enabling diverse in situ functionalization reactions—including peptide coupling and Pictet–Spengler cyclization—that capitalize on the presence of the unprotected primary amine.

## Graphical abstract

An amino group transfer strategy was developed to install free NH_2_ to the α‐position of native carbonyls under mild conditions. The resulting primary α‐aminated products are amenable to in situ peptide coupling or Pictet–Spengler cyclization in one‐pot procedures. We anticipate this method to provide a general platform towards the synthesis of amines, ultimately opening exciting avenues in the synthesis and medicinal study of peptidomimetics.

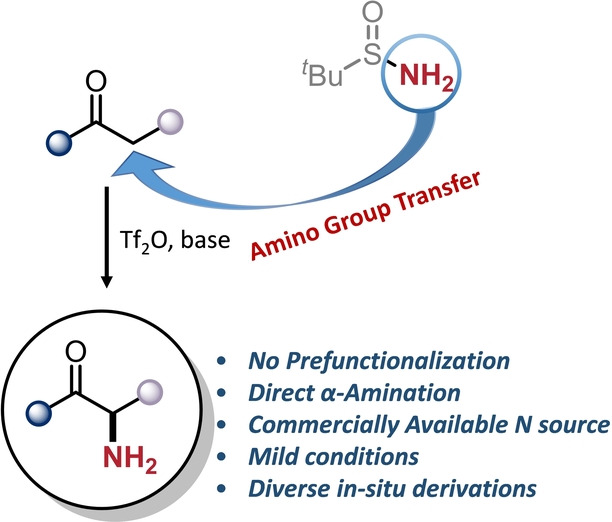

Nitrogen is the fourth most abundant element in small‐molecule drugs (Scheme 1A),[^1^, ^2^] rendering the introduction of an amino group to a small molecule at a predetermined position a long‐standing aim of synthetic chemists.^[3]^ Methods for the installation of a primary amino group (NH_2_) are particularly valuable, as they obviate the need to perform additional post‐synthetic transformations to liberate the desired functionality. Notably, only few such methods exist for the preparation of primary α‐amino carbonyls.^[4]^ Moreover, owing to the mismatched electronic nature of the α‐carbon of carbonyls and the nitrogen of amines, in many cases such an amination often requires prefunctionalization^[5]^ of the carbonyl partner in the form of α‐halogenation or similar (Scheme 1B). Conversely, and while amination with electrophilic aminating reagents is an important alternative strategy,^[6]^ most of these methods typically lead to α‐hydrazinyl or α‐aminoxy products requiring further modification to unveil the NH_2_ group. In 2019, Kürti and co‐workers reported a facile synthesis of primary α‐aminoketones from prefunctionalized carbonyls—in the form of silyl enol ethers—employing *O*‐(2,4‐dinitrophenyl) hydroxylamine or hydroxylamine‐*O*‐sulfonic acid (Scheme 1B).^[4d]^ Protocols for direct oxidative amination have been developed to circumvent this issue, in which the utilization of nucleophilic (often cyclic, secondary) amines as aminating reagents is crucial.^[7]^ The direct installation of the NH_2_ group onto native carbonyls therefore remains a challenging, unmet goal.

**Scheme 1:**
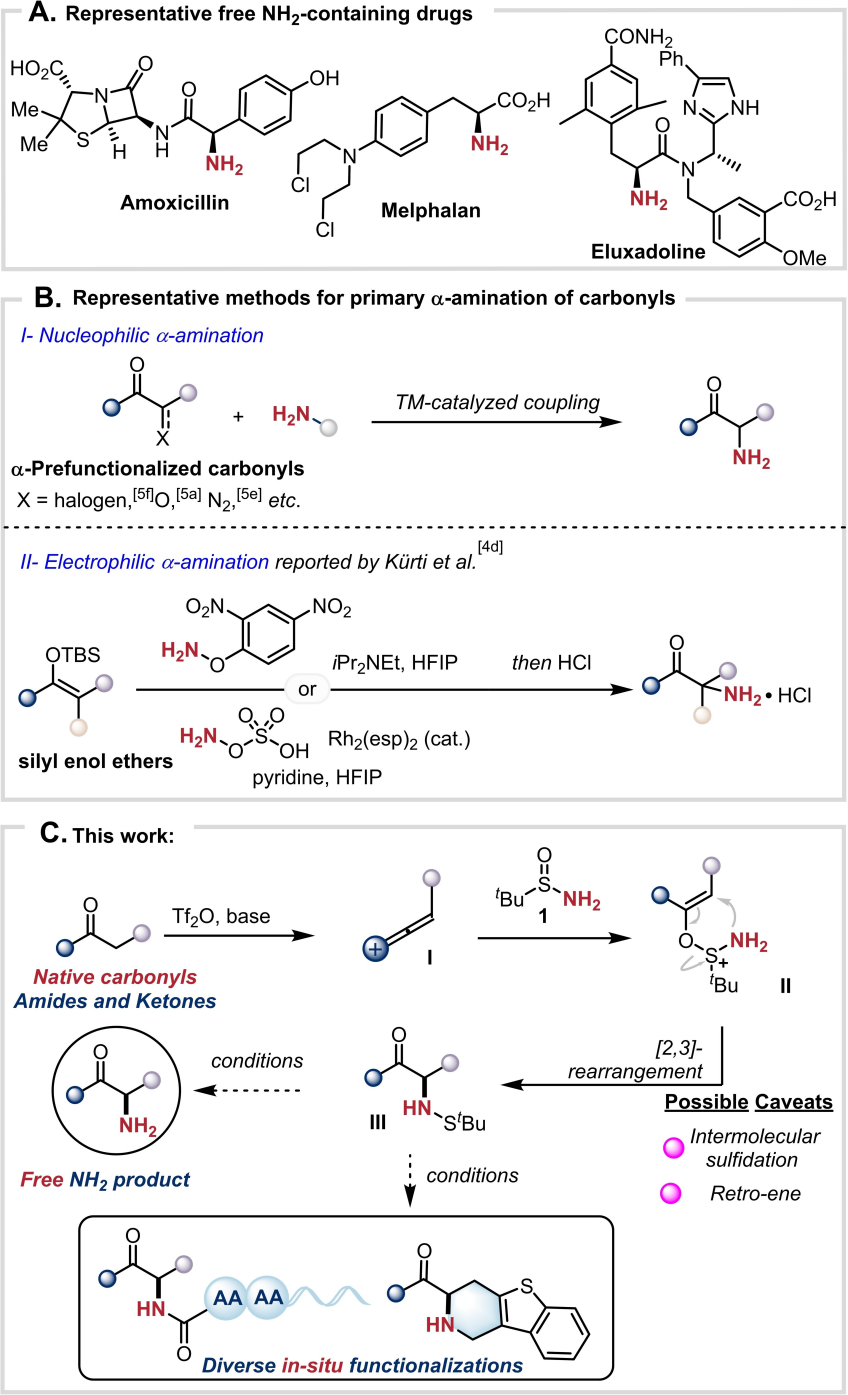
A) Selected examples of free NH_2_‐containing drugs. B) Representative methods for primary α‐amination of carbonyls. C) This work: Free α‐amination of unfunctionalized amides and ketones, and diverse in situ transformations.

Charge‐accelerated sulfonium rearrangement has emerged as a valuable strategy for the construction of C−C bonds and C−N bonds.^[8]^ Recently, our group reported a strategy enabling a direct coupling of carboxamides with sulfinimines for the synthesis of β‐amino amides via a sulfonium [3,3]‐sigmatropic rearrangement.^[8i]^ Inspired by that work, we envisioned that deploying *tert*‐butanesulfinamide^[9]^ **1** in conjunction with an appropriate vinyl cation (**I**)[^10^, ^11^] could enable C−N bond formation, installing the elusive NH_2_ group in a single direct step. However, this plan was not without its possible caveats: intermolecular sulfidation^[12a]^ or retro‐ene reaction^[12b]^ were potential side reactions (among others) of adduct **II**, leading to undesired α‐sulfanyl carbonyls or starting materials respectively. Furthermore, conditions to cleave the expected N−S bond of sulfenamide **III** in the same pot were also to be unveiled (Scheme 1C).

We first set out to attempt the α‐amination of amides employing *N*,*N*‐dimethyl‐4‐phenylbutanamide (**2 a**) as a model substrate. Gladly, under conditions previously found optimal for electrophilic amide activation (Tf_2_O, 2‐iodopyridine), the addition of *tert*‐butanesulfinamide **1** to the keteniminium ion of **2 a** resulted, after acidic work‐up (HCl in dioxane), in the formation of α‐NH_2_ amide **4 a** (68 % yield).^[8i]^ Despite 15 % of apparent starting material, likely stemming not from incomplete reaction, but rather from a competing retro‐ene process,^[12b]^ being recovered after the reaction, the anticipated side product resulting from α‐sulfidation was not observed.^[12a]^

We sought to demonstrate the broad applicability of this amination protocol. Hence, various amides were subjected to the standard reaction conditions and afforded the desired primary amine products in good yields (**4 b**–**h**, Scheme 2). Here, it is worth highlighting morpholine‐(**4 d**), Weinreb‐(**4 e**) and indolinyl‐amides (**4 f**), products amenable to transformation into other carbonyl derivatives. Moreover, the presence of a trifluoromethyl substituent near the reactive center was found not to negatively impact the reactivity (**4 h**). This protocol was found to also tolerate a large panel of functional groups, including alkenes (**4 i**, **4 j**), alkynes (**4 k**), allyl ethers (**4 l**), esters (**4 n**), nitriles (**4 o**) and phthalimides (**4 p**). Notably, an amide bearing a ketone moiety gave a comparatively low isolated yield (38 %) of the aminated product **4 m**; we believe this to result from undesired condensation side‐reactions taking place under the acidic conditions employed for cleavage of the N−S bond. Various heteroaromatic and aromatic motifs were also well‐tolerated (**4 q**–**t**). Cyclopropyl substrate **3 u** also smoothly converted to the desired product **4 u**, exempt from any ring opening or decomposition of the strained 3‐membered ring. Remarkably, 13‐membered ring lactam could also be efficiently aminated to afford **4 v**.

**Scheme 2:**
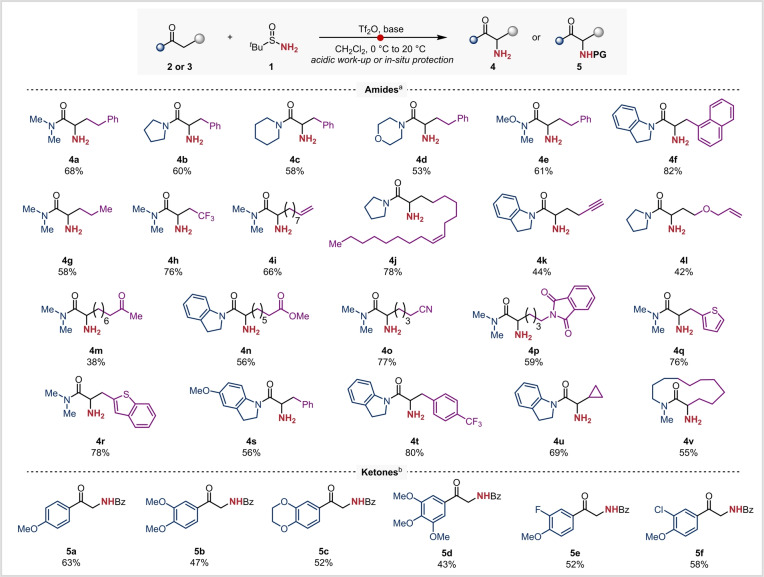
Conditions a) amination of amides: reactions were performed on a 0.2 mmol scale: amide (1.0 equiv.), 2‐iodopyridine (2.2 equiv.), Tf_2_O (1.2 equiv.), *tert‐*butanesulfinamide (2.0 equiv.), CH_2_Cl_2_ (0.1 M), 0 °C to 20 °C, 24 h, then acidic workup (HCl in dioxane, 20 °C, 16 h). Conditions b) amination of ketones: reactions were performed on a 0.2 mmol scale: ketone (1.0 equiv.), DTBMP (1.2 equiv.), Tf_2_O (1.2 equiv.), *tert*‐butanesulfinamide (2.0 equiv.), CH_2_Cl_2_ (0.1 M), 0 °C to 20 °C, 24 h then protective workup (BzCl, NEt_3_, CH_2_Cl_2_, 20 °C, 16 h). DTBMP: 2,6‐di‐*tert*‐butyl‐4‐methyl‐pyridine. See Supporting Information for details.

Electron‐rich aryl ketones are also candidates for electrophilic activation to form highly reactive intermediates,^[11]^ a prerequisite for this α‐amination reaction. In this case, the acidic work‐up led to side reactions such as condensation of the generated primary amine with the ketone group. To circumvent this issue, in situ benzoylation of the free NH_2_ group was conducted throughout, enabling the isolation of a wide array of *N*‐protected α‐aminated ketones (**5 a**–**f**).

The work presented herein portrays a rare transformation deploying a free NH_2_ group. Apart from its unique character, one of the benefits of such a process is the potential to directly capitalize on the rich chemistry of amines with in situ chemistry. For instance, free NH_2_ groups are ideally suited for peptide coupling and indeed, generated amines **4 d**, **4 v** and **4 q** could be successfully coupled in situ in a one‐pot fashion with different amino acids. It is noteworthy that this amounts to a formal and unprecedented “α‐peptidation” of previously unfunctionalized amides in a single step, to yield the corresponding peptides **6 a**–**d** in excellent yields (Scheme 3A). Interestingly, compound **6 d** was obtained in very good yield and a 9 : 1 mixture of diastereomers when enantiopure sulfonamide **(*R*)‐1** was employed.

**Scheme 3:**
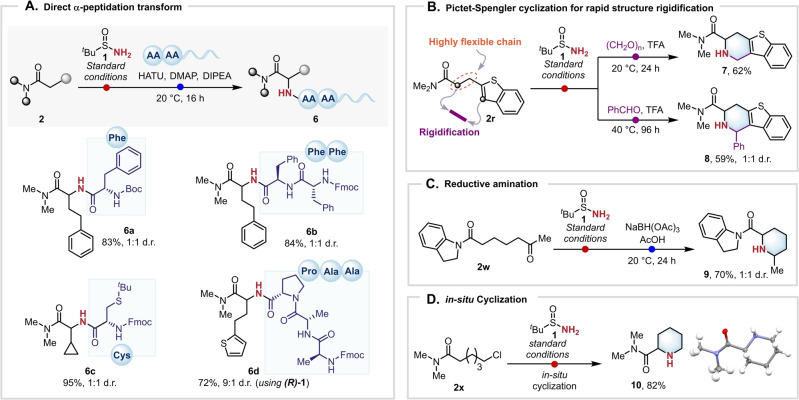
α‐Amination/functionalization of amides. Reactions were performed on a 0.2 mmol scale. A) Direct α‐peptidation transform: amide (1.0 equiv.), 2‐iodopyridine (2.2 equiv.), Tf_2_O (1.2 equiv.), *tert*‐butanesulfinamide (2.0 equiv.), CH_2_Cl_2_ (0.1 M), 0 °C to 20 °C, 24 h then amino acid (2.0 equiv), HATU (2.2 equiv.), DIPEA (2.2 equiv.), CH_2_Cl_2_ (0.04 M), 20 °C, 3 h. B) α‐Amination/Pictet–Spengler: amide (1.0 equiv.), 2‐iodopyridine (2.2 equiv.), Tf_2_O (1.2 equiv.), *tert*‐butanesulfinamide (2.0 equiv.), CH_2_Cl_2_ (0.1 M), 0 °C to 20 °C, 24 h then aldehyde (2.0 equiv.), TFA (4.0 equiv.), 20 °C, 24 h or 40 °C, 96 h. C) α‐Amination/reductive amination: amide (1.0 equiv.), 2‐iodopyridine (2.2 equiv.), Tf_2_O (1.2 equiv.), *tert*‐butanesulfinamide (2.0 equiv.), CH_2_Cl_2_ (0.1 M), 0 °C to 20 °C, 24 h then NaBH(OAc)_3_ (2.0 equiv.), AcOH (1.0 equiv.), 20 °C, 24 h. D) in situ Cyclization: amide (1.0 equiv.), 2‐iodopyridine (2.2 equiv.), Tf_2_O (1.2 equiv), *tert*‐butanesulfinamide (2.0 equiv.), CH_2_Cl_2_ (0.1 M), 0 °C to 20 °C, 24 h, then 4 M HCl in dioxane, 20 °C, 16 h. HATU: Hexafluorophosphate Azabenzotriazole Tetramethyl Uronium. DMAP: 4‐Dimethylaminopyridine. DIPEA: *N*,*N*‐Diisopropylethylamine. TFA: Trifluoroacetic acid.

In addition, primary amines such as **4 r** were found to be prime substrates for Pictet–Spengler cyclization to directly generate piperidines **7** and **8**,^[13]^ employing paraformaldehyde or benzaldehyde as the carbonyl partner, respectively (Scheme 3B). This transformation allows the direct “stitching” of the α carbon of the amide in **2 r** and the heteroarene CH, with the amino moiety serving as a linchpin—a potentially very useful transformation for drug discovery. Moreover, ketoamide **2 w** was directly converted to piperidine **9** through a one‐pot α‐amination/reductive amination sequence (Scheme 3C),^[14]^ whereas an amide bearing an ω‐chlorine atom on the side chain (**2 x**) could be successfully engaged in a tandem amination‐cyclization process, furnishing piperidine **10** in excellent yield (Scheme 3D).^[15]^

From a mechanistic point of view, we believe that nucleophilic attack of *tert*‐butanesulfinamide on the keteniminium **I** or its congener (**I′**) generates adduct **II** (Scheme 4). This sulfonium species **II** is then thought to undergo a charge‐accelerated [2,3]‐rearrangement[^8j^, ^16^] to yield sulfenamide **III**.^[17]^ Noteworthy, both sulfenamides of type **III** and primary α‐aminated products were detected by LC/MS analysis before the acidic work‐up, supporting this mechanistic pathway. The acidic work‐up accomplishes complete cleavage of the N−S bond.

**Scheme 4:**
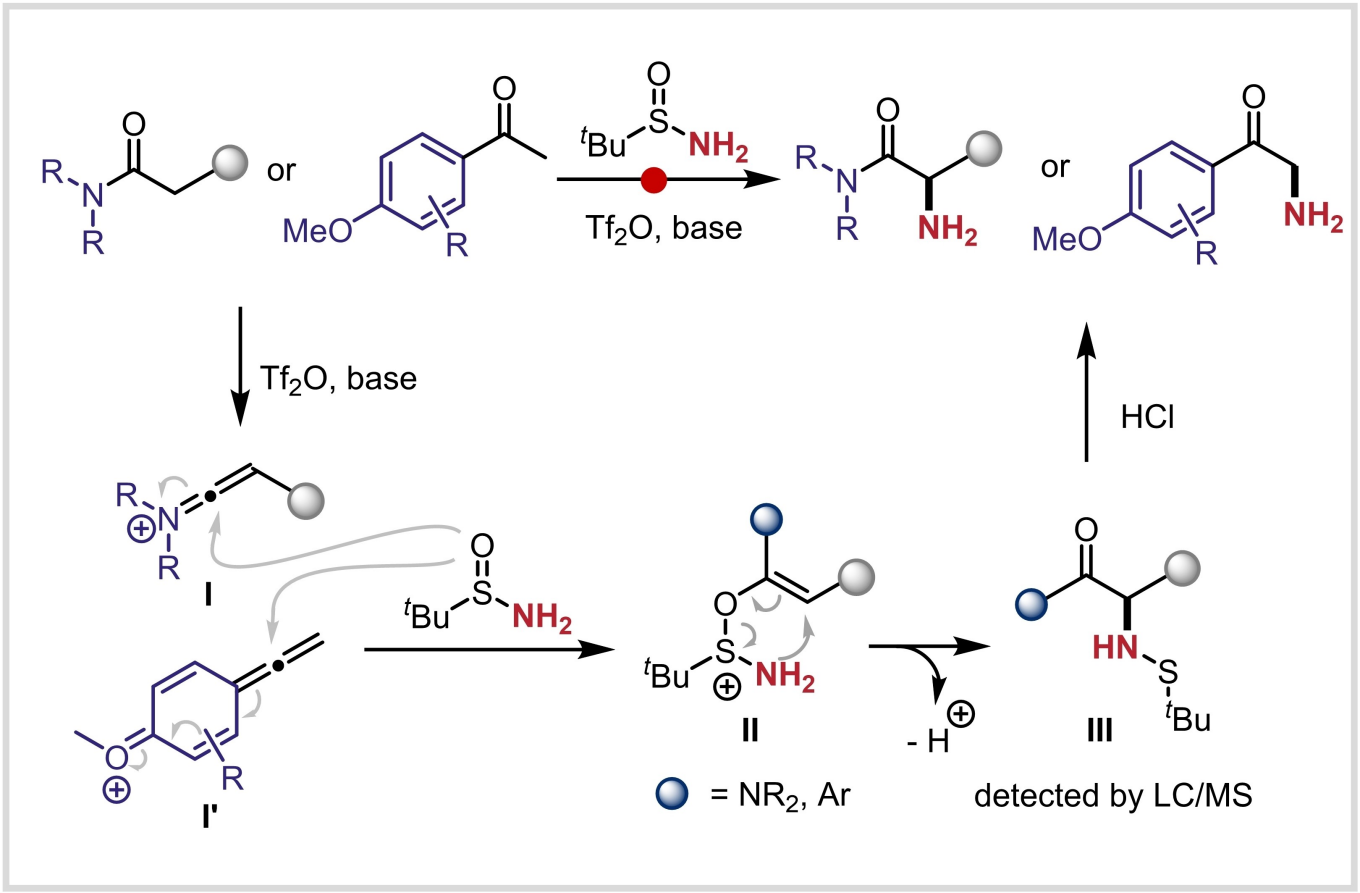
Plausible mechanism for the direct α‐amination of ketones and amides.

In conclusion, we have developed a facile method for the direct installation of primary amines (NH_2_) into native carbonyls under mild conditions. Moreover, this strategy allows for a wide range of possible in situ derivatization reactions, including peptide coupling and Pictet–Spengler cyclization. We anticipate this method to provide a general platform towards the synthesis of unnatural α‐amino acid derivatives, which we believe opens up exciting avenues for further research in the field of synthesis and medicinal study of peptidomimetics.

## Conflict of interest

The authors declare no conflict of interest.

## Supporting information

As a service to our authors and readers, this journal provides supporting information supplied by the authors. Such materials are peer reviewed and may be re‐organized for online delivery, but are not copy‐edited or typeset. Technical support issues arising from supporting information (other than missing files) should be addressed to the authors.

Supporting Information

## Data Availability

The data that support the findings of this study are available in the Supporting Information of this article.
