# Supplementary material for: Free Amino Group Transfer via α‐Amination of Native Carbonyls
Source: Angew Chem Int Ed Engl. 2023 Jun 5;62(28):e202304990. doi: 10.1002/anie.202304990 (PMC10952782; doi:10.1002/anie.202304990)
Supplement: Supplementary file 1 — Supporting Information [file ANIE-62-0-s001.pdf]

Angewandte Chemie
Eine Zeitschrift der Gesellschaft Deutscher Chemiker

Supporting Information

Free Amino Group Transfer via α-Amination of Native Carbonyls

*M. Feng, A. J. Fernandes, A. Sirvent, E. Spinozzi, S. Shaaban, N. Maulide**

Table of Contents

1. **General information** 2
2. **Substrate preparation** 3
3. **General procedure for the α-free amination of amides** 12
4. **General procedure for the α-amination of ketones** 23
5. **Free α-amination/functionalization of amides** 26

5.1. α-Amination/peptide coupling 26

5.2. α-Amination/Pictet-Spengler cyclizations 29

5.3. α-Amination/Reductive amination 32

5.4. α-Amination/direct cyclization 33

6. **NMR Spectra** 34
7. **X-ray data** 88
8. **References** 90

1. General information

Unless otherwise stated, all glassware was flame-dried before use and all reactions were performed under an atmosphere of argon. All solvents were distilled from appropriate drying agents prior to use or, if purchased in anhydrous form, used as received from commercial suppliers. Triflic anhydride was distilled over P_4O_{10} prior to use.[1] All other reagents were used as received from commercial suppliers, unless otherwise stated. Reaction progress was monitored by thin layer chromatography (TLC) performed on aluminium plates coated with silica gel F_{254} with 0.2 mm thickness. Chromatograms were visualized by fluorescence quenching with UV light at 254 nm or by staining using potassium permanganate. Flash column chromatography was performed using silica gel 60 (230-400 mesh, Merck and co.). Neat infra-red spectra were recorded using a Perkin-Elmer Spectrum 100 FT-IR spectrometer. Wavenumbers (ν_{max}) are reported in cm^{-1}. Mass spectra were obtained using a Finnigan MAT 8200 or (70 eV) or an Agilent 5973 (70 eV) spectrometer, using electrospray ionization (ESI). All ^{1}H NMR, ^{13}C NMR and ^{19}F NMR spectra were recorded using a Bruker AV-400, AV-600 or AV-700 spectrometer at 300K. Chemical shifts are given in parts per million (ppm, δ), referenced to the solvent peak of $CDCl_3$ [defined at δ = 7.26 ppm (^{1}H NMR) and δ = 77.16 (^{13}C NMR)], CD_3OD [defined at δ = 3.31 ppm (^{1}H NMR) and δ = 49.00 (^{13}C NMR)], or DMSO-d_6 [defined at δ = 2.50 ppm (^{1}H NMR) and δ = 32.52 (^{13}C NMR)]. Coupling constants are quoted in Hz (*J*). ^{1}H NMR splitting patterns were designated as singlet (s), doublet (d), triplet (t), quartet (q), and pentet (p). Splitting patterns that could not be interpreted or easily visualized were designated as multiplet (m) or broad (br). Selected ^{13}C NMR spectra were recorded using the attached proton test (APT) to facilitate the confirmation and assignment of the structure. Optical rotations were measured on a Unipol L 2000 polarimeter using a 100 mm path-length cell at 589 nm (c given in g/100 mL).

2. Substrate preparation

General Procedure A:

To a solution of the amine (1.00 equiv.) and triethylamine (2.00 equiv.) in CH_2Cl_2 (0.1 M) at 0 °C, the corresponding acyl chloride (1.20 equiv.) was added dropwise and the resulting reaction mixture was allowed to warm to 25 °C while stirring overnight (14 h). After this time, a saturated aqueous solution of sodium bicarbonate was added and the biphasic system was separated. The aqueous phase was extracted with CH_2Cl_2 (three times) and the organic phases were combined and dried over anhydrous sodium sulfate. The dried solution was filtered and concentrated under reduced pressure. The resulting crude material was purified by flash column chromatography on silica gel (heptane/ethyl acetate) to afford the desired compound.

General Procedure B:

To a solution of the carboxylic acid (1.00 equiv.), triethylamine (1.00 equiv.), 4-dimethylaminopyridine (DMAP, 10 mol%) and 1-ethyl-3-(3-dimethylaminopropyl)carbodiimide hydrochloride (EDCI·HCl, 1.50 equiv.) in CH_2Cl_2 (0.10 M), the corresponding amine (1.20 equiv.) was added and the resulting solution was stirred at 25 °C overnight (14 h). After this time, the organic solution was washed sequentially with 0.5 M aqueous hydrochloric acid, saturated aqueous sodium bicarbonate and saturated aqueous sodium chloride. The washed solution was dried over anhydrous sodium sulfate, filtered and concentrated under reduced pressure. The resulting crude material was purified by flash column chromatography on silica gel (heptane/ethyl acetate) to afford the desired compound.

Characterizations of the prepared amides

***N,N*-Dimethyl-4-phenylbutanamide (2a)**

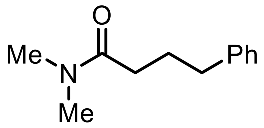

The title compound was obtained in 92% yield using General Procedure A. All analytical data were in good accordance with data reported in the literature.[2]

3-Phenyl-1-(pyrrolidin-1-yl)propan-1-one (2b)

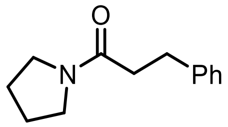

The title compound was obtained in 96% yield using General Procedure A. All analytical data were in good accordance with data reported in the literature.[3]

3-Phenyl-1-(piperidin-1-yl)propan-1-one (2c)

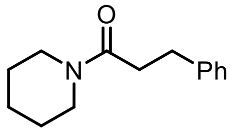

The title compound was obtained in 91% yield using General Procedure A. All analytical data were in good accordance with data reported in the literature.[4]

1-Morpholino-4-phenylbutan-1-one (2d)

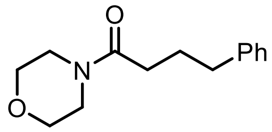

The title compound was obtained in 88% yield using General Procedure B. All analytical data were in good accordance with data reported in the literature.[5]

***N*-Methoxy-*N*-methyl-4-phenylbutanamide (2e)**

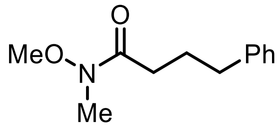

The title compound was obtained in 71% yield using General Procedure B. All analytical data were in good accordance with data reported in the literature.[6]

1-(Indolin-1-yl)-3-(naphthalen-1-yl)propan-1-one (2f)

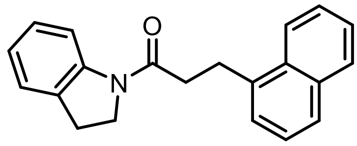

The title compound was obtained in 85% yield using General Procedure B.

^{1}H NMR (600 MHz, CDCl$_3$) δ 8.31 (d, *J* = 8.1 Hz, 1H), 8.09 (d, *J* = 8.4 Hz, 1H), 7.88 (d, *J* = 8.0 Hz, 1H), 7.79 – 7.72 (m, 1H), 7.51 (ddd, *J* = 15.7, 10.9, 3.9 Hz, 2H), 7.41 (dd, *J* = 7.6, 5.3 Hz, 2H), 7.23 (t, *J* = 7.7 Hz, 1H), 7.16 (d, *J* = 7.3 Hz, 1H), 7.02 (t, *J* = 7.4 Hz, 1H), 3.90 (t, *J* = 8.5 Hz, 2H), 3.60 – 3.52 (m, 2H), 3.11 (t, *J* = 8.4 Hz, 2H), 2.89 – 2.82 (m, 2H).

^{13}C NMR (150 MHz, CDCl$_3$) δ 170.4, 142.9, 137.3, 133.9, 131.6, 131.0, 128.9, 127.6, 127.0, 126.2, 126.1, 125.7, 125.6, 124.5, 123.6, 123.5, 117.0, 47.9, 37.1, 28.0, 27.8.

IR (neat) ν: 1656, 1597, 1481, 1410, 1337, 1263, 802, 779, 755 cm^{-1}.

HRMS (ESI$^+$): exact mass calculated for $[M+H]^+$ ($C_{21}H_{20}NO^+$) requires m/z 302.1539, found m/z 302.1543.

***N,N*-Dimethylpentanamide (2g)**

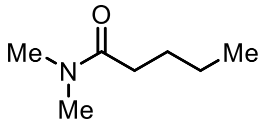

The title compound was obtained in 98% yield using General Procedure A. All analytical data were in good accordance with data reported in the literature.[3]

4,4,4-Trifluoro-*N,N*-dimethylbutanamide (2h)

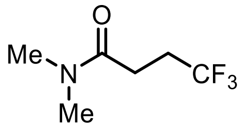

The title compound was obtained in 75% yield using General Procedure B. All analytical data were in good accordance with data reported in the literature.[7]

***N,N*-Dimethylundec-10-enamide (2i)**

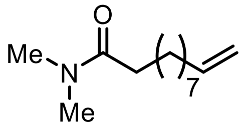

The title compound was obtained in 79% yield using General Procedure B. All analytical data were in good accordance with data reported in the literature.[8]

(*Z*)-1-(Pyrrolidin-1-yl)octadec-9-en-1-one (2j)

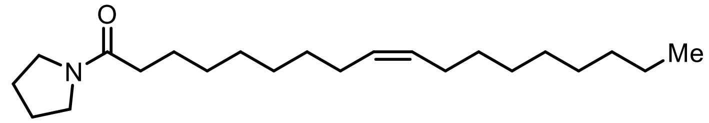

The title compound was obtained in 88% yield using General Procedure B.

^{1}H NMR (400 MHz, CDCl$_3$) δ 5.41 – 5.26 (m, 2H), 3.43 (dt, *J* = 22.0, 6.8 Hz, 4H), 2.31 – 2.19 (m, 2H), 2.07 – 1.97 (m, 4H), 1.93 (dd, *J* = 13.2, 6.8 Hz, 2H), 1.89 – 1.80 (m, 2H), 1.63 (dd, *J* = 14.8, 7.3 Hz, 2H), 1.31 – 1.26 (m, 20H), 0.88 (t, *J* = 6.9 Hz, 3H).

^{13}C NMR (100 MHz, CDCl$_3$) δ 171.8, 129.9, 129.8, 46.6, 45.6, 34.9, 31.9, 29.8, 29.7, 29.5 (2C), 29.4, 29.3 (2C), 29.2, 27.2(2C), 26.1, 24.9, 24.4, 22.7, 14.1.

IR (neat) ν: 2923, 2852, 1646, 1427, 1343, 1225, 1194, 756, 730 cm^{-1}.

HRMS (ESI$^+$): exact mass calculated for $[M+H]^+$ ($C_{22}H_{42}NO^+$) requires m/z 336.3261, found m/z 336.3261.

1-(Indolin-1-yl)hex-5-yn-1-one (2k)

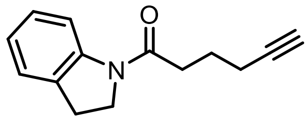

The title compound was obtained in 63% yield using General Procedure B.

^{1}H NMR (400 MHz, CDCl$_3$) δ 8.23 (d, *J* = 8.0 Hz, 1H), 7.19 (t, *J* = 7.9 Hz, 2H), 7.01 (dd, *J* = 8.2, 7.6 Hz, 1H), 4.08 (t, *J* = 8.5 Hz, 2H), 3.20 (t, *J* = 8.5 Hz, 2H), 2.58 (t, *J* = 7.2 Hz, 2H), 2.36 (td, *J* = 6.7, 2.6 Hz, 2H), 2.03 – 1.92 (m, 3H).

^{13}C NMR (101 MHz, CDCl$_3$) δ 170.5, 143.0, 131.0, 127.5, 124.5, 123.5, 116.9, 83.8, 69.0, 47.9, 34.1, 28.0, 23.1, 17.8.

IR (neat) ν: 3268, 3216, 2331, 1651, 1596, 1482, 1408, 1307, 1261, 1162, 766, 735, 719, 656 cm^{-1}.

HRMS (ESI$^+$): exact mass calculated for $[M+H]^+$ ($C_{14}H_{16}NO^+$) requires m/z 214.1226, found m/z 214.1225.

4-(Allyloxy)-1-(pyrrolidine-1-yl)butan-1-one (2l)

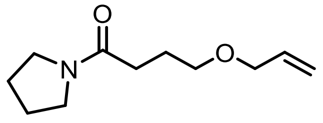

The title compound was obtained in 87% yield using a procedure described in the literature.[9] All analytical data were in good accordance with data reported in the literature.[9]

***N,N*-Dimethyl-10-oxoundecanamide (2m)**

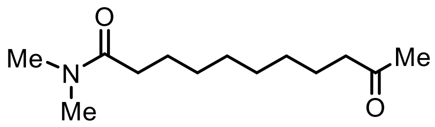

To a solution of the *N,N*-dimethylundec-10-enamide (**2i**, 1.00 equiv.) and Pd(OAc)$_2$ (0.10 equiv.) in DMSO/water (0.33 M, 10/1, v/v) under oxygen atmosphere was added TFA (1.00 equiv.). The mixture was heated to 70 °C and stirred for 16 h. After letting it cool to 25 °C, it was diluted with water and then extracted with ethyl acetate. The organic layer was washed with brine, dried over Na_2SO_4 and concentrated under reduced pressure. The crude product was purified through flash column chromatography on silica gel (ethyl acetate/heptane) to afford the title compound in 56% yield.

^{1}H NMR (400 MHz, CDCl$_3$) δ 2.99 (s, 3H), 2.93 (s, 3H), 2.40 (t, *J* = 7.4 Hz, 2H), 2.31 – 2.25 (m, 2H), 2.12 (s, 3H), 1.65 – 1.50 (m, 4H), 1.35 – 1.23 (m, 8H).

^{13}C NMR (101 MHz, CDCl$_3$) δ 209.5, 173.3, 43.9, 37.4, 35.5, 33.5, 30.0, 29.6, 29.3 (2C), 29.2, 25.2, 23.9.

IR (neat) ν: 2928, 2853, 1714, 1648, 1501, 1494, 1465, 1412, 1396, 1356, 1161 cm^{-1}.

HRMS (ESI$^+$): exact mass calculated for $[M+Na]^+$ ($C_{13}H_{25}NNaO_2^+$) requires m/z 250.1778, found m/z 250.1778.

Methyl 9-(indolin-1-yl)-9-oxononanoate (2n)

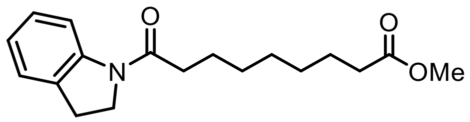

The title compound was obtained in 76% yield using General Procedure B. All analytical data were in good accordance with data reported in the literature.[10]

6-Cyano-*N*,*N*-dimethylhexanamide (2o)

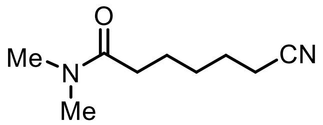

To a solution of the 6-chloro-*N*,*N*-dimethylhexanamide (**2w**, 1.00 eq.), the corresponding salt (2.50 eq.) was added in DMF (0.40 M), and the resulting solution was stirred at 60 °C for 24 h. After this time, the organic solution was extracted with 0.50 M aqueous hydrochloric acid and washed three times with brine. Afterwards, the organic phase was concentrated under reduced pressure. The resulting crude material was purified by flash column chromatography on silica gel (heptane/ethyl acetate) to afford the title compound in 84% yield.

^{1}H NMR (400 MHz, CDCl$_3$) δ 3.00 (s, 3H), 2.94 (s, 3H), 2.34 (dt, *J* = 12.6, 7.2 Hz, 4H), 1.69 (dq, *J* = 15.4, 7.6 Hz, 4H), 1.55 – 1.43 (m, 2H).

^{13}C NMR (151 MHz, CDCl$_3$) δ 172.4, 119.7, 37.2, 35.4, 32.8, 28.4, 25.3, 24.1, 17.0.

IR (neat) ν: 2934, 2855, 1740, 1647, 1641, 1463, 1398, 1141, 474, 455, 437, 418 cm^{-1}.

HRMS (ESI$^+$): exact mass calculated for $[M+Na]^+$ ($C_9H_{16}N_2ONa^+$) requires m/z 191.1155, found m/z 191.1145.

6-(1,3-Dioxoisoindolin-2-yl)-*N,N*-dimethylhexanamide (2p)

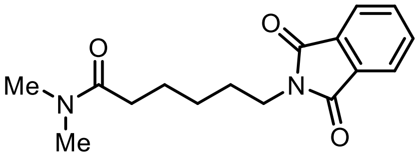

To a solution of the 6-chloro-*N*,*N*-dimethylhexanamide (**2w**, 1.00 equiv.), the corresponding salt (2.5 equiv.) was added in DMF (0.40 M), and the resulting solution was stirred at 60 °C for 24 h. After this time, the organic solution was extracted with 0.50 M aqueous hydrochloric acid and washed three times with brine. Afterwards, the organic phase was concentrated under reduced pressure. The resulting crude material was purified by flash column chromatography on silica gel (heptane/ethyl acetate) to afford the title compound in 48% yield.

^{1}H NMR (400 MHz, $CDCl_3$) δ 7.83 (dt, *J* = 7.5, 3.8 Hz, 2H), 7.74 – 7.66 (m, 2H), 3.69 (t, *J* = 7.2 Hz, 2H), 2.99 (s, 3H), 2.92 (s, 3H), 2.34 – 2.26 (m, 2H), 1.74 – 1.66 (m, 2H), 1.62 (d, *J* = 8.5 Hz, 2H), 1.45 – 1.34 (m, 2H).

^{13}C NMR (151 MHz, $CDCl_3$) δ 172.8 (2C), 168.4, 133.8 (2C), 132.1 (2C), 123.1 (2C), 37.9, 37.2, 35.3, 33.1, 28.4, 26.7, 24.6.

IR (neat) ν: 2940, 1771, 1708, 1647, 1501, 1466, 1437, 1396, 1389, 1045, 721, 530 cm^{-1}.

HRMS (ESI$^+$): exact mass calculated for $[M+Na]^+$ ($C_{16}H_{20}N_2O_3Na^+$) requires m/z 311.1366, found m/z 311.1374.

***N,N*-Dimethyl-3-(thiophen-2-yl)propenamide (2q)**

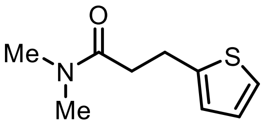

The title compound was obtained in 63% yield using General Procedure B. All analytical data were in good accordance with data reported in the literature.[11]

3-(Benzo[*b*]thiophen-2-yl)-*N,N*-dimethylpropanamide (2r)

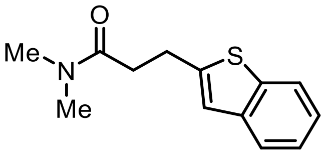

The title compound was obtained in 82% yield using General Procedure B.

^{1}H NMR (400 MHz, $CDCl_3$) δ 7.75 (d, *J* = 7.9 Hz, 1H), 7.66 (d, *J* = 7.4 Hz, 1H), 7.32 – 7.22 (m, 2H), 7.06 (d, *J* = 0.6 Hz, 1H), 3.32 – 3.21 (m, 2H), 2.99 (s, 3H), 2.97 (s, 3H), 2.77 – 2.69 (m, 2H).

^{13}C NMR (101 MHz, CDCl$_3$) δ 171.3, 145.2, 140.1, 139.4, 124.1, 123.6, 122.8, 122.1, 121.1, 37.1, 35.5, 34.7, 26.3.

IR (neat) ν: 1639, 1492, 1435, 1410, 1396, 1140, 826, 745, 726 cm^{-1}.

HRMS (ESI$^+$): exact mass calculated for [M+H]$^+$ ($C_{13}H_{16}NSO^+$) requires m/z 234.0947, found m/z 234.0948.

1-(5-Methoxyindolin-1-yl)-3-phenylpropan-1-one (2s)

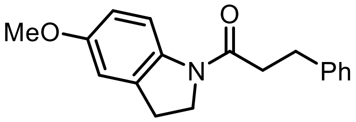

The title compound was obtained in 80% yield using General Procedure A.

^{1}H NMR (400 MHz, CDCl$_3$) δ 8.18 (d, J = 9.6 Hz, 1H), 7.34 – 7.17 (m, 5H), 6.73 (d, J = 7.8 Hz, 2H), 3.95 (t, J = 8.4 Hz, 2H), 3.78 (s, 3H), 3.17 – 3.01 (m, 4H), 2.82 – 2.62 (m, 2H).

^{13}C NMR (151 MHz, CDCl$_3$) δ 169.8, 156.3, 141.4, 136.9, 132.8, 128.7 (2C), 128.6 (2C), 126.3, 117.7, 112.0, 111.0, 55.8, 48.2, 37.8, 31.0, 28.3.

IR (neat) ν: 2955, 2937, 2833, 1638, 1485, 1407, 1258, 1031, 701 cm^{-1}.

HRMS (ESI$^+$): exact mass calculated for [M+H]$^+$ ($C_{18}H_{20}NO_2^+$) requires m/z 282.1489, found m/z 282.1489.

1-(Indolin-1-yl)-3-(4-(trifluoromethyl)phenyl)propan-1-one (2t)

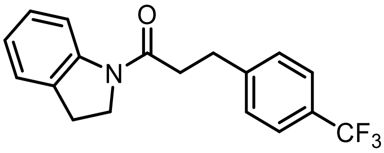

The title compound was obtained in 88% yield using General Procedure B.

^{1}H NMR (600 MHz, CDCl$_3$) δ 8.25 (d, J = 8.1 Hz, 1H), 7.55 (d, J = 8.0 Hz, 2H), 7.38 (d, J = 8.0 Hz, 2H), 7.23 – 7.16 (m, 2H), 7.02 (t, J = 7.4 Hz, 1H), 3.98 (t, J = 8.5 Hz, 2H), 3.19 – 3.12 (m, 4H), 2.74 (t, J = 7.6 Hz, 2H).

^{13}C NMR (150 MHz, CDCl$_3$) δ 169.7, 145.4, 142.8, 131.0, 128.8, 128.5 (q, $^2J_{C\text{-}F}$ = 18.9 Hz, 2C), 127.6, 125.4 (q, $^3J_{C\text{-}F}$ = 4.0 Hz, 2C), 124.9 (q, $^1J_{C\text{-}F}$ = 270 Hz), 124.5, 123.7, 117.0, 47.9, 37.2, 30.3, 28.0.

^{19}F NMR (565 MHz, CDCl$_3$) δ -62.35.

IR (neat) ν: 1657, 1599, 1482, 1461, 1411, 1325, 1288, 1261, 1162, 1119, 1067, 1019, 828, 756 cm^{-1}.

HRMS (ESI$^+$): exact mass calculated for $[M+H]^+$ ($C_{18}H_{17}F_3NO^+$) requires m/z 320.1257, found m/z 320.1258.

2-Cyclopropyl-1-(indolin-1-yl)ethan-1-one (2u)

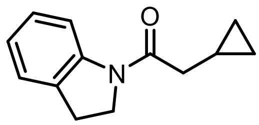

The title compound was obtained in 99% yield using General Procedure B. All analytical data were in good accordance with data reported in the literature.[12]

1-Methylazacyclotridecan-2-one (2v)

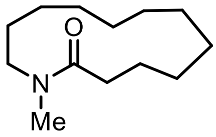

Sodium hydride (1.10 equiv., 60% suspension in mineral oil) was added to a solution of lactam (1.00 equiv.) in DMF (0.20 M) at 0 °C and stirred for 1 h. Subsequently, methyl iodide (3.00 equiv.) was slowly added to the mixture and the resulting suspension was stirred for 12 h. The mixture was poured into 50 mL of water, the aqueous layer was extracted with CH_2Cl_2 (three times), and the organic phases were combined and dried over anhydrous sodium sulfate. The dried solution was filtered and concentrated under reduced pressure. The resulting crude material was purified by flash column chromatography on silica gel 0 to 20% DMA/CH_2Cl_2, DMA solution is prepared from CH_2Cl_2/methanol/ammonia = 90:9:1) to afford the desired compound as an off-white solid (62%). All analytical data were in good accordance with data reported in the literature.[10]

1-(Indolin-1-yl)heptane-1,6-dione (2w)

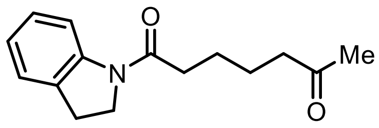

The title compound was obtained in 89% yield using General Procedure B.

^{1}H NMR (600 MHz, CDCl$_3$) δ 8.22 (d, J = 8.0 Hz, 1H), 7.20 – 7.15 (m, 2H), 7.00 (t, J = 7.4 Hz, 1H), 4.04 (t, J = 8.5 Hz, 2H), 3.19 (t, J = 8.4 Hz, 2H), 2.50 (t, J = 7.1 Hz, 2H), 2.43 (t, J = 7.0 Hz, 2H), 2.15 (s, 3H), 1.81 – 1.65 (m, 4H).

^{13}C NMR (151 MHz, CDCl$_3$) δ 208.9, 171.0, 143.2, 131.1, 127.7, 124.6, 123.7, 117.1, 48.1, 43.7, 35.8, 30.1, 28.2, 24.1, 23.6.

IR (neat) ν: 1712, 1648, 1476, 1408, 765 cm^{-1}.

HRMS (ESI⁺): exact mass calculated for $[M+Na]^+$ ($C_{15}H_{19}NO_2Na^+$) requires m/z 268.1308, found m/z 268.1308.

6-Chloro-*N,N*-dimethylhexanamide (2x)

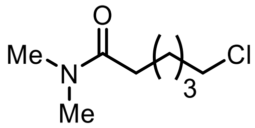

The title compound was obtained in 99% yield using General Procedure B. All analytical data were in good accordance with data reported in the literature.[13]

2-Cyclopropyl-*N,N*-dimethylacetamide (2y)

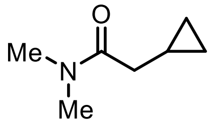

The title compound was obtained in 50% yield using General Procedure B. All analytical data were in good accordance with data reported in the literature.[14]

3. General procedure for the α-free amination of amides

General Procedure C:

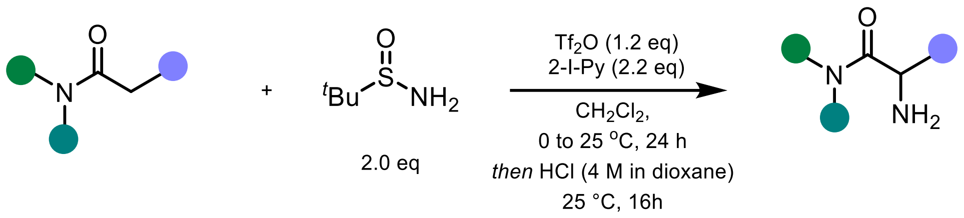

To a mixture of amide (0.200 mmol, 1.00 equiv.), 2-iodopyridine (46.8 µL, 0.440 mmol, 2.20 equiv.) in DCM (1.00 mL) in a flame-dry Schlenk tube was added trifluoromethanesulfonic anhydride (40.7 µL, 0.240 mmol, 1.20 equiv.) dropwise under argon at 0 °C. After stirring for 15 min at 0 °C, a solution of *tert*-butanesulfinamide (48.5 mg, 0.40 mmol, 2.00 equiv.) in dichloromethane (1.00 mL) was added and the reaction was stirred at 25 °C for 24 hours. After that, a 4M HCl solution in dioxane (0.500 mL, 2.00 mmol, 10.00 equiv.) was added and the biphasic mixture was vigorously stirred for 16 hours at 25 °C. The reaction was diluted with dichloromethane (ca. 3.00 mL) and carefully quenched with saturated aqueous potassium carbonate solution (ca. 3.00 mL) and extracted 3 x 5.00 mL with dichloromethane. The combined organic layers were dried over anhydrous Na_2SO_4, filtered and the solvent was removed under reduced pressure. Purification by column chromatography gave the pure product.

2-Amino-*N,N*-dimethyl-4-phenylbutanamide (4a)

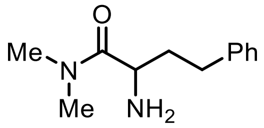

The title compound was obtained in 68% yield as a light-yellow oil.

^{1}H NMR (400 MHz, CDCl$_3$) δ 7.33 – 7.26 (m, 2H), 7.24 – 7.17 (m, 3H), 3.68 (dd, *J* = 8.5, 4.2 Hz, 1H), 2.95 (s, 3H), 2.87 – 2.70 (m, 5H), 2.21 (s, 2H), 1.97 – 1.87 (m, 1H), 1.82 – 1.72 (m, 1H).

^{13}C NMR (101 MHz, CDCl$_3$) δ 175.1, 141.3, 128.5 (2C), 128.4 (2C), 126.0, 50.1, 36.6, 36.5, 35.8, 31.8.

IR (neat) ν: 1640, 1603, 1496, 1400, 1367, 1137, 754, 701 cm^{-1}.

HRMS (ESI$^+$): exact mass calculated for $[M+H]^+$ ($C_{12}H_{19}N_2O^+$) requires m/z 207.1492, found m/z 207.1492.

2-Amino-3-phenyl-1-(pyrrolidin-1-yl)propan-1-one (4b)

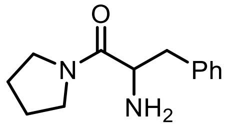

The title compound was obtained in 60% yield as a light-yellow oil.

^{1}H NMR (400 MHz, CDCl$_3$) δ 7.34 – 7.24 (m, 5H), 3.88 (t, *J* = 7.3 Hz, 1H), 3.41 – 3.36 (m, 2H), 3.04 – 2.91 (m, 2H), 2.81 – 2.66 (m, 4H), 1.92 – 1.58 (m, 4H).

^{13}C NMR (101 MHz, CDCl$_3$) δ 172.1, 137.3, 129.3 (2C), 128.4 (2C), 126.8, 54.8, 46.0, 45.8, 42.1, 25.8, 24.0.

IR (neat) ν: 1737, 1725, 1633, 1496, 1453, 1202, 841, 752, 742, 702, 522, 412 cm^{-1}.

HRMS (ESI$^+$): exact mass calculated for [M+H]$^+$ ($C_{13}H_{19}N_2O^+$) requires m/z 219.1492, found m/z 219.1493.

2-Amino-3-phenyl-1-(piperidin-1-yl)propan-1-one (4c)

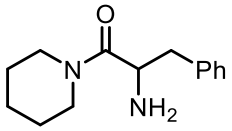

The title compound was obtained in 58% yield as a light-yellow solid.

^{1}H NMR (400 MHz, CDCl$_3$) δ 7.40 – 7.32 (m, 2H), 7.29 – 7.24 (m, 3H), 4.10 – 3.97 (m, 1H), 3.67 – 3.48 (m, 2H), 3.36 – 3.29 (m, 1H), 3.22 – 3.12 (m, 1H), 3.01 – 2.96 (m, 1H), 2.85 – 2.80 (m, 1H), 2.09 (br s, 2H), 1.63 – 1.43 (m, 5H), 1.25 – 1.10 (m, 1H).

^{13}C NMR (151 MHz, CDCl$_3$) δ 172.9, 137.9, 129.5 (2C), 128.6 (2C), 126.8, 52.4, 46.3, 43.2, 42.9, 26.2, 25.5, 24.5.

IR (neat) ν: 3363, 3059, 3025, 2969, 2854, 1738, 1630, 1443, 1366, 1217, 851, 699, 515 cm^{-1}.

HRMS (ESI$^+$): exact mass calculated for [M+H]$^+$ ($C_{14}H_{21}N_2O^+$) requires m/z 233.1648, found m/z 233.1649.

2-Amino-1-morpholino-4-phenylbutan-1-one (4d)

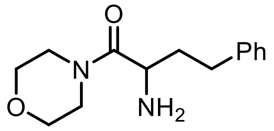

The title compound was obtained in 53% yield as a light-yellow oil.

^{1}H NMR (700 MHz, CDCl$_3$) δ 7.28 (t, *J* = 7.6 Hz, 2H), 7.20 (dd, *J* = 7.4, 4.0 Hz, 3H), 3.70 – 3.59 (m, 3H), 3.58 – 3.47 (m, 4H), 3.22 – 3.14 (m, 1H), 3.14 – 3.08 (m, 1H), 2.83 – 2.79 (m, 1H), 2.76 – 2.71 (m, 1H), 1.89 (brs, 2H), 1.88 – 1.81 (m, 1H), 1.75 (dtd, *J* = 13.8, 8.3, 5.4 Hz, 1H).

^{13}C NMR (176 MHz, CDCl$_3$) δ 174.4, 141.4, 128.7 (2C), 128.6 (2C), 126.3, 67.0, 66.6, 49.9, 45.5, 42.5, 37.4, 32.0.

IR (neat) ν: 3364, 3286, 2922, 2855, 1646, 1454, 1270, 1031, 848, 756, 701 cm^{-1}.

HRMS (ESI$^+$): exact mass calculated for [M+Na]$^+$ ($C_{14}H_{21}N_2O_2^+$) requires m/z 249.1598, found m/z 249.1594.

2-Amino-*N*-methoxy-*N*-methyl-4-phenylbutanamide (4e)

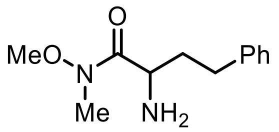

The title compound was obtained in 61% yield as a light-yellow oil.

^{1}H NMR (600 MHz, CDCl$_3$) δ 7.28 (t, *J* = 7.6 Hz, 2H), 7.21 (d, *J* = 7.3 Hz, 2H), 7.18 (t, *J* = 7.3 Hz, 1H), 3.72 (s, 1H), 3.53 (s, 3H), 3.17 (s, 3H), 2.81 (ddd, *J* = 14.1, 9.0, 5.3 Hz, 1H), 2.73 (dt, *J* = 13.9, 8.2 Hz, 1H), 2.01 (ddt, *J* = 17.1, 8.6, 5.3 Hz, 1H), 1.95 (brs, 2H), 1.82 – 1.72 (m, 1H).

^{13}C NMR (151 MHz, CDCl$_3$) δ 176.5 (*can only be seen on HMBC*), 141.6, 128.6 (2C), 128.5 (2C), 126.6, 61.4, 50.6, 36.2, 32.5, 32.2.

All analytical data were in good accordance with those reported in the literature.[15]

2-Amino-1-(indolin-1-yl)-3-(naphthalen-1-yl)propan-1-one (4f)

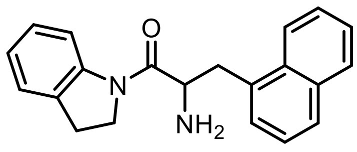

The title compound was obtained in 82% yield as a yellow solid.

^{1}H NMR (600 MHz, CDCl$_3$) δ 8.26 (d, *J* = 8.1 Hz, 1H), 8.11 (d, *J* = 8.3 Hz, 1H), 7.85 (d, *J* = 8.0 Hz, 1H), 7.73 (d, *J* = 7.9 Hz, 1H), 7.53 – 7.46 (m, 2H), 7.41 – 7.31 (m, 2H), 7.19 (t, *J* = 7.7 Hz, 1H), 7.07 (d, *J* = 7.3 Hz, 1H), 7.00 (t, *J* = 7.4 Hz, 1H), 4.07 (t, *J* = 7.1 Hz, 1H), 3.83 (td, *J* = 10.0, 7.0 Hz, 1H), 3.55 – 3.43 (m, 2H), 3.08 (td, *J* = 10.1, 6.2 Hz, 1H), 2.89 – 2.85 (m, 1H), 2.66 – 2.58 (m, 1H), 2.04 (s, 2H).

^{13}C NMR (151 MHz, CDCl$_3$) δ 173.2, 142.5, 133.7, 133.4, 132.0, 131.4, 128.9, 127.7, 127.6, 127.4, 126.3, 125.7, 125.5, 124.4, 124.0, 123.3, 117.5, 54.7, 47.4, 39.7, 27.8.

IR (neat) ν: 1649, 1596, 1480, 1461, 1368, 1339, 1316, 1288, 1262, 798, 754 cm^{-1}.

HRMS (ESI$^+$): exact mass calculated for [M+H]$^+$ ($C_{21}H_{21}N_2O^+$) requires m/z 317.1648, found m/z 317.1646.

2-Amino-*N,N*-dimethylpentanamide (4g)

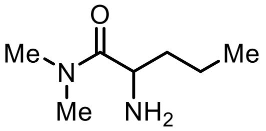

The title compound was obtained in 58% yield as a light-yellow oil.

^{1}H NMR (400 MHz, CDCl$_3$) δ 5.19 (brs, 2H), 4.40 (t, J = 6.1 Hz, 1H), 3.08 (s, 3H), 2.98 (s, 3H), 1.87 – 1.77 (m, 2H), 1.55 – 1.42 (m, 2H), 0.98 – 0.94 (m, 3H).

^{13}C NMR (151 MHz, CDCl$_3$) δ 169.7, 50.7, 37.4, 36.2, 33.3, 18.2, 13.9.

IR (neat) ν: 3258, 2962, 2874, 1655, 1506, 1405, 1260, 1218, 1155, 1065, 1026, 748, 692 cm^{-1}.

HRMS (ESI$^+$): exact mass calculated for [M+H]$^+$ ($C_7H_{17}N_2O^+$) requires m/z 145.1335, found m/z 145.1338.

2-Amino-4,4,4-trifluoro-*N,N*-dimethylbutanamide (4h)

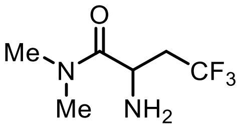

The title compound was obtained in 76% yield as a light-yellow oil.

^{1}H NMR (600 MHz, CDCl$_3$) δ 4.06 (dd, *J* = 7.4, 5.3 Hz, 1H), 3.08 (s, 3H), 2.97 (s, 3H), 2.56 – 2.44 (m, 1H), 2.42 – 2.25 (m, 1H), 1.83 (s, 2H).

^{13}C NMR (151 MHz, CDCl$_3$) δ 172.7, 126.1 (q, *J* = 277.1 Hz), 45.9 (q, *J* = 2.4 Hz), 39.8 (q, *J* = 27.0 Hz), 36.9, 36.0.

^{19}F NMR (565 MHz, CDCl$_3$) δ -63.9 (t, *J* = 10.9 Hz).

IR (neat) ν: 1639, 1376, 1253, 1224, 1136, 1088, 1032, 640 cm^{-1}.

HRMS (ESI$^+$): exact mass calculated for [M+H]$^+$ ($C_6H_{12}F_3N_2O^+$) requires m/z 185.0896, found m/z 185.0896.

2-Amino-*N,N*-dimethylundec-10-enamide (4i)

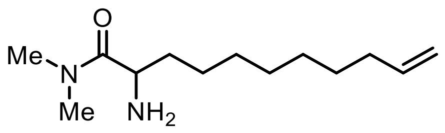

The title compound was obtained in 66% yield as a yellow solid.

^{1}H NMR (700 MHz, CDCl$_3$) δ 5.79 (ddt, *J* = 16.9, 10.2, 6.7 Hz, 1H), 5.02 – 4.86 (m, 2H), 3.07 – 3.01 (m, 4H), 2.96 (s, 3H), 2.05 – 1.98 (m, 2H), 1.70 – 1.61 (m, 1H), 1.56 – 1.49 (m, 1H), 1.43 (d, *J* = 5.9 Hz, 1H), 1.38 – 1.23 (m, 11H).

^{13}C NMR (176 MHz, CDCl$_3$) δ 174.3, 139.1, 114.1, 58.9, 36.9, 35.9, 33.7, 29.4, 29.3, 29.0 (2C), 28.8, 25.6.

IR (neat) ν: 2925, 2854, 1642, 1502, 1462, 1401, 1366, 1123, 910, 769 cm^{-1}.

HRMS (ESI$^+$): exact mass calculated for [M+H]$^+$ ($C_{13}H_{27}N_2O^+$) requires m/z 227.2118, found m/z 227.2121.

(*Z*)-2-Amino-1-(Pyrrolidin-1-yl)octadec-9-en-1-one (4j)

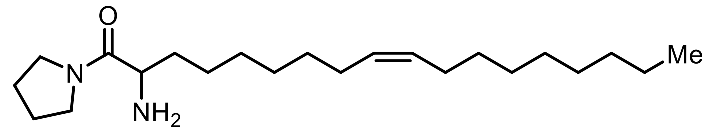

The title compound was obtained in 78% yield as a light-yellow oil.

^{1}H NMR (600 MHz, CDCl$_3$) δ 5.45 – 5.21 (m, 2H), 4.40 – 3.98 (m, 3H), 3.84 – 3.76 (m, 1H), 3.64 – 3.60 (m, 1H), 3.55 – 3.51 (m, 1H), 3.44 – 3.33 (m, 2H), 2.00 – 1.93 (m, 6H), 1.92 – 1.82 (m, 3H), 1.74 – 1.59 (m, 2H), 1.32 – 1.24 (m, 18H), 0.87 (t, *J* = 7.0 Hz, 3H).

^{13}C NMR (150 MHz, CDCl$_3$) δ 171.6, 130.0, 129.6, 52.7, 46.4, 46.2, 33.6, 31.9, 29.9, 29.7 (2C), 29.5, 29.4, 29.3, 29.2, 29.1, 27.2, 27.1, 26.0, 25.4, 24.0, 22.6.

IR (neat) ν: 2923, 2853, 1644, 1365, 1255, 1226, 1218, 1164, 770 cm^{-1}.

HRMS (ESI$^+$): exact mass calculated for [M+H]$^+$ ($C_{22}H_{43}N_2O^+$) requires m/z 351.3370, found m/z 351.3373.

2-Amino-1-(indolyn-1-yl)hex-5-yn-1-one (4k)

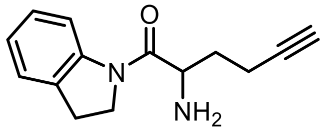

The title compound was obtained in 44% yield as a brown solid.

^{1}H NMR (600 MHz, CDCl$_3$) δ 8.24 (d, *J* = 8.0 Hz, 1H), 7.22 – 7.19 (m, 2H), 7.04 (t, *J* = 7.4, 1H), 4.18 (t, *J* = 8.4 Hz, 2H), 3.83 (dd, *J* = 8.9, 3.8 Hz, 1H), 3.22 (t, *J* = 8.4 Hz, 2H), 2.59-2.47 (m, 1H), 2.41 – 2.31 (m, 1H), 2.02 – 2.00 (m, 1H), 1.97 – 1.88 (m, 1H), 1.74 – 1.62 (m, 3H).

^{13}C NMR (151 MHz, CDCl$_3$) δ 173.9, 143.0, 131.4, 127.7, 124.7, 124.2, 117.5, 83.8, 69.4, 52.6, 47.6, 33.8, 28.3, 15.4.

IR (neat) ν: 3296, 2970, 2948, 2857, 1735, 1651, 1482, 1248, 1044, 757, 653, 639, 487 cm^{-1}.

HRMS (ESI$^+$): exact mass calculated for [M+H]$^+$ ($C_{14}H_{17}N_2O^+$) requires m/z 229.1335, found m/z 229.1330.

4-(Allyloxy)-2-amino-1-(pyrrolidin-1-yl)butan-1-one (4l)

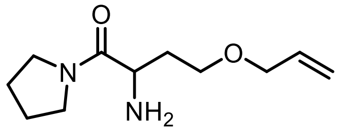

The title compound was obtained in 42% yield as a yellow oil.

^{1}H NMR (400 MHz, CDCl$_3$) δ 5.84 (ddd, *J* = 22.5, 10.7, 5.5 Hz, 1H), 5.25 (d, *J* = 17.2, 1H), 5.16 (d, *J* = 10.4 Hz, 1H), 3.97 (d, *J* = 5.1 Hz, 2H), 3.86 (br s, 1H), 3.75 – 3.61 (m, 1H), 3.55 – 3.37 (m, 5H), 3.32 (br s, 2H), 2.04 – 1.71 (m, 6H).

^{13}C NMR (151 MHz, CDCl$_3$) δ 172.7, 134.8, 117.0, 72.1, 67.0, 50.6, 46.3, 46.1, 34.6, 26.1, 24.3.

IR (neat) ν: 2952, 2923, 2871, 1631, 1448, 1343, 1098, 870 cm^{-1}.

HRMS (ESI$^+$): exact mass calculated for [M+H]$^+$ ($C_{11}H_{21}O_2N_2^+$) requires m/z 213.1598, found m/z 213.1593.

2-Amino-*N,N*-dimethyl-10-oxoundecanamide (4m)

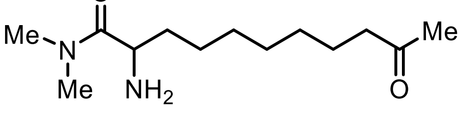

The title compound was obtained in 38% yield as a light-yellow oil.

^{1}H NMR (400 MHz, CDCl$_3$) δ 3.72 – 3.63 (m, 1H), 3.02 (s, 3H), 2.95 (s, 3H), 2.39 (t, *J* = 7.4 Hz, 2H), 2.11 (s, 3H), 2.03 (brs, 2H), 1.59 – 1.50 (m, 2H), 1.47 – 1.37 (m, 2H), 1.31 – 1.26 (m, 8H).

^{13}C NMR (101 MHz, CDCl$_3$) δ 209.4, 175.5, 51.2, 43.8, 36.9, 35.9, 35.3, 30.0, 29.4, 29.4, 29.2, 25.8, 23.9.

IR (neat) ν: 3246, 2928, 2853, 1713, 1646, 1641, 1462, 1399, 1368 cm^{-1}.

HRMS (ESI$^+$): exact mass calculated for [M+H]$^+$ ($C_{13}H_{27}N_2O_2^+$) requires m/z 243.2067, found m/z 243.2073.

Methyl 8-amino-9-(indolin-1-yl)-9-oxononanoate (4n)

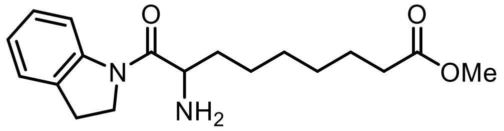

The title compound was obtained in 56% yield as a brown oil.

^{1}H NMR (600 MHz, CDCl$_3$) δ 8.23 (d, *J* = 8.1 Hz, 1H), 7.21-7.17 (m, 2H), 7.05-6.97 (m, 1H), 4.17 (dd, *J* = 17.5, 9.2 Hz, 1H), 4.04 (dd, *J* = 17.5, 9.2 Hz, 1H), 3.64 (s, 3H), 3.19 (t, *J* = 8.2 Hz, 1H), 2.28 (t, *J* = 7.5 Hz, 2H), 2.21 (br s, 2H), 1.92 – 1.72 (m, 1H), 1.65 – 1.58 (m, 2H), 1.57 – 1.48 (m, 2H), 1.44 – 1.38 (m, 2H), 1.36 – 1.28 (m, 5H).

^{13}C NMR (151 MHz, CDCl$_3$) δ 174.3, 174.0, 143.0, 131.4, 127.7, 124.7, 124.1, 117.4, 53.9, 51.6, 47.7, 35.3, 34.1, 29.3, 29.1, 28.2, 25.8, 24.9.

IR (neat) ν: 2969, 2929, 2855, 1734, 1652, 1480, 1366, 1216, 874, 754, 484 cm^{-1}.

HRMS (ESI$^+$): exact mass calculated for [M+H]$^+$ ($C_{18}H_{27}N_2O_3^+$) requires m/z 319.2016, found m/z 319.2017.

2-Amino-6-cyano-*N,N*-dimethylhexanamide (4o)

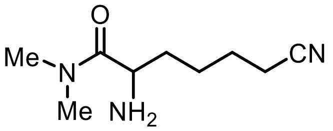

The title compound was obtained in 77% yield as a light-yellow oil.

^{1}H NMR (600 MHz, CDCl$_3$) δ 4.53 (t, *J* = 5.9 Hz, 1H), 3.09 (s, 3H), 2.98 (s, 3H), 2.47 – 2.40 (m, 2H), 1.93 (d, *J* = 6.0 Hz, 2H), 1.79 – 1.60 (m, 2H), 0.87 (t, *J* = 7.0 Hz, 2H).

^{13}C NMR (151 MHz, CDCl$_3$) δ 169.3, 119.8, 50.5, 37.4, 36.2, 30.3, 25.1, 23.8, 17.0.

IR (neat) ν: 2967, 2922, 2853, 2320, 1739, 1716, 1652, 1508, 1461, 1398, 1377 cm^{-1}.

HRMS (ESI$^+$): exact mass calculated for $[M+H]^+$ ($C_9H_{18}N_3O^+$) requires m/z 184.1444, found m/z 184.1439.

2-Amino-6-(1,3-dioxoisondolin-2-yl)-*N*,*N*-dimethylhexanamide (4p)

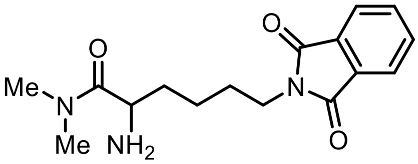

The title compound was obtained in 59% yield as a yellow oil.

^{1}H NMR (600 MHz, CDCl$_3$) δ 7.82 (dd, *J* = 5.4, 3.0 Hz, 2H), 7.69 (dd, *J* = 5.4, 3.1 Hz, 2H), 3.67 (t, *J* = 7.2 Hz, 2H), 3.43 (s, 1H), 3.02 (s, 3H), 2.93 (s, 3H), 2.14 (br s, 2H), 1.77 – 1.57 (m, 3H), 1.57 – 1.35 (m, 3H).

^{13}C NMR (151 MHz, CDCl$_3$) δ 175.3, 168.5 (2C), 134.0 (2C), 132.2 (2C), 123.3 (2C), 50.9, 37.7, 36.9, 35.9, 34.7, 28.5, 23.0.

IR (neat) ν: 3284, 2969, 2931, 2860, 1738, 1709, 1628, 1396, 957, 720, 529 cm^{-1}.

HRMS (ESI$^+$): exact mass calculated for $[M+H]^+$ ($C_{16}H_{22}N_3O_3^+$) requires m/z 304.1656, found m/z 304.1652.

2-Amino-*N*,*N*-dimethyl-3-(thiophen-2-yl)propanamide (4q)

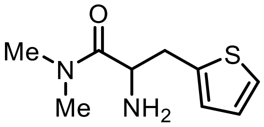

The title compound was obtained in 76% yield as a brown oil.

^{1}H NMR (700 MHz, CDCl$_3$) δ 7.17 (dd, *J* = 5.1, 1.0 Hz, 1H), 6.94 (dd, *J* = 5.1, 3.4 Hz, 1H), 6.85 (d, *J* = 2.8 Hz, 1H), 3.91 (dd, *J* = 7.8, 5.9 Hz, 1H), 3.16 (dd, *J* = 14.6, 5.8 Hz, 1H), 2.99 – 2.96 (m, 1H), 2.95 (s, 3H), 2.91 (s, 3H), 1.99 (brs, 2H).

^{13}C NMR (176 MHz, CDCl$_3$) δ 174.3, 140.2, 127.1, 126.3, 124.5, 53.3, 36.9, 36.8, 35.9.

IR (neat) ν: 3364, 3291, 2922, 1632, 1558, 1399, 1258, 1153, 1031, 851, 828, 697, 638 cm^{-1}.

HRMS (ESI$^+$): exact mass calculated for $[M+H]^+$ ($C_9H_{15}N_2OS^+$) requires m/z 199.0900, found m/z 199.0892.

2-Amino-3-(benzo[*b*]thiophen-2-yl)-*N,N*-dimethylpropanamide (4r)

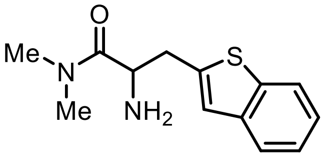

The title compound was obtained in 78% yield as a brown oil.

^{1}H NMR (600 MHz, CDCl$_3$) δ 7.76 (d, *J* = 7.9 Hz, 1H), 7.68 (d, *J* = 7.8 Hz, 1H), 7.34 – 7.29 (m, 1H), 7.29 – 7.25 (m, 1H), 7.08 (s, 1H), 4.02 (dd, *J* = 7.3, 6.1 Hz, 1H), 3.24 (dd, *J* = 14.6, 5.5 Hz, 1H), 3.03 (dd, *J* = 14.6, 8.0 Hz, 1H), 2.96 (s, 6H), 1.98 (s, 2H).

^{13}C NMR (151 MHz, CDCl$_3$) δ 173.8, 141.1, 139.8, 139.7, 124.2, 123.8, 123.0, 122.8, 122.1, 52.6, 37.4, 36.9, 35.8.

IR (neat) ν: 1641, 1498, 1457, 1366, 1257, 1133, 1111, 860, 827, 748, 727 cm^{-1}.

HRMS (ESI$^+$): exact mass calculated for [M+H]$^+$ ($C_{13}H_{17}N_2SO^+$) requires m/z 249.1056, found m/z 249.1056.

2-Amino-1-(5-methoxyindolin-1-yl)-3-phenylpropan-1-one (4s)

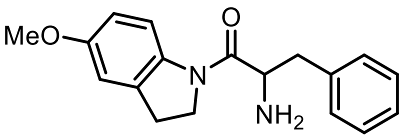

The title compound was obtained in 56% yield as an orange solid.

^{1}H NMR (600 MHz, CDCl$_3$) δ 8.17 (d, *J* = 8.7 Hz, 1H), 7.35 – 7.15 (m, 5H), 6.77 – 6.65 (m, 2H), 4.04 (td, *J* = 10.0, 7.5, 1H), 3.90 – 3.85 (m, 1H), 3.77 (s, 3H), 3.52 (td, *J* = 10.1, 6.4 Hz, 1H), 3.11 – 2.99 (m, 2H), 2.94 – 2.77 (m, 2H), 2.15 (br s, 2H).

^{13}C NMR (151 MHz, CDCl$_3$) δ 172.1, 156.7, 137.6, 136.4, 133.2, 129.5 (2C), 128.7 (2C), 127.0, 118.2, 112.0, 110.9, 60.5, 55.7, 47.9, 42.7, 28.3.

IR (neat) ν: 3061, 3026, 2923, 2854, 1641, 1486, 1263, 1030, 726, 699 cm^{-1}.

HRMS (ESI$^+$): exact mass calculated for [M+H]$^+$ ($C_{18}H_{21}N_2O_2^+$) requires m/z 297.1598, found m/z 297.1602.

2-Amino-1-(indolin-1-yl)-3-(4-(trifluoromethyl)phenyl)propan-1-one (4t)

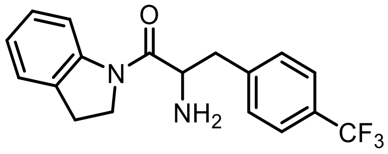

The title compound was obtained in 80% yield as a brown solid.

^{1}H NMR (400 MHz, CDCl$_3$) δ 8.26 (d, *J* = 7.9 Hz, 1H), 7.54 (d, *J* = 7.8 Hz, 2H), 7.36 (d, *J* = 7.8 Hz, 2H), 7.25 – 7.15 (m, 2H), 7.04 (t, *J* = 7.4, 1H), 4.11 (dd, *J* = 16.7, 9.9, 1H), 3.87 (br s, 1H), 3.68 (dd, *J* = 16.4, 10.0 Hz, 1H), 3.19 – 3.07 (m, 2H), 3.03 – 2.75 (m, 2H), 1.87 (br s, 2H).

^{13}C NMR (151 MHz, CDCl$_3$) δ 172.6, 142.6, 141.8, 131.3, 129.7 (2C), 129.2 (q, *J* = 32.4 Hz), 127.6, 125.5 (2C), 124.6, 124.3, 124.2 (q, *J* = 272.1 Hz), 117.4, 55.4, 47.6, 42.1, 28.0.

^{19}F NMR (376 MHz, CDCl$_3$) δ -64.5.

IR (neat) ν: 3283, 2997, 2969, 2924, 2854, 1738, 1635, 1481, 1321, 1123, 874, 757, 731 cm^{-1}.

HRMS (ESI$^+$): exact mass calculated for [M+H]$^+$ ($C_{18}H_{18}F_3N_2O^+$) requires m/z 335.1366, found m/z 335.1365.

2-Amino-2-cyclopropyl-1-(indolin-1-yl)ethan-1-one (4u)

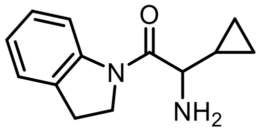

The title compound was obtained in 69% yield as an orange oil.

^{1}H NMR (600 MHz, CDCl$_3$) δ 8.23 (d, *J* = 7.9 Hz, 1H), 7.21 – 7.18 (m, 2H), 7.03 (t, *J* = 7.4, 1H), 4.20-4.05 (m, 2H), 3.49 (br s, 1H), 3.19 (t, *J* = 8.1 Hz, 2H), 2.27 (br s, 2H), 1.30 – 1.05 (m, 1H), 0.60 – 0.57 (m, 2H), 0.51 – 0.41 (m, 1H), 0.40 – 0.32 (m, 1H).

^{13}C NMR (151 MHz, CDCl$_3$) δ 172.4, 142.9, 131.6, 127.7, 124.7, 124.2, 117.5, 56.1, 47.9, 28.2, 16.0, 2.7 (2C).

IR (neat) ν: 3363, 3075, 2969, 2925, 2857, 1738, 1649, 1480, 1417, 1260, 752, 726 cm^{-1}.

HRMS (ESI$^+$): exact mass calculated for [M+H]$^+$ ($C_{13}H_{17}N_2O^+$) requires m/z 217.1335, found m/z 217.1330.

2-Amino-1-methylazacyclotridecan-2-one (4v)

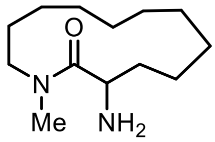

The title compound was obtained in 55% yield as a yellow solid. For simplicity the major rotamer is characterized.

^{1}H NMR (700 MHz, CDCl$_3$) δ 4.43 – 4.39 (m, 1H), 4.19 (dd, *J* = 6.8, 2.4 Hz, 1H), 3.41 – 3.29 (m, 3H), 3.05 (s, 3H), 2.58 (ddd, *J* = 13.5, 5.2, 3.0 Hz, 1H), 1.86 – 1.82 (m, 1H), 1.72 – 1.65 (m, 2H), 1.39 – 1.24 (m, 14H).

^{13}C NMR (176 MHz, CDCl$_3$) δ 173.0, 50.7, 47.8, 35.3, 31.5, 26.8, 26.4, 26.3, 25.2, 24.4, 23.5, 23.3, 19.5.

IR (neat) ν: 2928, 2859, 1643, 1491, 1462, 1376, 1277, 1254, 1223, 1119, 768, 735 cm^{-1}.

HRMS (ESI$^+$): exact mass calculated for [M+H]$^+$ ($C_{13}H_{27}N_2O^+$) requires m/z 227.2118, found m/z 227.2124.

4. General procedure for the α-amination of ketones

General Procedure D

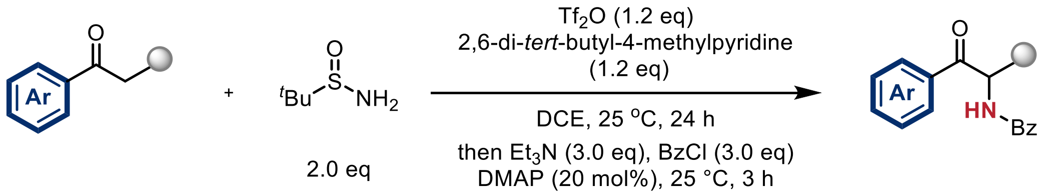

To a flame dried Schlenk tube under argon with a stirring bar and solution of a ketone (0.20 mmol, 1.00 equiv.), 2,6-di-*tert*-butyl-4-methylpyridine (1.20 equiv.) in DCE (0.10 M) was added trifluoromethanesulfonic anhydride (1.20 equiv.) at 25 °C. The reaction mixture was stirred at room temperature for 30 min (for 4-methoxyacetophenone as a substrate) or other time stated. To the reaction mixture at room temperature was then added the *tert*-butanesulfinamide (48.5 mg, 0.40 mmol, 2.00 equiv.) in DCE (1.00 mL). The reaction mixture was stirred for 14 h at room temperature. Triethylamine (84.0 µL, 0.60 mmol, 3.00 equiv.), 4-dimethylaminopyridine (DMAP, 5.0 mg, 0.04 mmol, 0.20 equiv.) and benzoyl chloride (70.0 µL, 0.60 mmol, 3.00 equiv.) were then added in sequence. The reaction was stirred at room temperature for another 3 h before the addition of a saturated aqueous solution of sodium bicarbonate and extracted 3 x 5 mL with dichloromethane. The combined organic layers were dried over anhydrous magnesium sulfate, the dried solution was filtered and the filtrate was concentrated under reduced pressure to afford the crude product. The crude product was then purified by column to give the desired aminated ketones.

N-(2-(4-Methoxyphenyl)-2-oxoethyl)benzamide (5a)

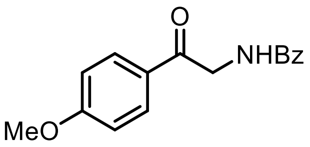

The title compound was obtained in 63% yield as a yellow solid. The activation time is 30 min.

^{1}H NMR (600 MHz, $CDCl_3$) δ 8.00 (d, *J* = 8.8 Hz, 2H), 7.88 (d, *J* = 7.5 Hz, 2H), 7.52 (t, *J* = 7.3 Hz, 1H), 7.45 (t, *J* = 7.6 Hz, 2H), 7.36 (s, 1H), 6.97 (d, *J* = 8.8 Hz, 2H), 4.89 (d, *J* = 4.1 Hz, 2H), 3.88 (s, 3H).

^{13}C NMR (151 MHz, $CDCl_3$) δ 192.6, 167.3, 164.3, 133.9, 131.6, 130.3 (2C), 128.6 (2C), 127.3, 127.1 (2C), 114.1 (2C), 55.5, 46.4.

IR (neat) ν: 3359, 2927, 1688, 1652, 1601, 1514, 1487, 1360, 1310, 1235, 1174, 712 cm^{-1}.

HRMS (ESI$^+$): exact mass calculated for $[M+H]^+$ ($C_{16}H_{16}NO_3^+$) requires m/z 270.1125, found m/z 270.1124.

N-(2-(3,4-Dimethoxyphenyl)-2-oxoethyl)benzamide (5b)

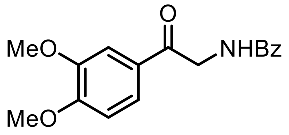

The title compound was obtained in 47% yield as a brown solid. The activation time is 30 min.

^{1}H NMR (400 MHz, CDCl$_3$) δ 7.89 – 7.87 (m, 2H), 7.68 (dd, *J* = 8.4, 2.0 Hz, 1H), 7.58 – 7.50 (m, 2H), 7.47 (t, *J* = 7.3 Hz, 2H), 7.33 (s, 1H), 6.94 (d, *J* = 8.4 Hz, 1H), 4.92 (d, *J* = 4.1 Hz, 2H), 3.97 (s, 3H), 3.96 (s, 3H).

^{13}C NMR (176 MHz, CDCl$_3$) δ 192.7, 167.4, 154.2, 149.3, 133.9, 131.7, 128.6 (2C), 127.5, 127.1 (2C), 122.7, 110.4, 109.9, 56.2, 56.1, 46.4.

IR (neat) ν: 3350, 1686, 1596, 1515, 1463, 1420, 1264, 1148, 1022, 765, 712 cm^{-1}.

HRMS (ESI$^+$): exact mass calculated for [M+H]$^+$ ($C_{17}H_{18}NO_4^+$) requires m/z 300.1230, found m/z 300.1230.

N-(2-(2,3-Dihydrobenzo[*b*][1,4]dioxin-6-yl)-2-oxoethyl)benzamide (5c)

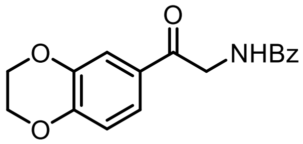

The title compound was obtained in 52% yield as a yellow solid. The activation time is 7 min.

^{1}H NMR (400 MHz, CDCl$_3$) δ 7.92 – 7.85 (m, 2H), 7.60 – 7.50 (m, 3H), 7.46 (t, *J* = 7.4 Hz, 2H), 7.30 (s, 1H), 6.96 (d, *J* = 9.1 Hz, 1H), 4.88 (d, *J* = 4.1 Hz, 2H), 4.38 – 4.25 (m, 4H).

^{13}C NMR (176 MHz, CDCl$_3$) δ 192.6, 167.4, 149.0, 143.7, 134.0, 131.7, 128.6 (2C), 128.1, 127.1 (2C), 122.2, 117.7, 117.5, 64.8, 64.1, 46.6.

IR (neat) ν: 3380, 1689, 1649, 1604, 1580, 1488, 1460, 1322, 1259, 1065, 894, 712, 693 cm^{-1}.

HRMS (ESI$^+$): exact mass calculated for [M+H]$^+$ ($C_{17}H_{16}NO_4^+$) requires m/z 298.1074, found m/z 298.1071.

N-(2-Oxo-2-(3,4,5-trimethoxyphenyl)ethyl)benzamide (5d)

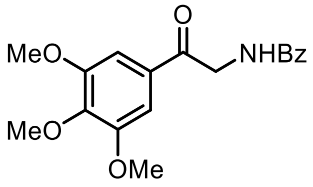

The title compound was obtained in 43% yield as a yellow solid. The activation time is 30 min.

^{1}H NMR (600 MHz, CDCl$_3$) δ 7.87 (d, *J* = 7.8 Hz, 2H), 7.52 (t, *J* = 7.2 Hz, 1H), 7.46 (t, *J* = 7.6, 2H), 7.35 – 7.29 (m, 1H), 7.26 (s, 2H), 4.93 (d, *J* = 4.0 Hz, 2H), 3.92 (s, 9H).

^{13}C NMR (151 MHz, CDCl$_3$) δ 193.2, 167.6, 153.5, 143.7, 134.0, 131.9, 129.6, 128.8 (2C), 127.3 (2C), 105.5 (2C), 61.1, 56.5 (2C), 46.8.

IR (neat) ν: 3359, 3064, 2940, 2839, 2250, 1737, 1686, 1414, 1358, 1123, 999, 908, 726, 539, 515 cm^{-1}.

HRMS (ESI$^+$): exact mass calculated for [M+Na]$^+$ ($C_{18}H_{19}NO_5Na^+$) requires m/z 352.1155, found m/z 352.1154.

N-(2-(3-Fluoro-4-methoxyphenyl)-2-oxoethyl)benzamide (5e)

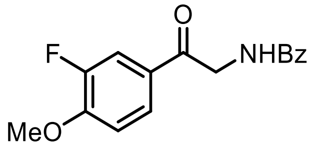

The title compound was obtained in 52% yield as a yellow solid. The activation time is 2 h.

^{1}H NMR (400 MHz, CDCl$_3$) δ 7.91 – 7.85 (m, 2H), 7.84 – 7.74 (m, 2H), 7.57 – 7.51 (m, 1H), 7.51 – 7.43 (m, 2H), 7.27 (s, 1H) 7.05 (t, *J* = 8.3 Hz, 1H), 4.89 (d, *J* = 4.2 Hz, 2H), 3.98 (s, 3H).

^{13}C NMR (101 MHz, CDCl$_3$) δ 192.0 (d, *J* = 2.0 Hz), 167.4, 152.8 (d, *J* = 10.8 Hz), 152.3 (d, *J* = 250.3 Hz), 133.9, 131.8, 128.6 (2C), 127.6 (d, *J* = 5.4 Hz), 127.1 (2C), 125.4 (d, *J* = 3.4 Hz), 115.6 (d, *J* = 19.2 Hz), 112.7, 56.4, 46.6.

^{19}F NMR (377 MHz, CDCl$_3$) δ -133.21 (dd, *J* = 11.3, 8.4 Hz).

IR (neat) ν: 3350, 1694, 1651, 1612, 1580, 1519, 1438, 1283, 1228, 1138, 1122, 763 cm^{-1}.

HRMS (ESI$^+$): exact mass calculated for [M+H]$^+$ ($C_{16}H_{15}NFO_3^+$) requires m/z 288.1030, found m/z 288.1033.

N-(2-(3-Chloro-4-methoxyphenyl)-2-oxoethyl)benzamide (5f)

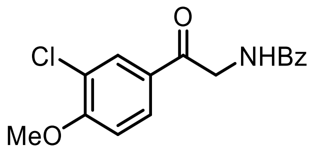

The title compound was obtained in 58% yield as a yellow solid. The activation time is 2 h.

^{1}H NMR (400 MHz, CDCl$_3$) δ 8.08 (d, *J* = 2.1 Hz, 1H), 7.95 (dd, *J* = 8.6, 2.2 Hz, 1H), 7.91 – 7.85 (m, 2H), 7.57 – 7.50 (m, 1H), 7.50 – 7.43 (m, 2H), 7.26 (s, 1H), 7.02 (d, *J* = 8.7 Hz, 1H), 4.90 (d, *J* = 4.2 Hz, 2H), 4.00 (s, 3H).

^{13}C NMR (101 MHz, $CDCl_3$) δ 191.9, 167.4, 159.7, 133.9, 131.8, 130.3, 128.6 (2C), 128.5, 127.9, 127.1 (2C), 123.6, 111.6, 56.5, 46.5.

IR (neat) ν: 3350, 1693, 1650, 1594, 1534, 1504, 1488, 1261, 1211, 1061, 1012, 710 cm^{-1}.

HRMS (ESI$^+$): exact mass calculated for $[M+H]^+$ ($C_{16}H_{15}N^{35}ClO_3^+$) requires m/z 304.0735, found m/z 304.0736.

5. Free α-amination/functionalization of amides

5.1. α-Amination/peptide coupling

General Procedure E

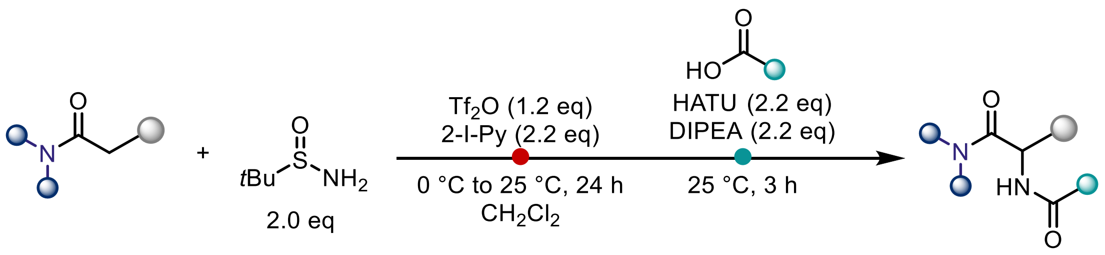

To a mixture of amide (0.20 mmol, 1.00 equiv.), 2-iodopyridine (46.8 µL, 0.440 mmol, 2.20 equiv.) in dichloromethane (1.00 mL) in a flame-dry Schlenk tube was added trifluoromethanesulfonic anhydride (40.7 µL, 0.240 mmol, 1.20 equiv.) dropwise under argon at 0 °C. After stirring for 15 min at 0 °C, a solution of *tert*-butylsulfinamide (48.5 mg, 0.40 mmol, 2.00 equiv.) in dichloromethane (1.00 mL) was added and the reaction stirred at 25 °C for 24 hours. After that, carboxylic acid (2.00 equiv.), HATU (2.20 equive.) and DIPEA (2.20 equiv.) were solubilized with dichloromethane (5.00 mL) at 0 °C in another flask. The reaction mixture in the Schlenk tube was transferred to the activated carboxylic acid mixture in one-portion at 0 °C. The reaction was stirred at room temperature for another 3 h before the addition of a saturated aqueous solution of sodium bicarbonate and extracted 3 x 5.00 mL with dichloromethane. The combined organic layers were dried over anhydrous magnesium sulfate, the dried solution was filtered and the filtrate was concentrated under reduced pressure to afford the crude product. The crude product was then purified by column to give the desired peptides.

***tert*-Butyl ((2*R*)-1-((1-(dimethylamino)-1-oxo-4-phenylbutan-2-yl)amino)-1-oxo-3-phenylpropan-2-yl)carbamate (6a)**

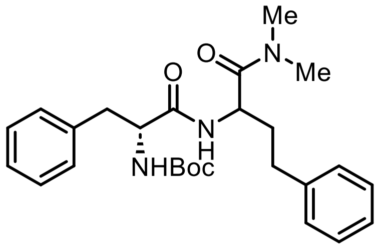

The title compound was obtained in 83% yield as a white solid. The *d.r.* was determined by crude NMR as 1:1.

^{1}H NMR (700 MHz, CD_3OD) δ 7.27 – 7.14 (m, 10H), 4.78 – 4.71 (m, 1H), 4.37 – 4.34 (m, 1H), 3.16 – 3.06 (m, 1H), 2.98 – 2.83 (m, 8H), 2.61 – 2.72 (m, 1H), 2.55 – 2.43 (m, 1H), 1.98 – 1.79 (m, 2H), 1.38 (2 x s, 9H). *The N-H was not observed in ^{1}H NMR.

^{13}C NMR (176 MHz, CD_3OD) δ 174.1, 173.4, 157.6, 143.3, 138.6, 130.4 (2C), 129.7, 129.5 (4C), 129.4 (2C), 127.8, 127.2, 127.0, 80.7, 57.3, 49.7, 38.9, 37.4, 36.1, 34.7, 32.6, 28.7. *For simplicity one of the diastereomers is characterized.

IR (neat) ν: 1710, 1635, 1497, 1392, 1366, 1251, 1168, 842, 750, 700 cm^{-1}.

HRMS (ESI$^+$): exact mass calculated for $[M+H]^+$ ($C_{26}H_{36}N_3O_4^+$) requires m/z 454.2700, found m/z 454.2692.

(9H-Fluoren-9-yl)methyl ((2*S*)-1-(((2*S*)-1-((1-(dimethylamino)-1-oxo-4-phenylbutan-2-yl)amino)-1-oxo-3-phenylpropan-2-yl)amino)-1-oxo-3-phenylpropan-2-yl)carbamate (6b)

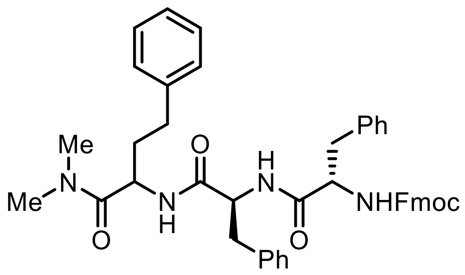

The title compound was obtained in 84% yield as a white solid. The *d.r.* was determined by crude NMR as 1:1.

The spectroscopic data is given for the 1:1 mixture of diastereoisomers.

^{1}H NMR (600 MHz, DMSO-d$_6$) δ 8.42 (d, *J* = 8.4 Hz, 0.5H, *1 dia*), 8.32 (d, *J* = 8.2 H, 0.5H, *1 dia*), 8.16 (d, *J* = 8.1 Hz, 1H), 7.90 – 7.83 (m, 2H), 7.62 – 7.53 (m, 2H), 7.43 – 7.35 (m, 2H), 7.32 – 7.09 (m, 17H), 4.72 – 4.59 (m, 2H), 4.26 – 4.06 (m, 4H), 3.05 – 3.01 (m, 1H), 2.97 – 2.92 (m, 1H), 2.90 – 2.87 (m, 1H), 2.85 (s, 1.5 H, *1 dia*), 2.82 (s, 1.5H, *1 dia*), 2.80 (s, 1.5H, *1 dia*), 2.79 (s,

1.5H, *1 dia*), 2.76 – 2.71 (m, 1H), 2.63 – 2.53 (m, 1H), 2.47 – 2.36 (m, 1H), 1.93 – 1.63 (m, 2H). *One of the N-H was not observed in ^{1}H NMR.

^{13}C NMR (151 MHz, DMSO-d$_6$) δ 171.5 (*1 dia*), 171.2 (*1 dia*), 170.8 (*1 dia*), 170.7 (*1 dia*), 170.5 (*1 dia*), 170.4 (*1 dia*), 155.7, 143.8 (2C, *1 dia*), 143.7 (2C, *1 dia*), 141.3 (4C, *2 dia*), 140.7, 138.2 (*1 dia*), 138.0 (*1 dia*), 137.6 (*1 dia*), 137.5 (*1 dia*), 129.3 (2C), 129.2 (2C), 128.4 (2C), 128.3 (2C), 128.0 (4C), 127.6 (2C), 127.1, 126.3 (2C), 126.2, 125.9, 125.3 (2C), 120.1 (2C), 65.7, 56.2 (*1 dia*), 56.1 (*1 dia*), 53.9 (*1 dia*), 53.7 (*1 dia*), 47.8 (2C, *2 dia*), 46.5, 37.5, 37.4, 36.3 (2C, *2 dia*), 35.2 (2C, *2 dia*), 33.5, 31.4 (*1 dia*), 31.1 (*1 dia*).

IR (neat) ν: 3277, 2925, 2859, 1639, 1529, 1450, 1255, 840, 738, 699, 557 cm^{-1}.

HRMS (ESI$^+$): exact mass calculated for [M+Na]$^+$ ($C_{45}H_{46}N_4O_5Na^+$) requires m/z 745.3360, found m/z 745.3370.

(9*H*-Fluoren-9-yl)methyl ((2*R*)-3-(*tert*-butylthio)-1-((1-cyclopropyl-2-(dimethylamino)-2-oxoethyl)amino)-1-oxopropan-2-yl)carbamate (6c)

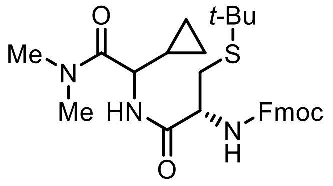

The title compound was obtained in 97% yield as a colorless wax. The *d.r.* was determined by crude NMR as 1:1.

The spectroscopic data is given for the 1:1 mixture of diastereoisomers.

^{1}H NMR (600 MHz, CD$_3$OD) δ 7.71 (d, *J* = 7.5 Hz, 2H), 7.67 – 7.62 (m, 2H), 7.36 (t, *J* = 6.7, 2H), 7.28 (td, *J* = 7.4, 1.0 Hz, 2H), 4.49 (dd, *J* = 10.4, 7.8 Hz, 1H), 4.42 – 4.38 (m, 0.5H), 4.37-4.34 (m, 0.5H), 4.33 – 4.29 (m, 2H), 4.23-4.18 (m, 1H), 3.10 (d, *J* = 10.4 Hz, 2H), 2.92 (d, *J* = 8.9 Hz, 2H), 2.79 (s, 6H), 1.30 (2 x s, 9H), 1.21-1.14 (m, 1H), 0.51 – 0.34 (m, 4H).

^{13}C NMR (151 MHz, CD$_3$OD) δ 172.8, 172.4, 158.2 (*1 dia*), 158.1 (*1 dia*), 145.2 (2C, *1 dia*), 145.1 (2C, *1 dia*), 142.5 (2C), 128.8 (2C), 128.2 (2C), 126.3 (2C, *1 dia*), 126.2 (2C, *1 dia*), 120.9 (2C), 68.2 (*1 dia*), 68.1 (*1 dia*), 56.6 (*1 dia),* 56.4 (*1 dia*), 53.4 (*1 dia*), 53.2 (*1 dia*), 48.3 (2C, *2 dia*), 38.9, 37.8 (2C, *2 dia*), 36.1, 31.6 (*1 dia*), 31.5 (*1 dia*), 31.3 (3C), 13.9, 3.4 (*1 dia*), 3.3 (*1 dia*), 3.1 (*1 dia*), 3.0 (*1 dia*).

IR (neat) ν: 3015, 2969, 1737, 1724, 1633, 1451, 1365, 973, 843 cm^{-1}.

HRMS (ESI$^+$): exact mass calculated for [M+Na]$^+$ ($C_{29}H_{37}N_3O_4SNa^+$) requires m/z 546.2397, found m/z 546.2399.

(9*H*-Fluorenyl-9-yl)methyl ((2*S*)-1-(((2*S*)-1-((2*S*)-2-((1-(dimethylamino)-1-oxo-3-(thiophen-2-yl)propan-2-yl)carbamoyl)pyrrolidin-1-yl)-1-oxopropan-2-yl)amino)-1-oxopropan-2-yl)carbamate (6d)

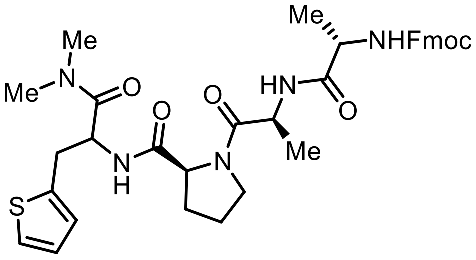

The title compound was obtained in 72% yield from the enantiopure (*R*)-*tert*-butanesulfinamide as a brown solid. The *d.r.* was determined by crude NMR as 9:1.

The spectroscopic data is given for the major diastereoisomer.

^{1}H NMR (600 MHz, CD_3OD) δ 7.79 (d, *J* = 7.4 Hz, 2H), 7.68 – 7.61 (m, 2H), 7.39 (t, *J* = 7.5, 2H), 7.31 (t, *J* = 7.3 Hz, 2H), 7.22 (d, *J* = 5.0 Hz, 1H), 6.95 – 6.85 (m, 2H), 5.09 (t, *J* = 7.1 Hz, 1H), 4.57 – 4.50 (m, 1H), 4.40 – 4.29 (m, 2H), 4.21 (t, *J* = 6.6 Hz, 1H), 4.17 – 4.10 (m, 1H), 3.81 – 3.69 (m, 1H), 3.63 – 3.55 (m, 1H), 3.26 – 3.08 (m, 4H), 2.96 (s, 3H), 2.89 (s, 3H), 2.10 – 2.06 (m, 1H), 1.94 – 1.91 (m, 2H), 1.79 – 1.69 (m, 1H), 1.36 – 1.29 (m, 6H).

^{13}C NMR (151 MHz, CD_3OD) δ 175.1, 173.5, 173.2, 172.2, 158.2, 145.4 (2C), 142.6 (2C), 139.9, 128.8, 128.2, 127.9 (2C), 127.8 (2C), 126.3, 126.2, 125.4, 120.9 (2C), 68.0, 61.4, 51.8 (2C), 48.5, 48.4, 37.6, 36.2 (2C), 33.3, 30.6, 25.9, 18.2, 17.0.

IR (neat) ν: 2969, 2924, 2852, 1655, 1624, 1227, 1056, 759 cm^{-1}.

HRMS (ESI$^+$): exact mass calculated for $[M+Na]^+$ ($C_{35}H_{41}N_5O_6SNa^+$) requires m/z 682.2670, found m/z 682.2666.

5.2. α-Amination/Pictet-Spengler cyclization

***N*,*N*-Dimethyl-1,2,3,4-tetrahydrobenzo[4,5]thieno[3,2-*c*]pyridine-3-carboxamide (7)**

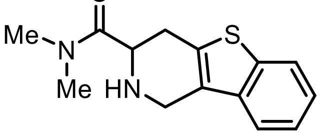

To a mixture of 3-(benzo[*b*]thiophen-2-yl)-*N*,*N*-dimethylpropanamide (**2r**, 46.7 mg, 0.20 mmol, 1.00 equiv.), 2-iodopyridine (46.8 µL, 0.44 mmol, 2.20 equiv.) in dichloromethane (1.00 mL) in

a flame-dry Schlenk tube was added trifluoromethanesulfonic anhydride (40.7 µL, 0.24 mmol, 1.20 equiv.) dropwise under argon at 0 °C. After stirring for 15 min at 0 °C, a solution of *tert*-butylsulfinamide (48.5 mg, 0.40 mmol, 2.00 equiv.) in dichloromethane (1.00 mL) was added and the reaction stirred at 25 °C for 24 hours. After that, paraformaldehyde (12.0 mg, 0.40 mmol, 2.00 equiv.) and trifluoroacetic acid (59.4 µL, 91.2 mg, 0.80 mmol, 4.00 equiv.) were added and the reaction mixture was stirred at 25 °C for 24 h. The mixture was quenched with a saturated aqueous solution of $NaHCO_3$ (3.00 mL) and extracted 3 x 5.00 mL with dichloromethane. The combined organic layers were dried over anhydrous magnesium sulfate, the dried solution was filtered and the filtrate was concentrated under reduced pressure to afford the crude product. The crude product was then purified by column chromatography. The title compound was obtained in 62% yield as a brown solid.

^{1}H NMR (600 MHz, $CDCl_3$) δ 7.78 (d, *J* = 7.9 Hz, 1H), 7.51 (d, *J* = 7.8 Hz, 1H), 7.33 (t, *J* = 7.4 Hz, 1H), 7.29 (t, *J* = 7.4 Hz, 1H), 4.24 (d, *J* = 16.6 Hz, 1H), 4.11 (d, *J* = 15.2 Hz, 1H), 4.04 (dd, *J* = 10.4, 3.8 Hz, 1H), 3.14 (s, 3H), 3.10 – 3.03 (m, 1H), 3.02 (s, 3H), 2.90 (d, *J* = 16.3 Hz, 1H). *The N-H was not observed in ^{1}H NMR.

^{13}C NMR (151 MHz, $CDCl_3$) δ 172.4, 138.1, 137.4, 134.4, 128.4, 124.3, 129.2, 122.6, 120.4, 53.6, 43.6, 37.3, 35.9, 28.7.

IR (neat) ν: 3342, 2929, 2870, 1644, 1397, 1128, 755 cm^{-1}.

HRMS (ESI$^+$): exact mass calculated for $[M+H]^+$ ($C_{14}H_{17}N_2OS^+$) requires m/z 261.1056, found m/z 261.1059.

***N*,*N*-Dimethyl-1-phenyl-1,2,3,4-tetrahydrobenzo[4,5]thieno[3,2-*c*]pyridine-3-carboxamide (8)**

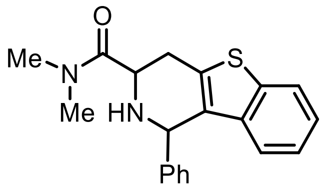

To a mixture of 3-(benzo[*b*]thiophen-2-yl)-*N*,*N*-dimethylpropanamide (**2r**, 46.7 mg, 0.20 mmol, 1.00 equiv.), 2-iodopyridine (46.8 µL, 0.44 mmol, 2.20 equiv.) in dichloromethane (1.00 mL) in a flame-dry Schlenk tube was added trifluoromethanesulfonic anhydride (40.7 µL, 0.24 mmol, 1.20 equiv.) dropwise under argon at 0 °C. After stirring for 15 min at 0 °C, a solution of *tert*-butylsulfinamide (48.5 mg, 0.400 mmol, 2.00 equiv.) in dichloromethane (1.00 mL) was added and the reaction stirred at 25 °C for 24 hours. After that, benzaldehyde (83.3 µL, 86.6 mg, 0.80 mmol, 4.00 equiv.) and trifluoroacetic acid (59.4 µL, 91.2 mg, 0.80 mmol, 4.00 equiv.) were added and the reaction mixture was stirred at 40 °C for 96 h. The mixture was quenched with a saturated aqueous solution of $NaHCO_3$ (3.00 mL) and extracted 3 x 5.00 mL with dichloromethane. The combined organic layers were dried over anhydrous magnesium sulfate,

the dried solution was filtered and the filtrate was concentrated under reduced pressure to afford the crude product. The crude product was then purified by column chromatography. The two diastereoisomers of the title compound were isolated in 29% and 30% yield.

***trans-N,N*-Dimethyl-1-phenyl-1,2,3,4-tetrahydrobenzo[4,5]thieno[3,2-*c*]pyridine-3-carboxamide (*trans*-8)**

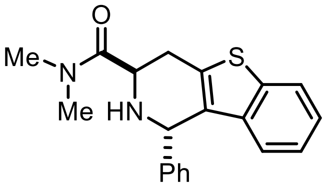

The title compound was isolated in 29% yield as a white solid.

^{1}H NMR (400 MHz, $CDCl_3$) δ 7.81 (d, *J* = 7.9 Hz, 1H), 7.32 – 7.23 (m, 6H), 7.20 – 7.14 (m, 2H), 5.37 (s, 1H), 3.89 (dd, *J* = 10.6, 4.4 Hz, 1H), 3.38 – 3.31 (m, 1H), 2.95 – 2.87 (m, 1H), 2.93 (s, 3H), 2.67 (s, 3H), 2.33 (br s, 1H).

^{13}C NMR (151 MHz, $CDCl_3$) δ 171.9, 141.6, 138.1, 137.9, 137.4, 129.7, 129.2 (2C), 128.4 (2C), 127.6, 124.3, 124.0, 122.5, 121.8, 56.4, 48.3, 36.7, 36.0, 28.6.

IR (neat) v: 2923, 2897, 2853, 1638, 1359, 1066, 701 cm^{-1}.

HRMS (ESI^+): exact mass calculated for $[M+H]^+$ ($C_{20}H_{21}N_2OS^+$) requires m/z 337.1369, found m/z 337.1366.

***cis-N,N*-Dimethyl-1-phenyl-1,2,3,4-tetrahydrobenzo[4,5]thieno[3,2-*c*]pyridine-3-carboxamide (*cis*-8)**

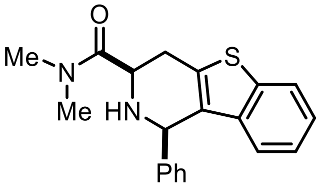

The title compound was isolated in 30% yield as a light-yellow solid.

^{1}H NMR (600 MHz, $CDCl_3$) δ 7.77 (d, *J* = 8.0 Hz, 1H), 7.32 – 7.25 (m, 5H), 7.20 (t, *J* = 7.5 Hz, 1H), 7.05 (t, *J* = 7.5 Hz, 1H), 6.85 (d, *J* = 8.0 Hz, 1H), 5.31 (s, 1H), 4.14 (d, *J* = 10.6 Hz, 1H), 3.26 – 3.19 (m, 1H), 3.17 (s, 3H), 3.04 – 2.99 (m, 1H), 2.99 (s, 3H), 1.92 (br s, 1H).

^{13}C NMR (151 MHz, $CDCl_3$) δ 171.9, 141.8, 138.1, 137.5, 136.8, 130.0, 129.0 (2C), 128.6 (2C), 128.2, 124.0, 123.9, 122.7, 122.3, 59.8, 53.8, 37.3, 35.9, 29.6.

IR (neat) v: 3025, 2969, 2922, 2851, 1639, 1366, 729 cm^{-1}.

HRMS (ESI+): exact mass calculated for $[M+H]^+$ ($C_{20}H_{21}N_2OS^+$) requires m/z 337.1369, found m/z 337.1365.

5.3. α-Amination/Reductive amination

Indolin-1-yl(6-methylpiperidin-2-yl)methanone (9)

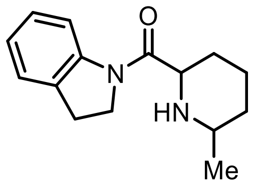

To a mixture of 1-(indolin-1-yl)heptane-1,6-dione (54.5 mg, 0.20 mmol, 1.00 equiv.), 2-iodopyridine (46.8 µL, 0.44 mmol, 2.20 equiv.) in dichloromethane (1.00 mL) in a flame-dry Schlenk tube was added trifluoromethanesulfonic anhydride (40.7 µL, 0.240 mmol, 1.20 equiv.) dropwise under argon at 0 °C. After stirring for 15 min at 0 °C, a solution of *tert*-butylsulfinamide (48.5 mg, 0.40 mmol, 2.00 equiv.) in dichloromethane (1.00 mL) was added and the reaction stirred at 25 °C for 24 hours. After that, acetic acid (11.5 µL, 12.0 mg, 0.20 mmol, 1.00 equiv.) and $NaBH(OAc)_3$ (84.8 mg, 0.40 mmol, 2.00 equiv.) were added and the reaction mixture was stirred at 25 °C for 24 h. The mixture was quenched with 1.00 M NaOH (3.00 mL) and extracted 3 x 5.00 mL with dichloromethane. The combined organic layers were dried over anhydrous magnesium sulfate, the dried solution was filtered and the filtrate was concentrated under reduced pressure to afford the crude product. The *d.r.* was determined by crude NMR as 2:1 (70% NMR yield). The crude product was then purified by column chromatography. The major diastereoisomer was isolated in 50% yield as a yellow oil.

The spectroscopic data is given for the major diastereoisomer.

^{1}H NMR (600 MHz, $CDCl_3$) δ 8.23 (d, *J* = 7.5 Hz, 1H), 7.19 – 7.15 (m, 2H), 7.00 (t, *J* = 7.4 Hz, 1H), 4.30 – 4.20 (m, 1H), 4.06 – 4.00 (m, 1H), 3.60 (d, *J* = 10.8 Hz, 1H), 3.17 (br s, 2H), 2.77 – 2.70 (m, 1H), 1.99 – 1.91 (m, 1H), 1.87 – 1.76 (m, 2H), 1.70 (d, *J* = 11.9 Hz, 1H), 1.55 – 1.51 (m, 1H), 1.44 – 1.38 (m, 1H), 1.13 (d, *J* = 10.7 Hz, 3H), 1.06 – 0.92 (m, 1H).

^{13}C NMR (151 MHz, $CDCl_3$) δ 171.8, 143.3, 131.4, 127.6, 124.6, 123.9, 117.5, 59.3, 51.4, 47.7, 34.4, 29.1, 28.2, 24.7, 22.9.

IR (neat) ν: 3267, 2970, 2925, 1648, 1365, 1216, 754 cm^{-1}.

HRMS (ESI+): exact mass calculated for $[M+H]^+$ ($C_{15}H_{21}N_2O^+$) requires m/z 245.1648, found m/z 245.1644.

5.4. α-Amination/direct cyclization

***N,N*-Dimethylpiperidine-2-carboxamide (10)**

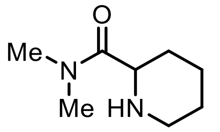

The title compound was obtained in 82% yield with 6-chloro-*N,N*-dimethylhexanamide (**2x**) using General Procedure C as a light-yellow solid.

^{1}H NMR (600 MHz, $CDCl_3$) δ 8.54 (s, 1H), 4.32 (d, *J* = 10.4 Hz, 1H), 3.67 (d, *J* = 12.4 Hz, 1H), 3.23 – 3.03 (m, 4H), 2.98 (s, 3H), 2.08 – 2.00 (m, 2H), 1.92 – 1.79 (m, 3H), 1.68 – 1.52 (m, 1H).

^{13}C NMR (151 MHz, $CDCl_3$) δ 168.7, 55.4, 43.8, 37.2, 36.0, 25.9, 21.8, 21.6.

IR (neat) ν: 1646, 1509, 1450, 1386, 1258, 1125, 1054, 1032, 911, 503 cm^{-1}.

HRMS (ESI^+): exact mass calculated for $[M+H]^+$ ($C_8H_{17}N_2O^+$) requires m/z 157.1335, found m/z 157.1333.

6. NMR Spectra

1-(Indolin-1-yl)-3-(naphthalen-1-yl)propan-1-one (2f)

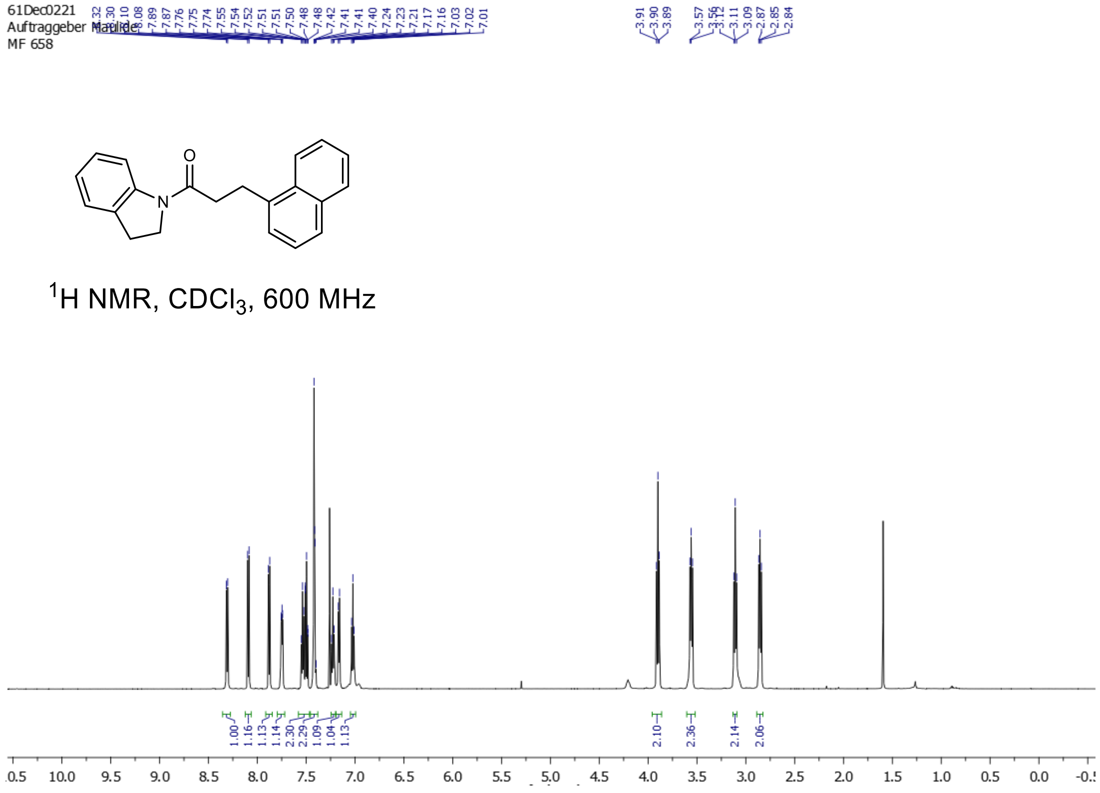

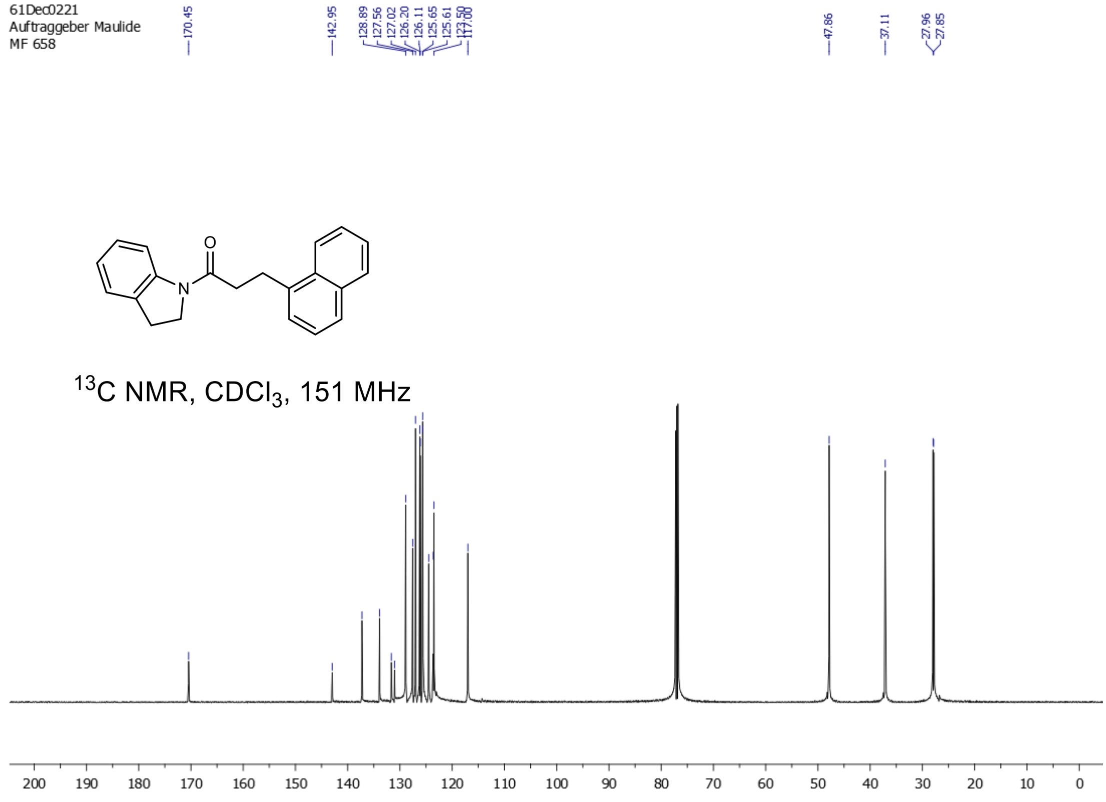

(*Z*)-1-(Pyrrolidin-1-yl)octadec-9-en-1-one (2j)

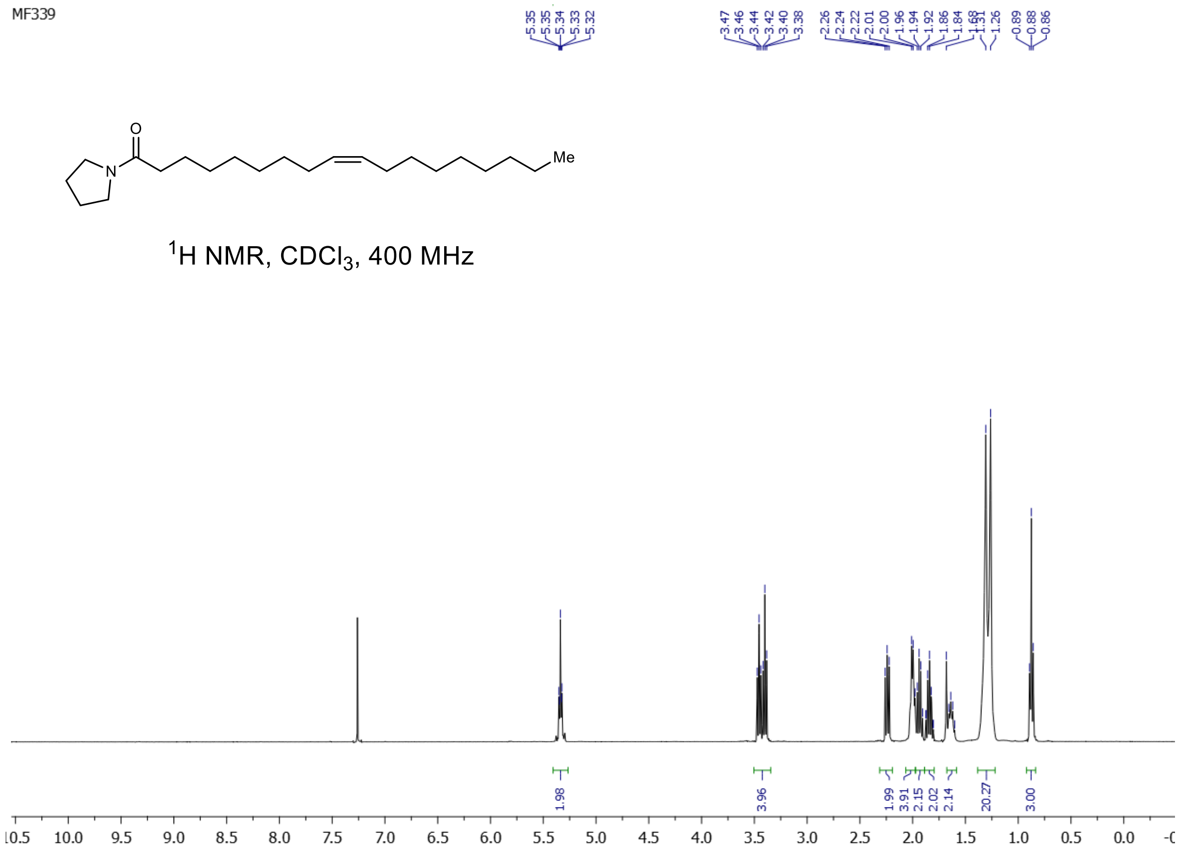

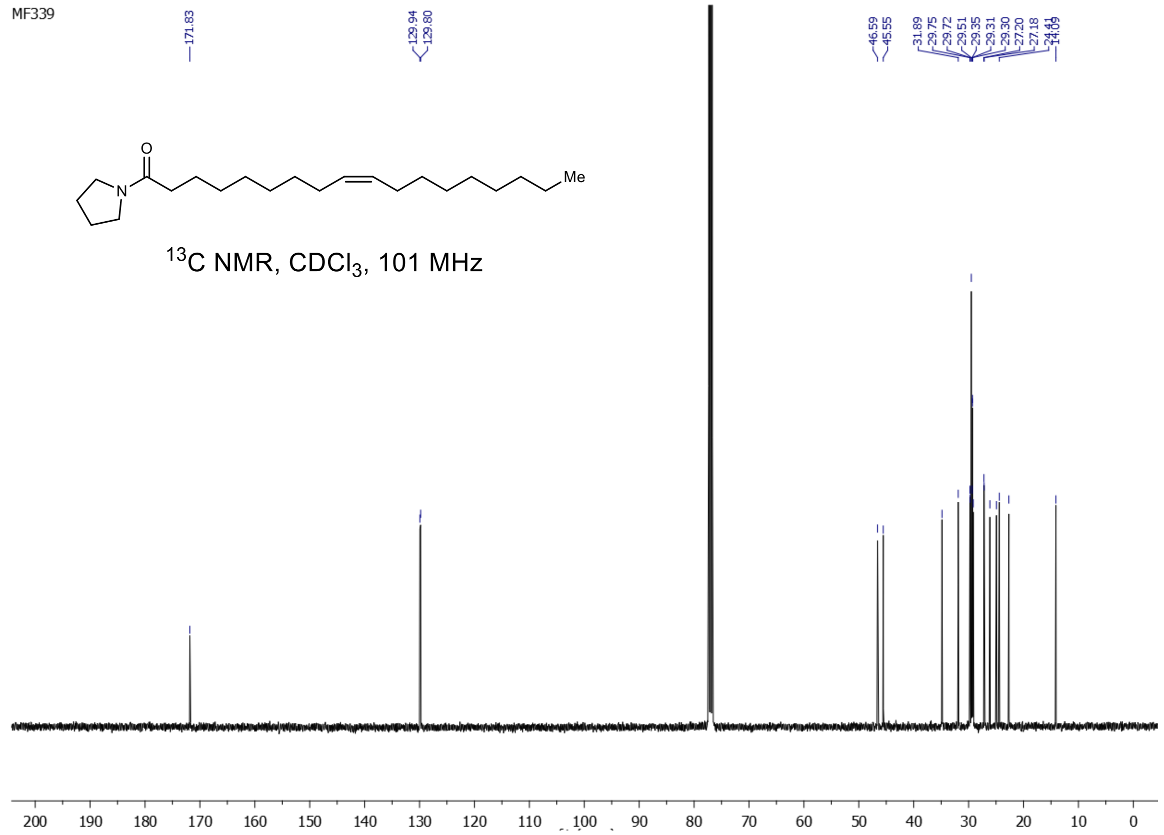

1-(Indolin-1-yl)hex-5-yn-1-one (2k)

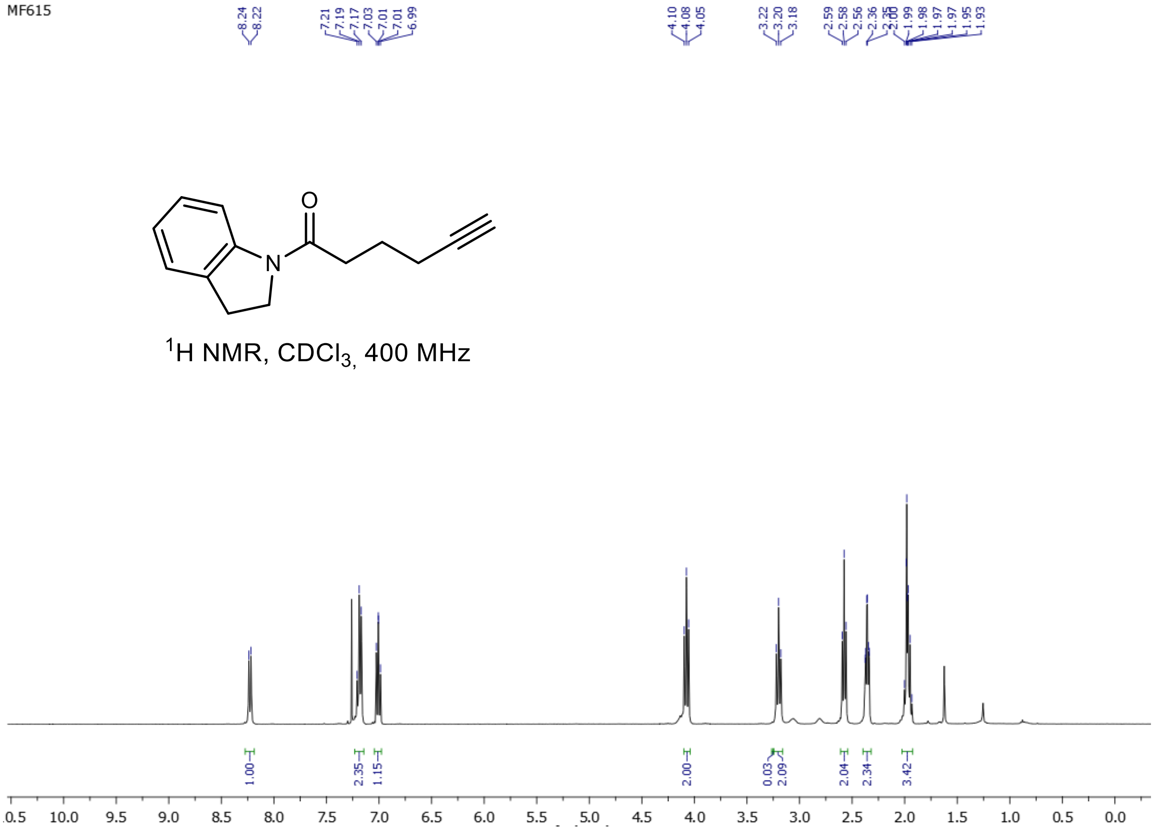

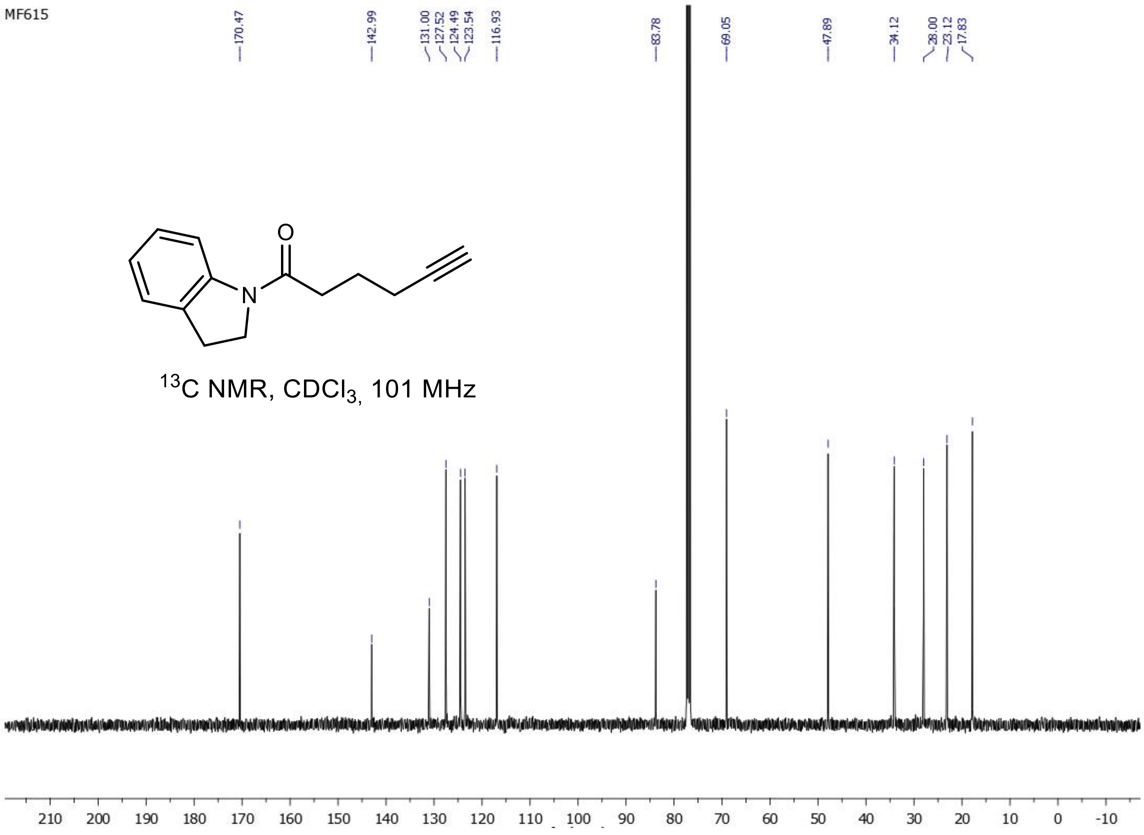

_N,N_-Dimethyl-10-oxoundecanamide (2m)

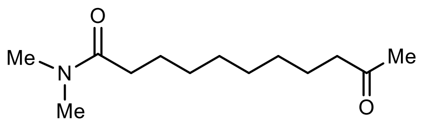

^{1}H NMR, $CDCl_3$, 400 MHz

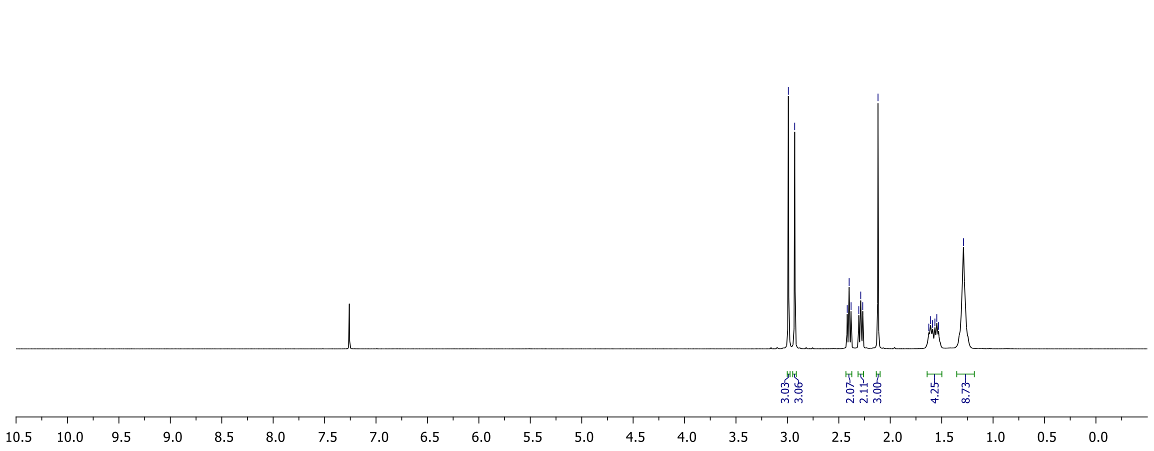

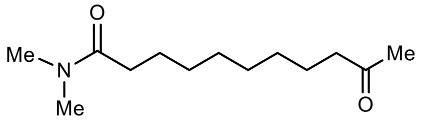

^{13}C NMR, $CDCl_3$, 101 MHz

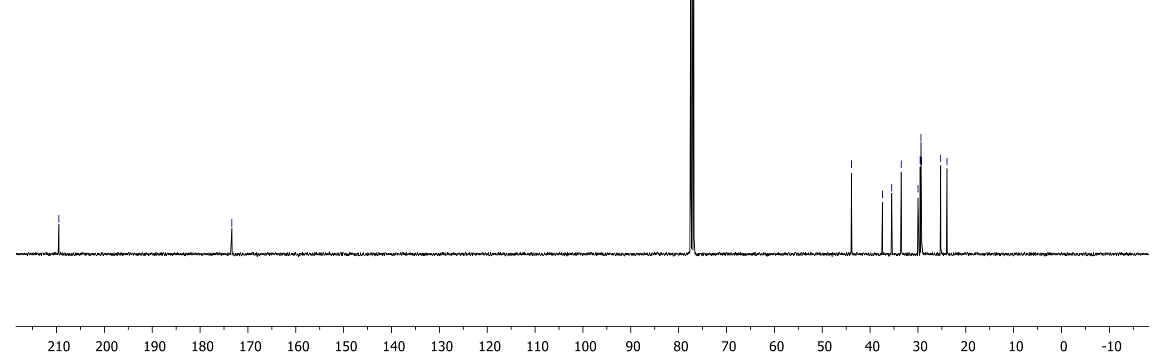

6-Cyano-*N,N*-dimethylhexanamide (2o)

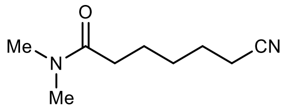

^{1}H NMR, $CDCl_3$, 600 MHz

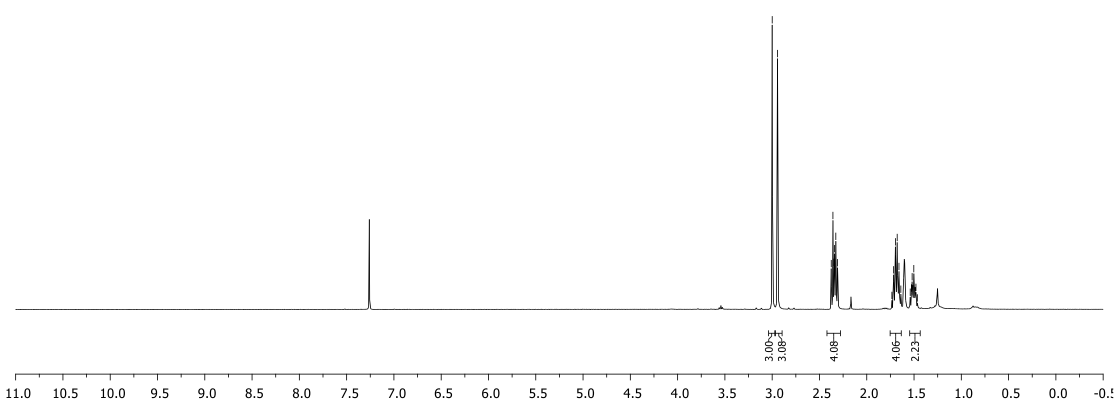

61Oct1422
Auftraggeber Maulide
JABR6

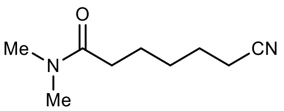

^{13}C NMR, $CDCl_3$, 151 MHz

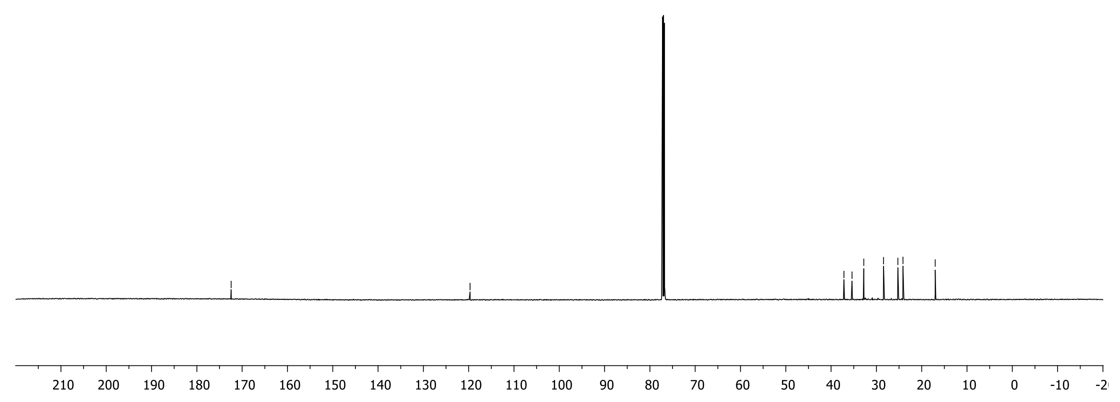

6-(1,3-Dioxoisoindolin-2-yl)-*N,N*-dimethylhexanamide (2p)

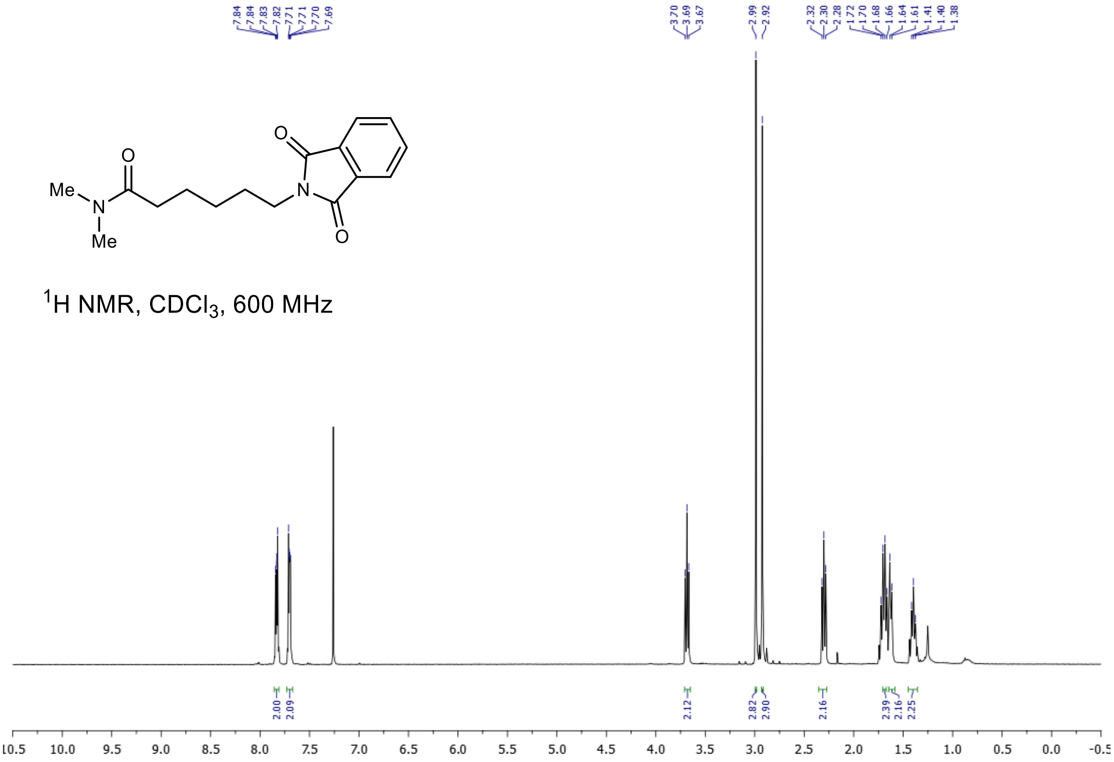

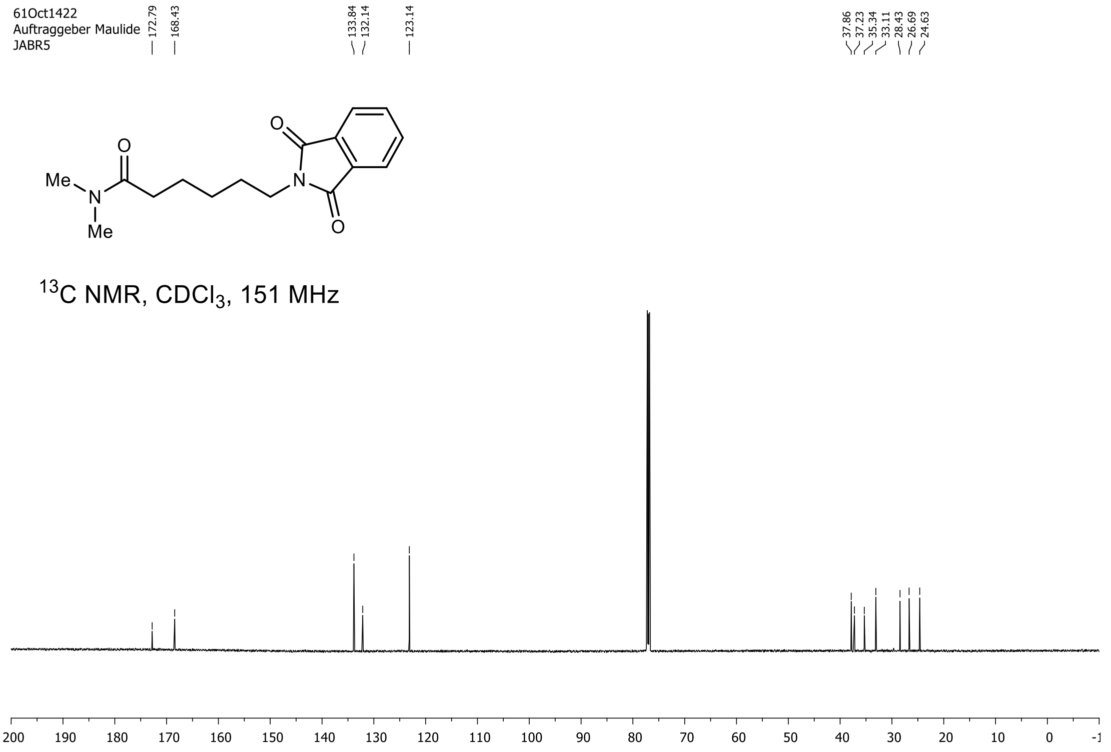

3-(Benzo[*b*]thiophen-2-yl)-*N,N*-dimethylpropanamide (2r)

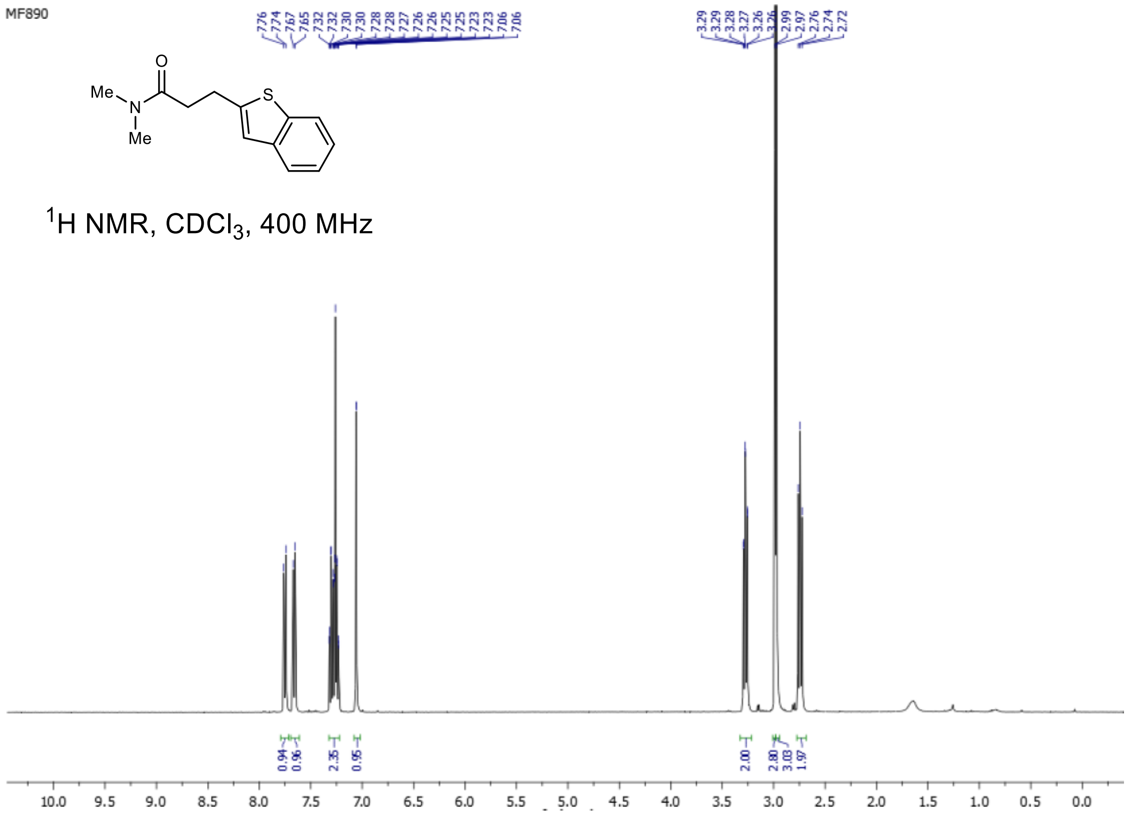

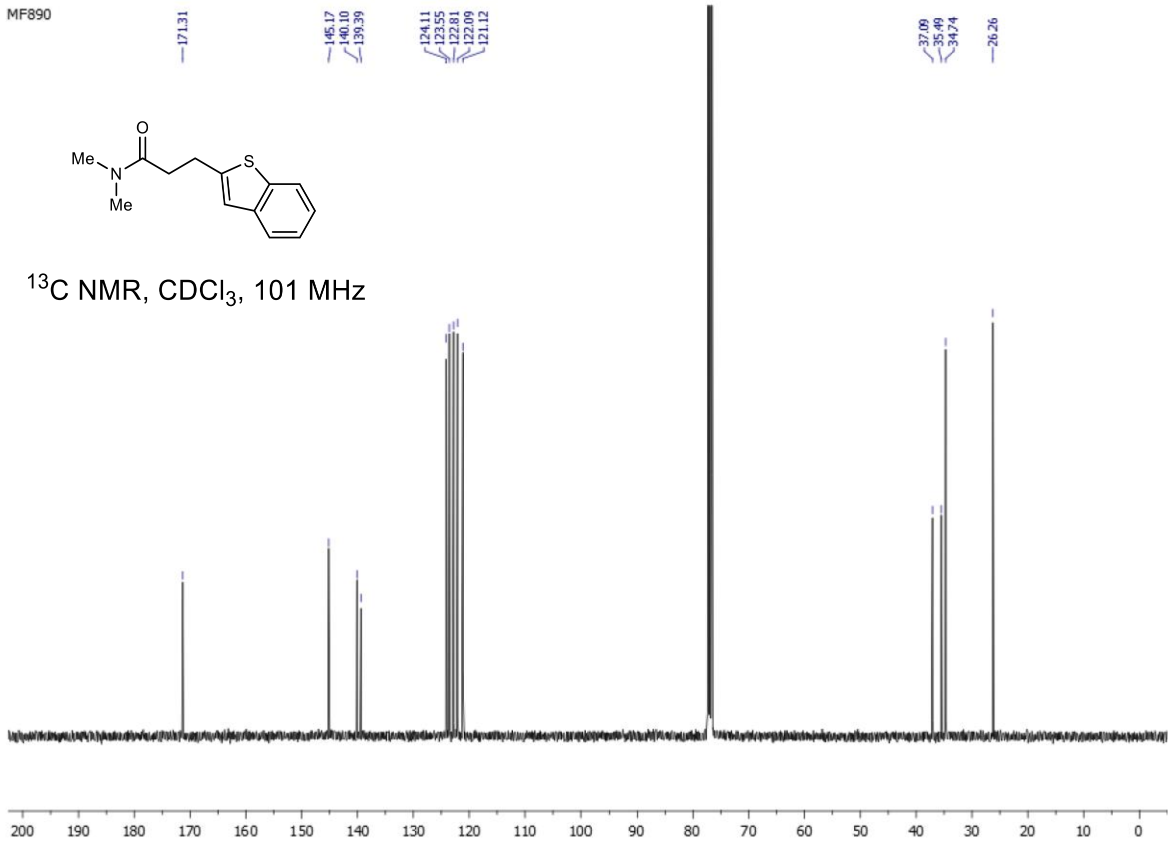

1-(5-Methoxyindolin-1-yl)-3-phenylpropan-1-one (2s)

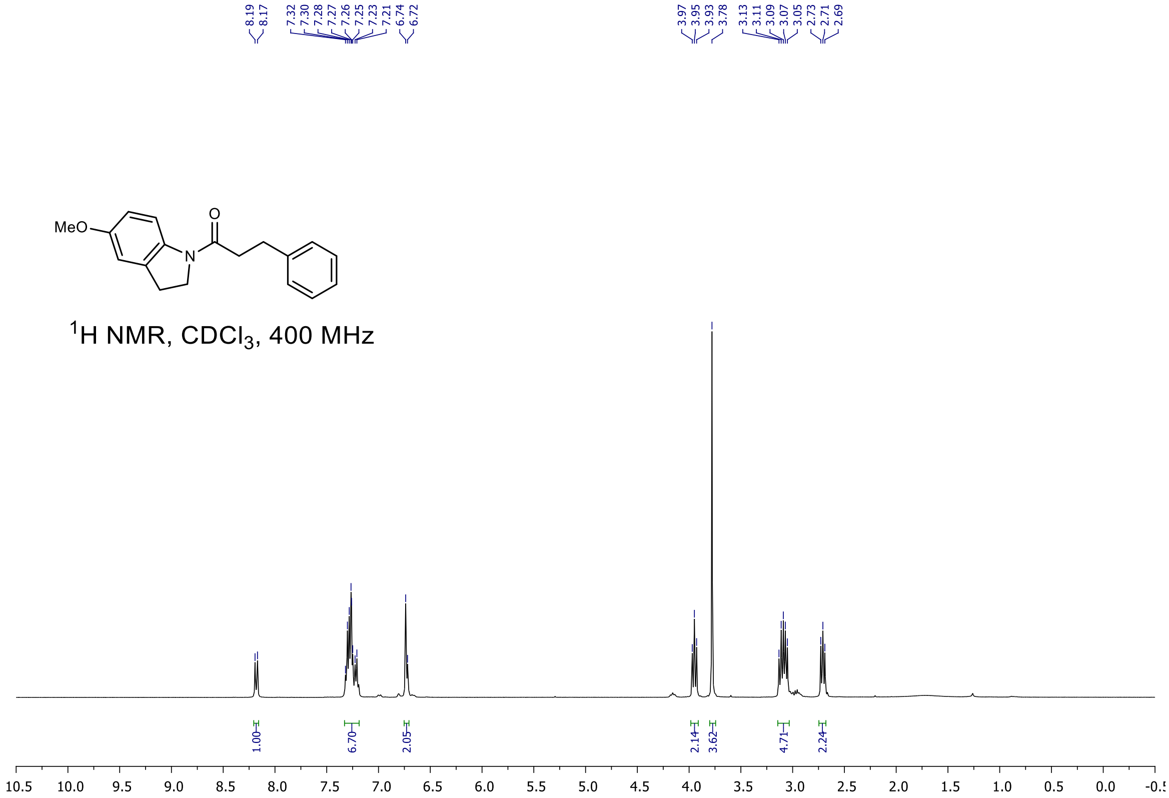

^{1}H NMR, $CDCl_3$, 400 MHz

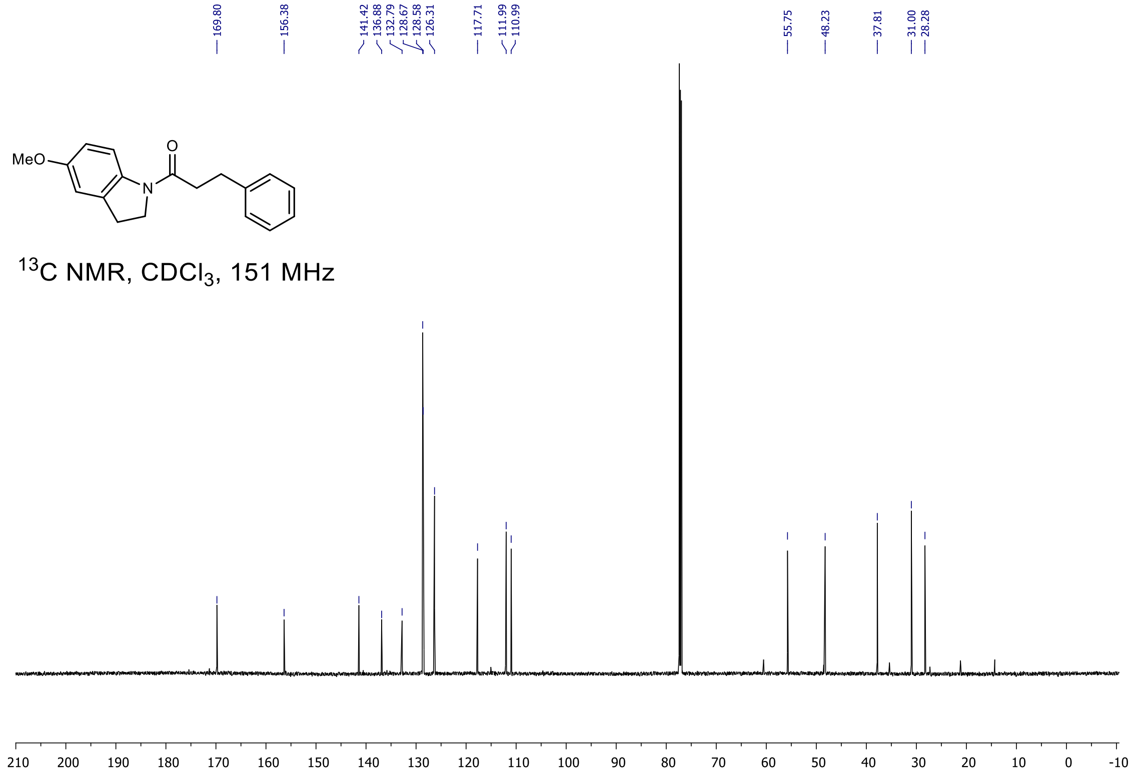

^{13}C NMR, $CDCl_3$, 151 MHz

1-(Indolin-1-yl)-3-(4-(trifluoromethyl)phenyl)propan-1-one (2t)

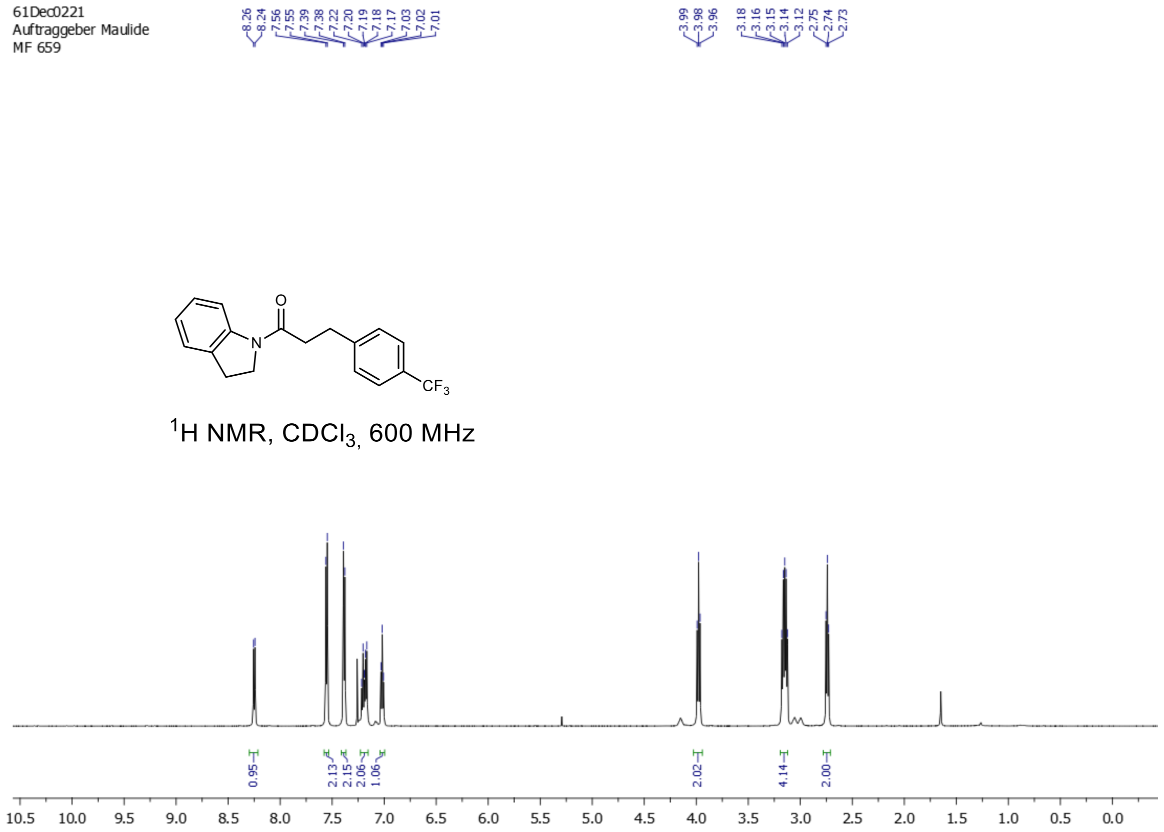

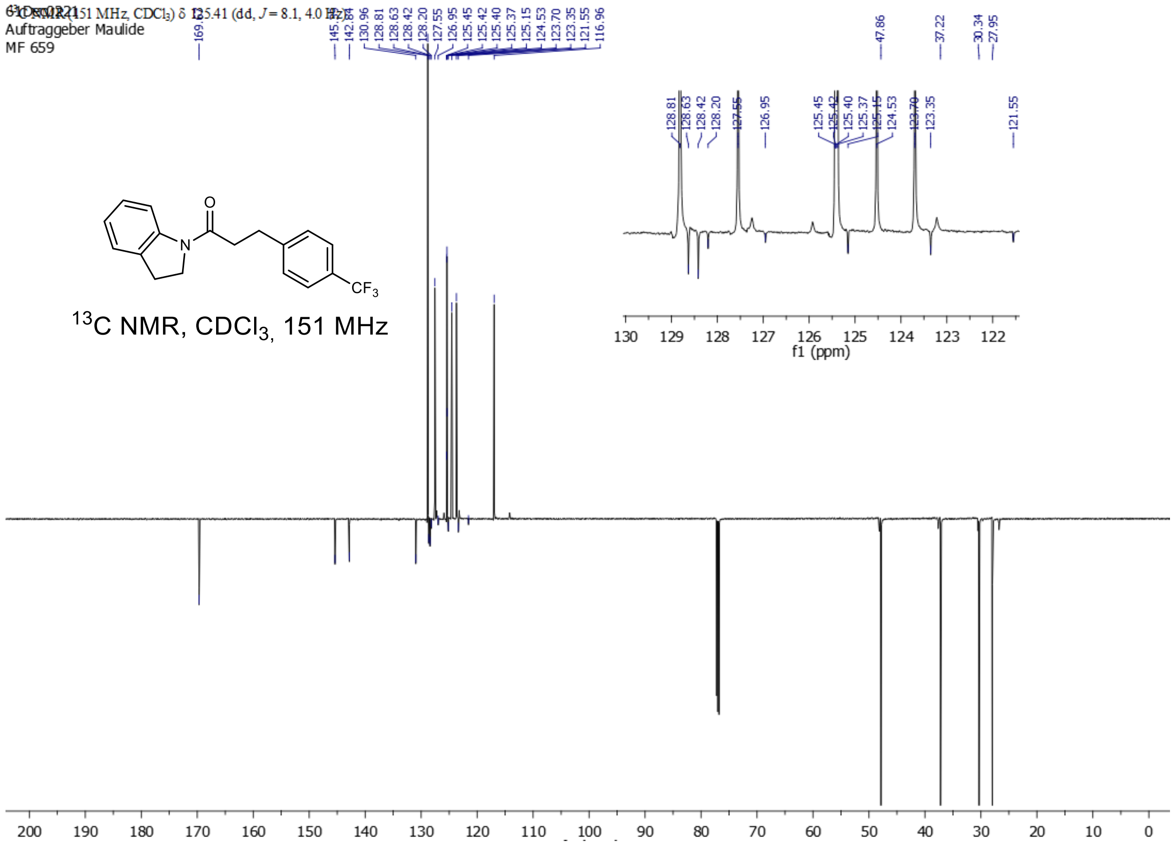

61Dec0221
Auftraggeber Maulide
MF 659

-62.35

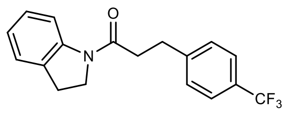

^{19}F NMR, $CDCl_3$, 565 MHz

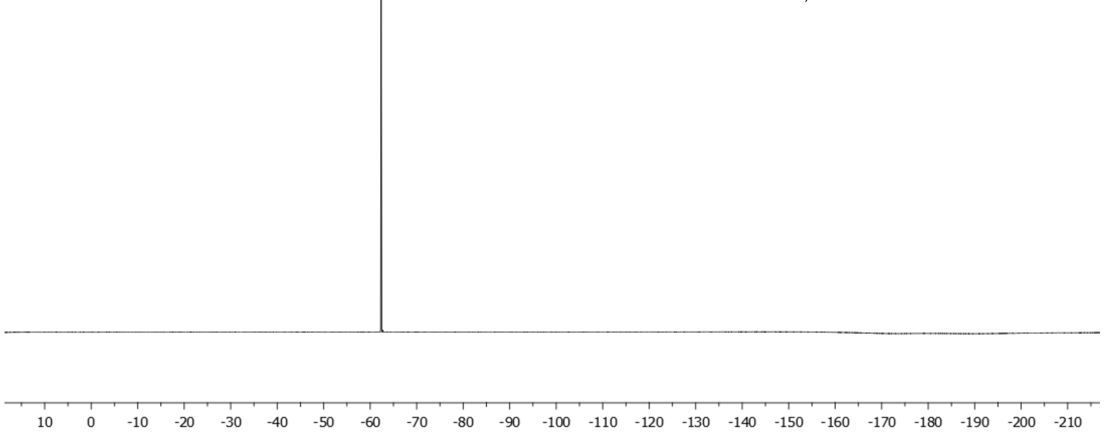

1-(Indolin-1-yl)heptane-1,6-dione (2w)

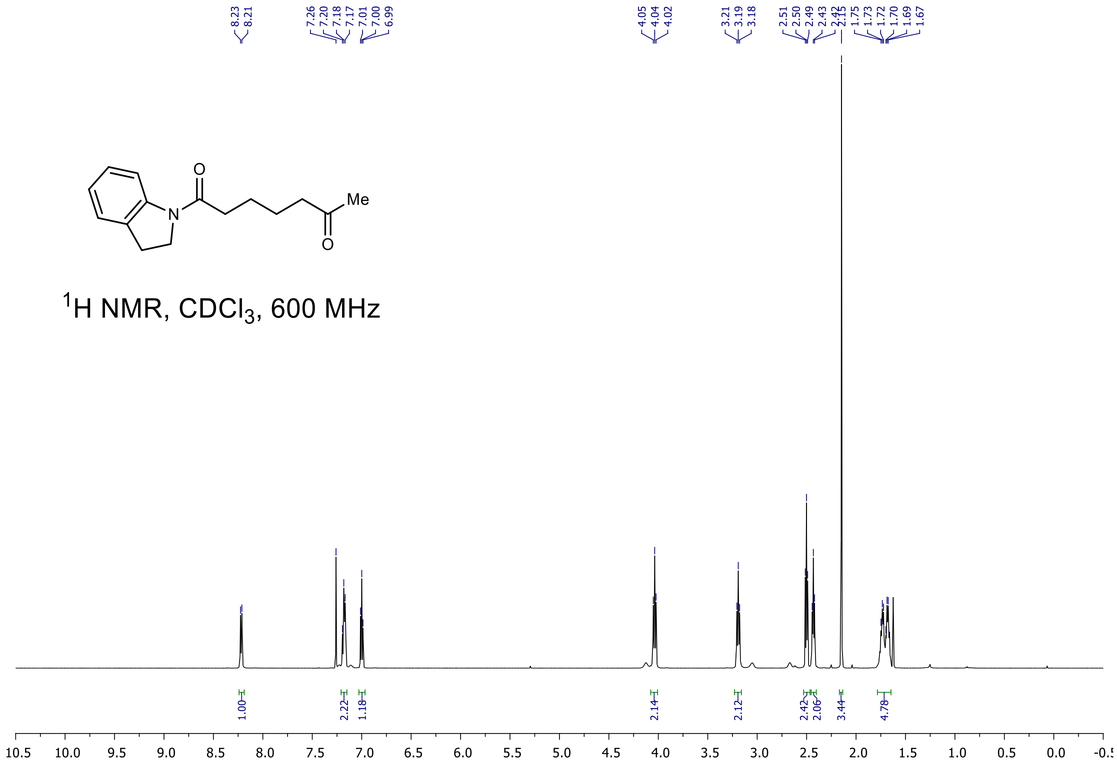

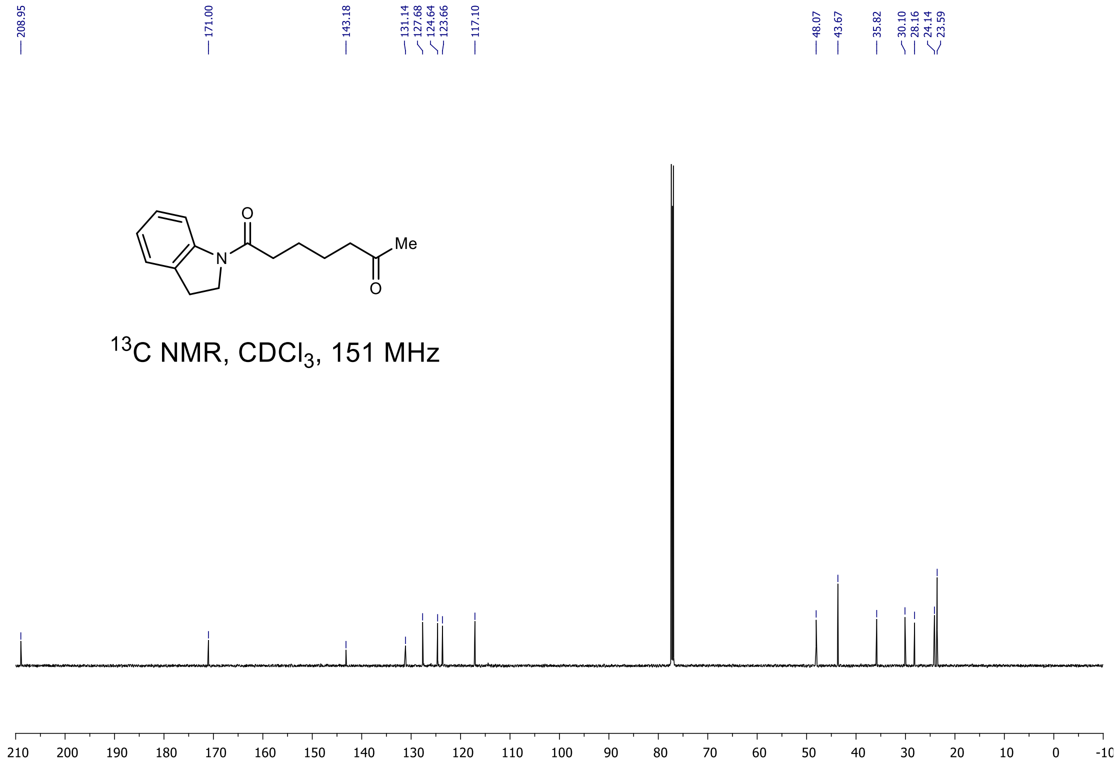

2-Amino-*N,N*-dimethyl-4-phenylbutanamide (4a)

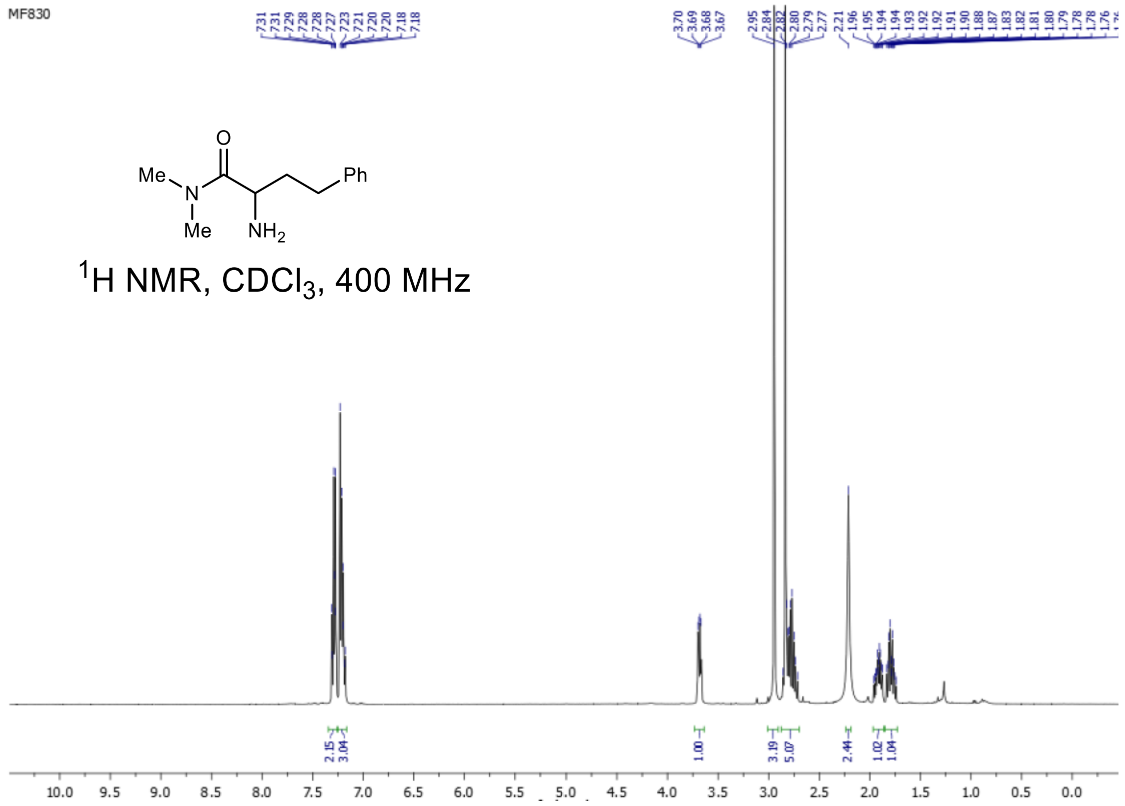

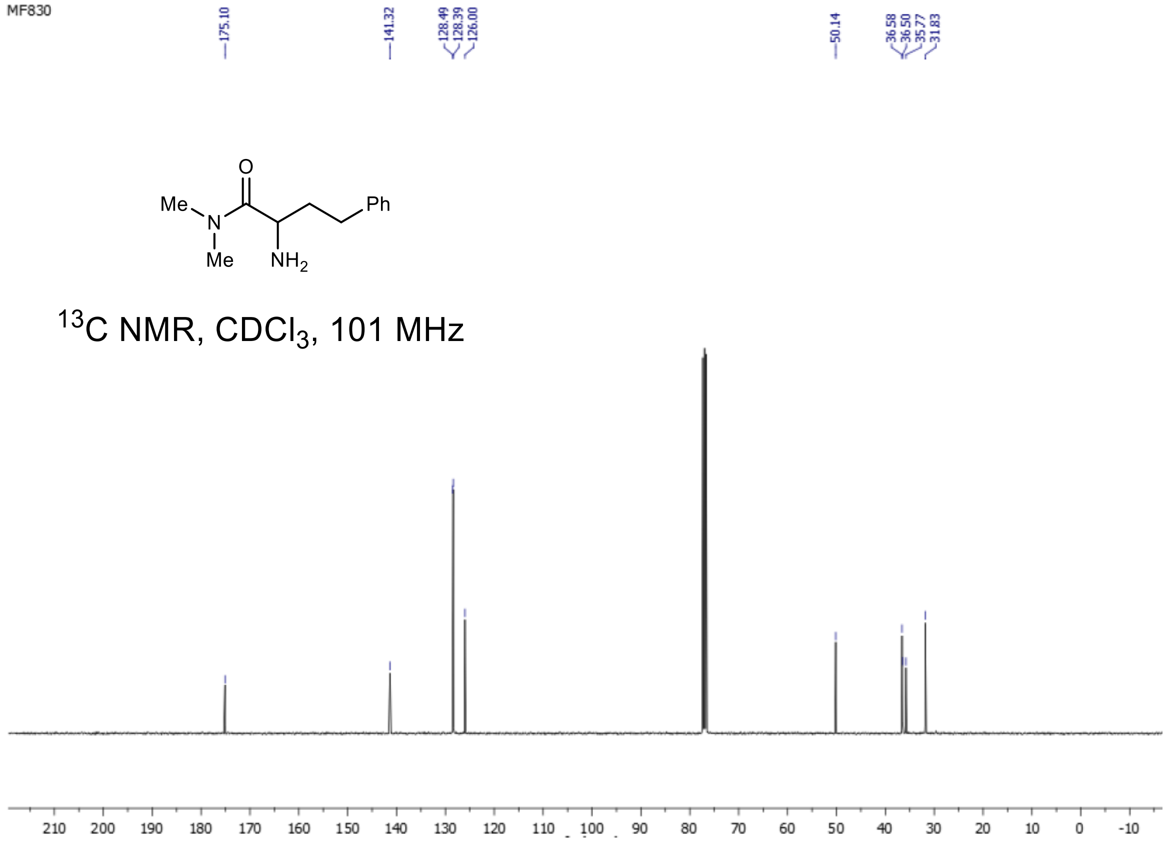

2-Amino-3-phenyl-1-(pyrrolidin-1-yl)propan-1-one (4b)

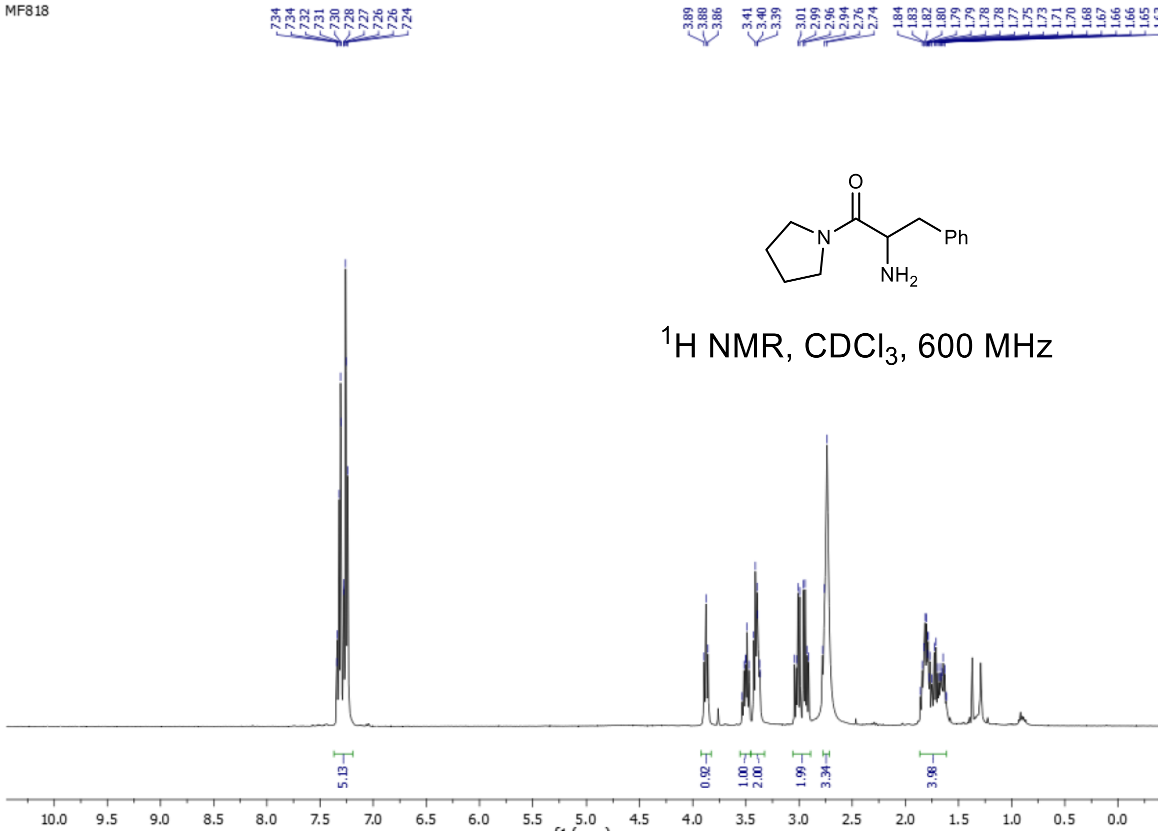

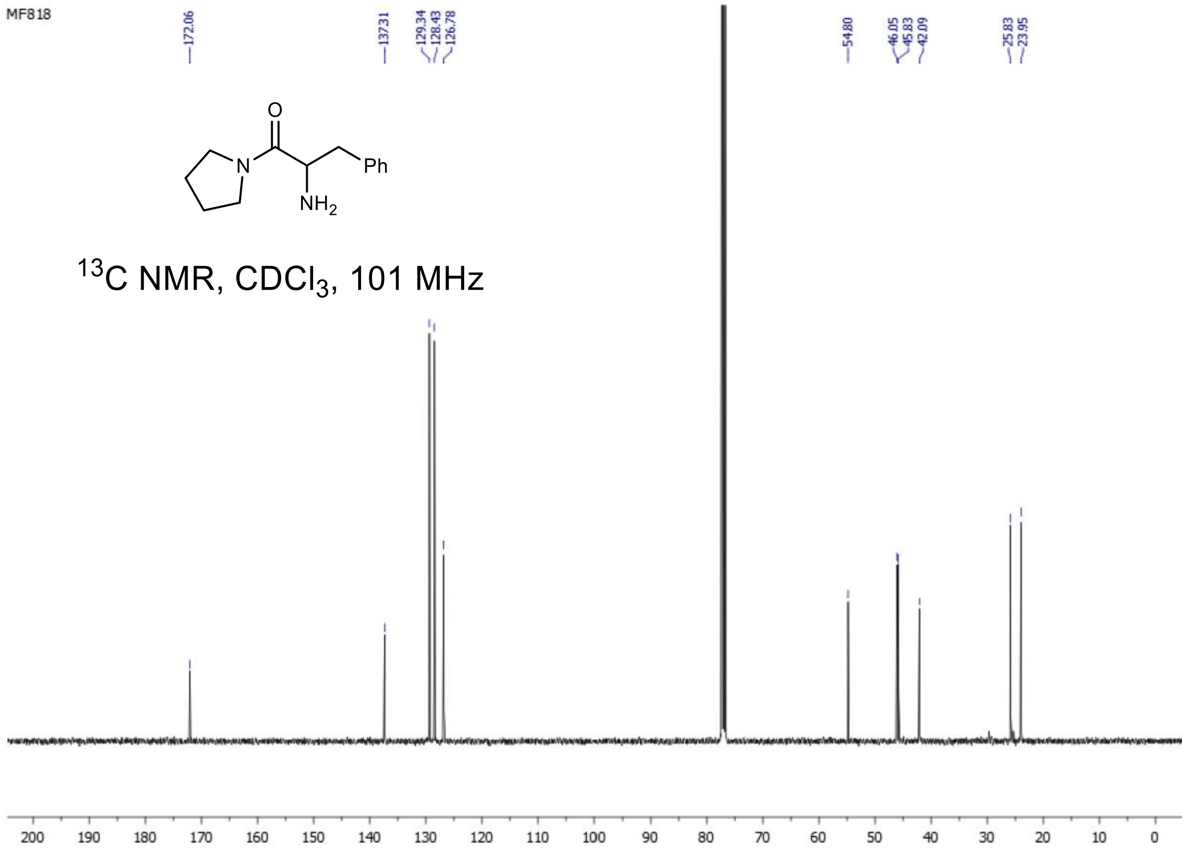

2-Amino-3-phenyl-1-(piperidin-1-yl)propan-1-one (4c)

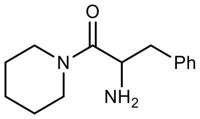

^{1}H NMR, $CDCl_3$, 400 MHz

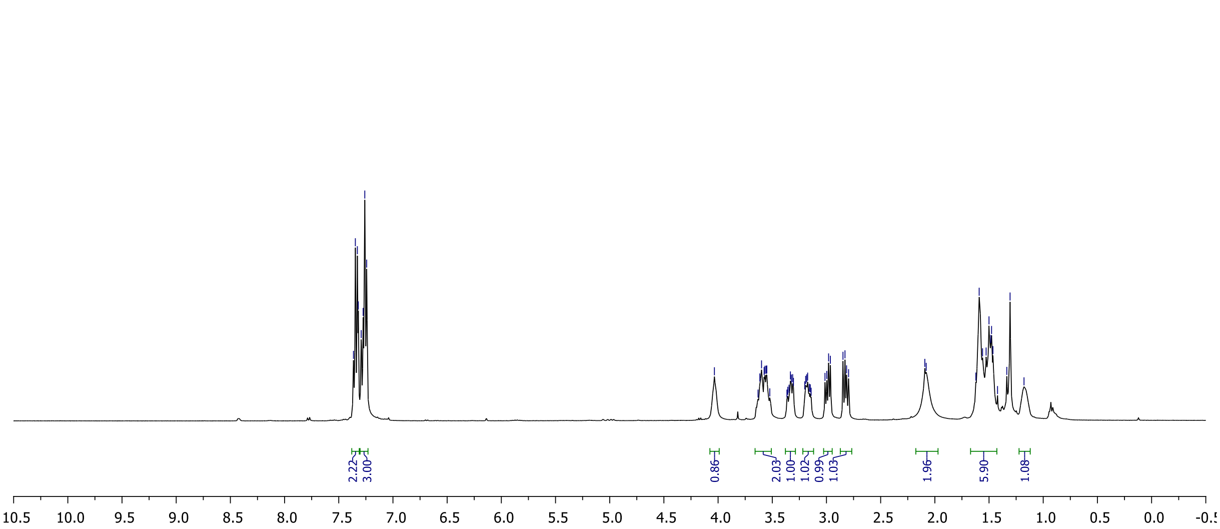

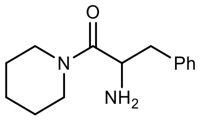

^{13}C NMR, $CDCl_3$, 151 MHz

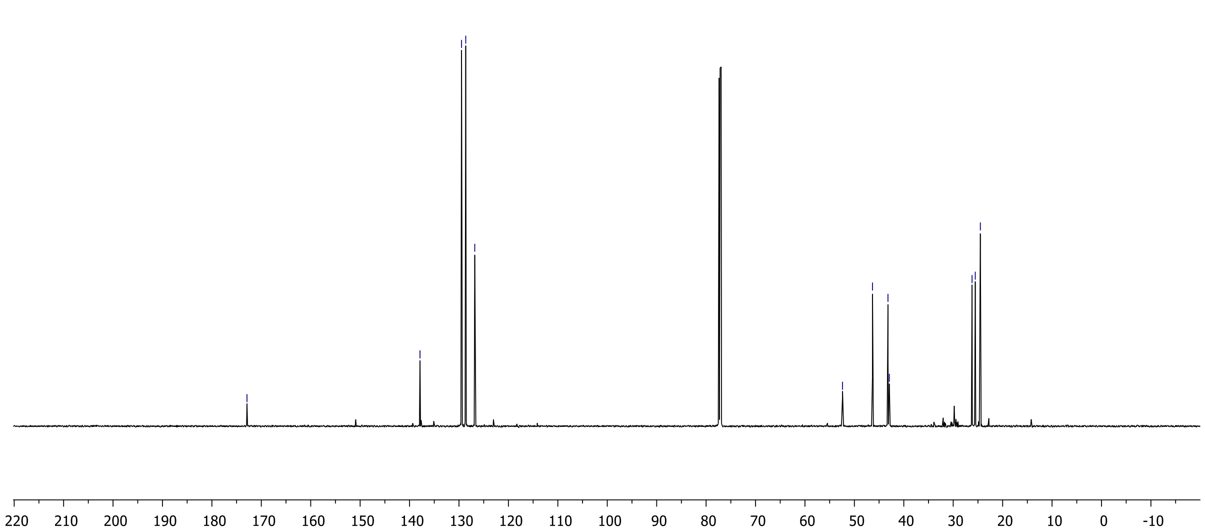

2-Amino-1-morpholino-4-phenylbutan-1-one (4d)

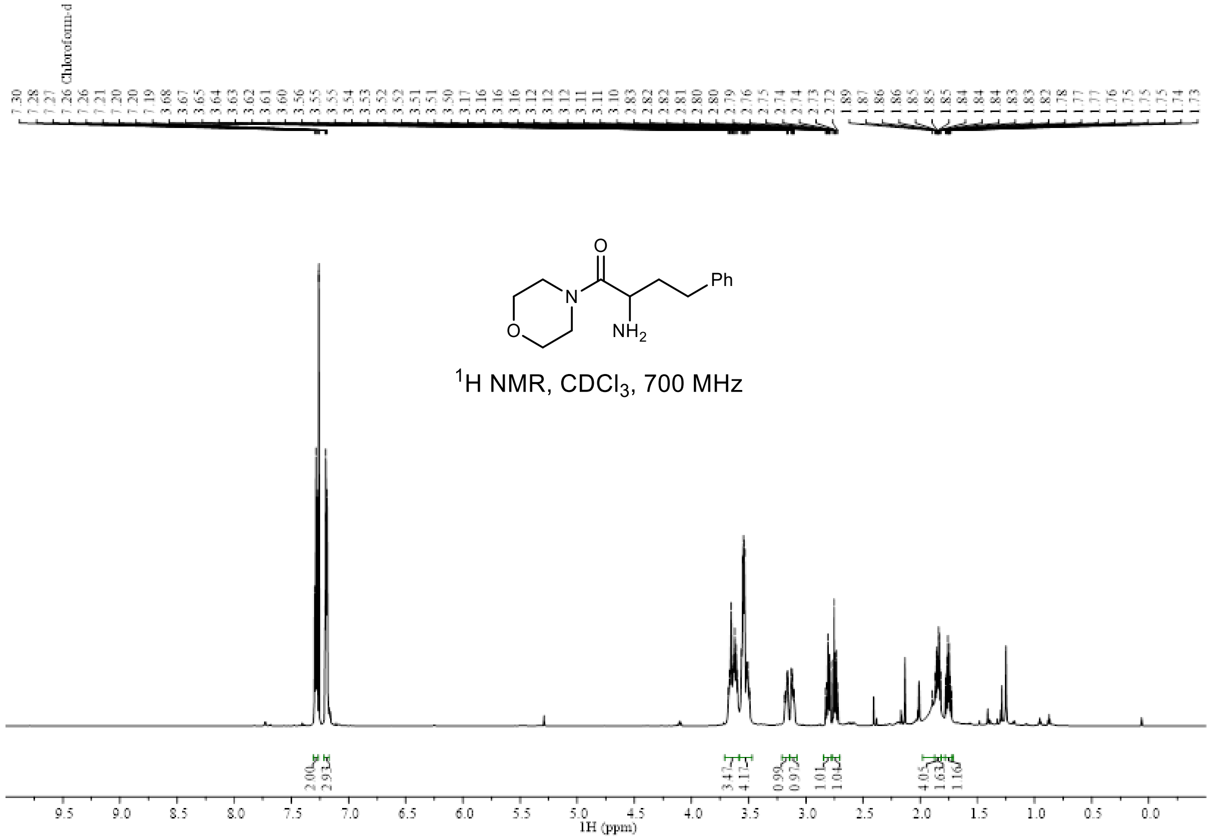

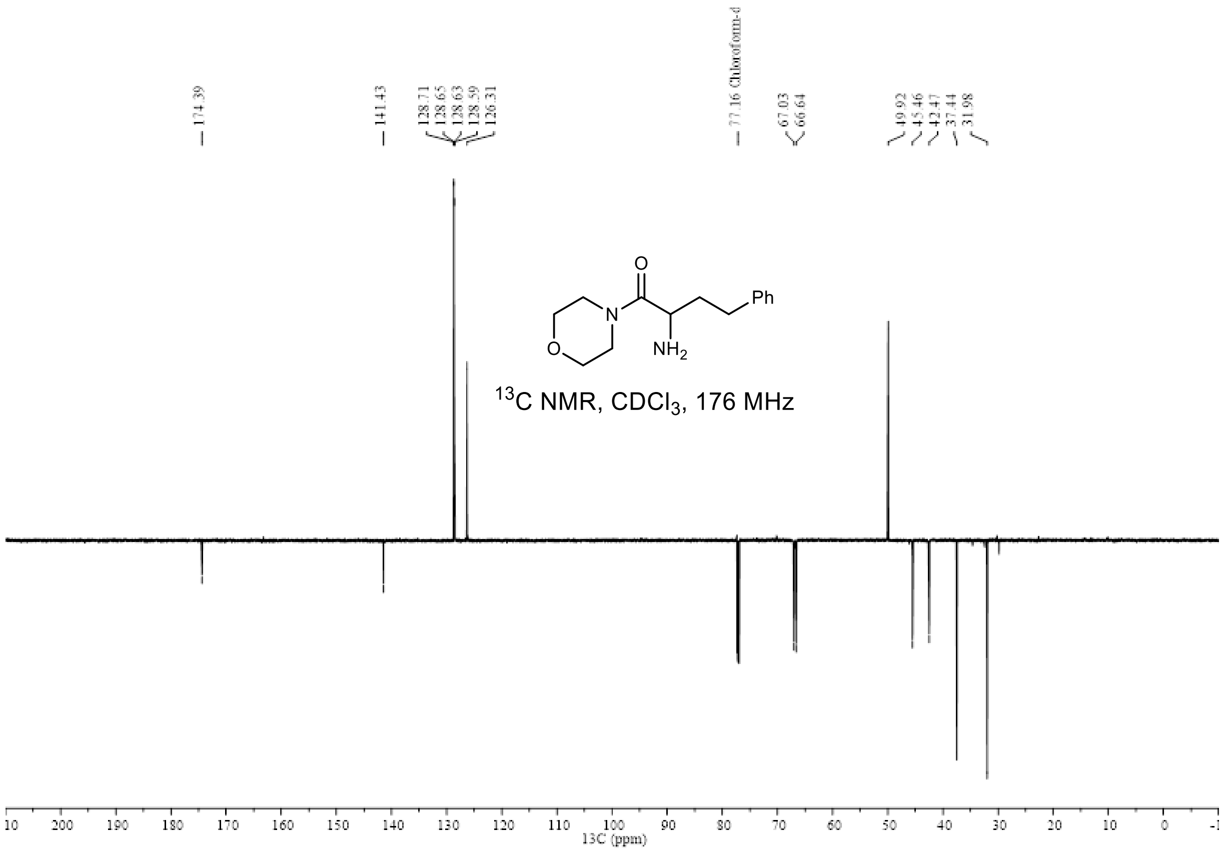

2-Amino-*N*-methoxy-*N*-methyl-4-phenylbutanamide (4e)

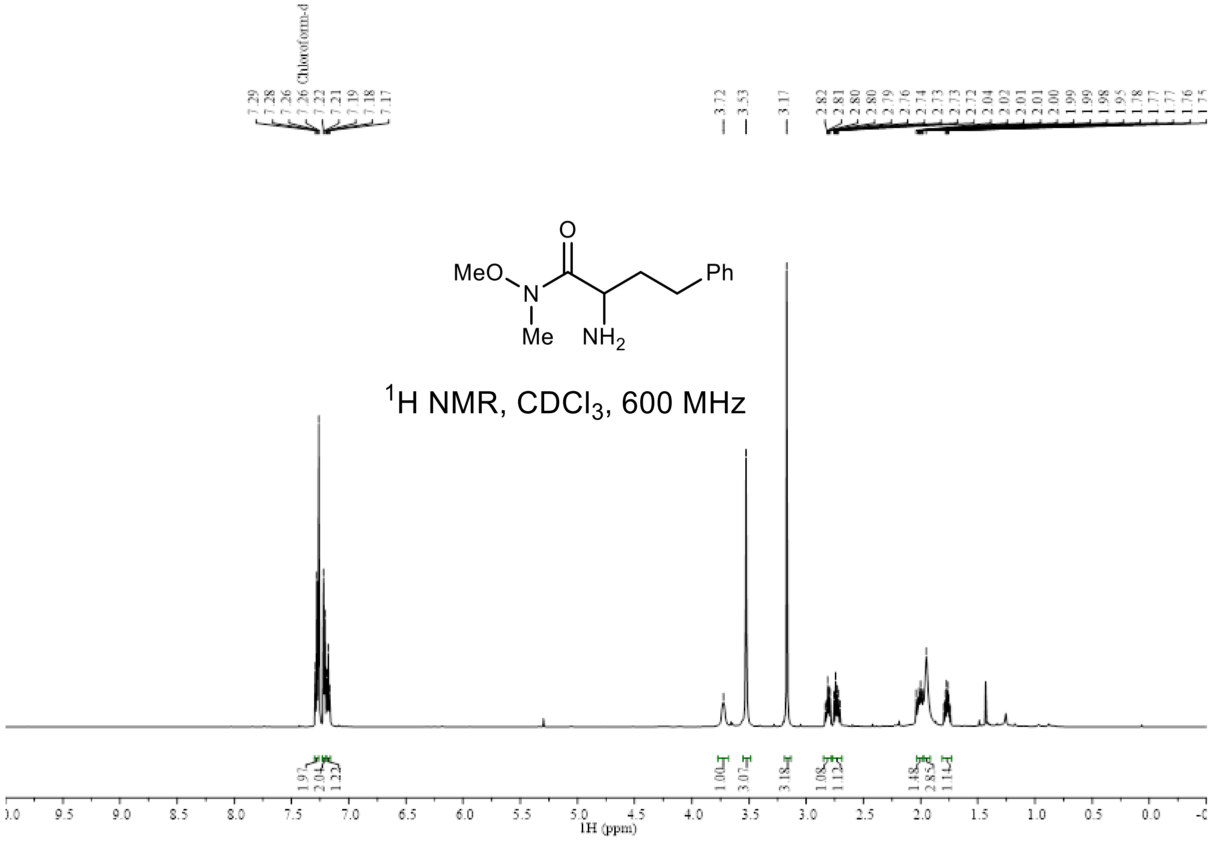

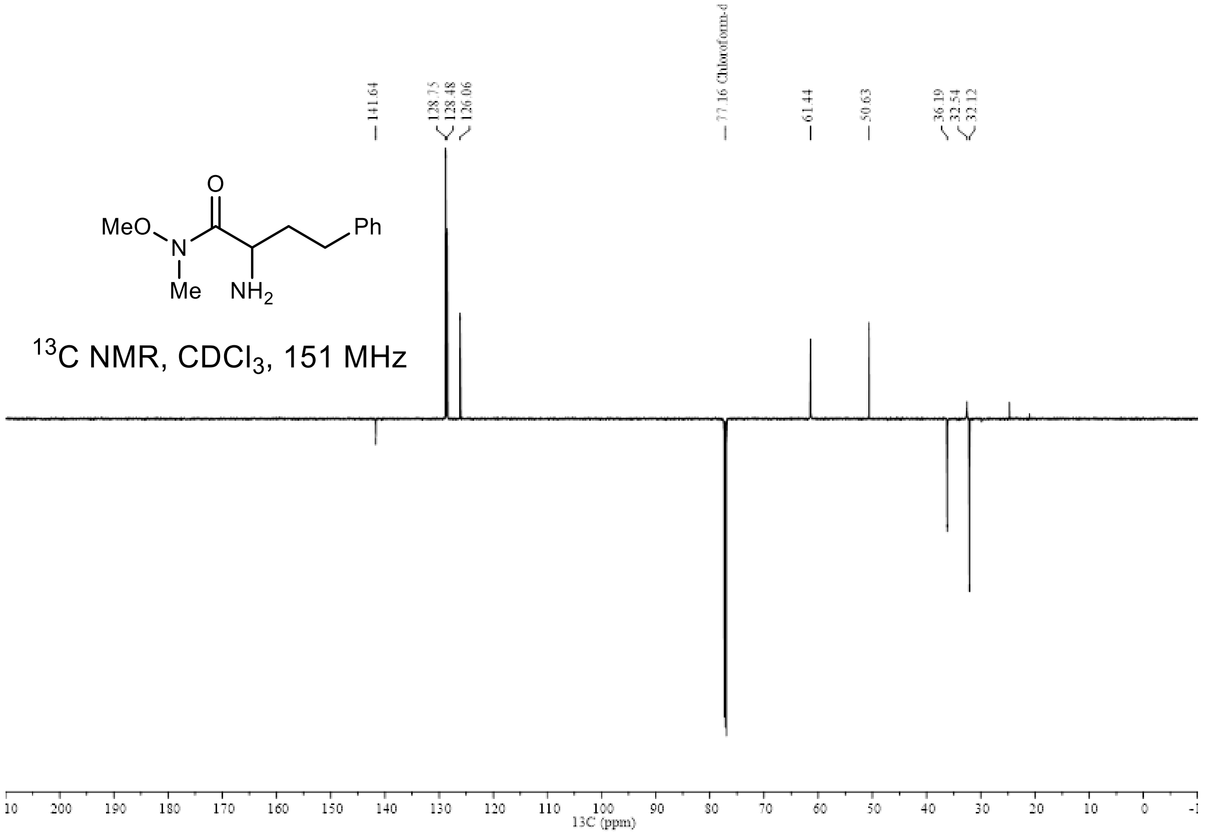

2-Amino-1-(indolin-1-yl)-3-(naphthalen-1-yl)propan-1-one (4f)

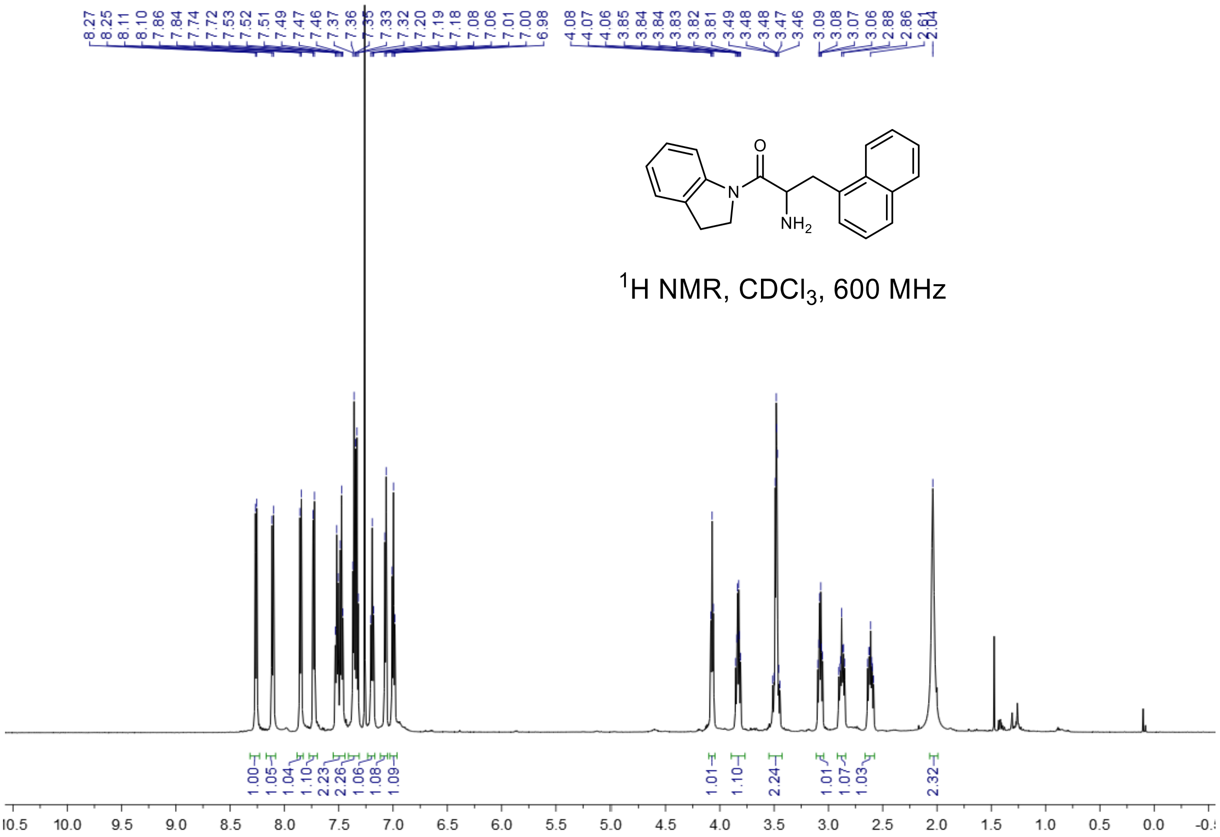

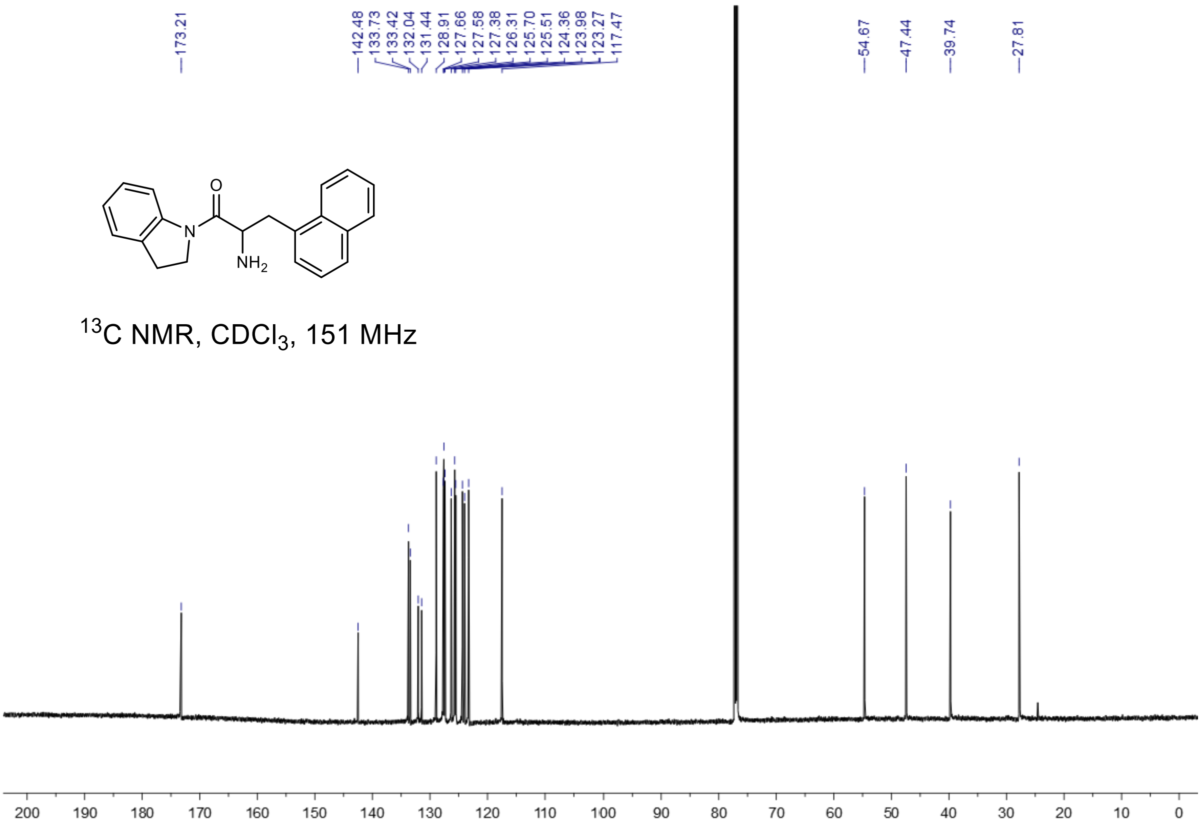

2-Amino-*N,N*-dimethylpentanamide (4g)

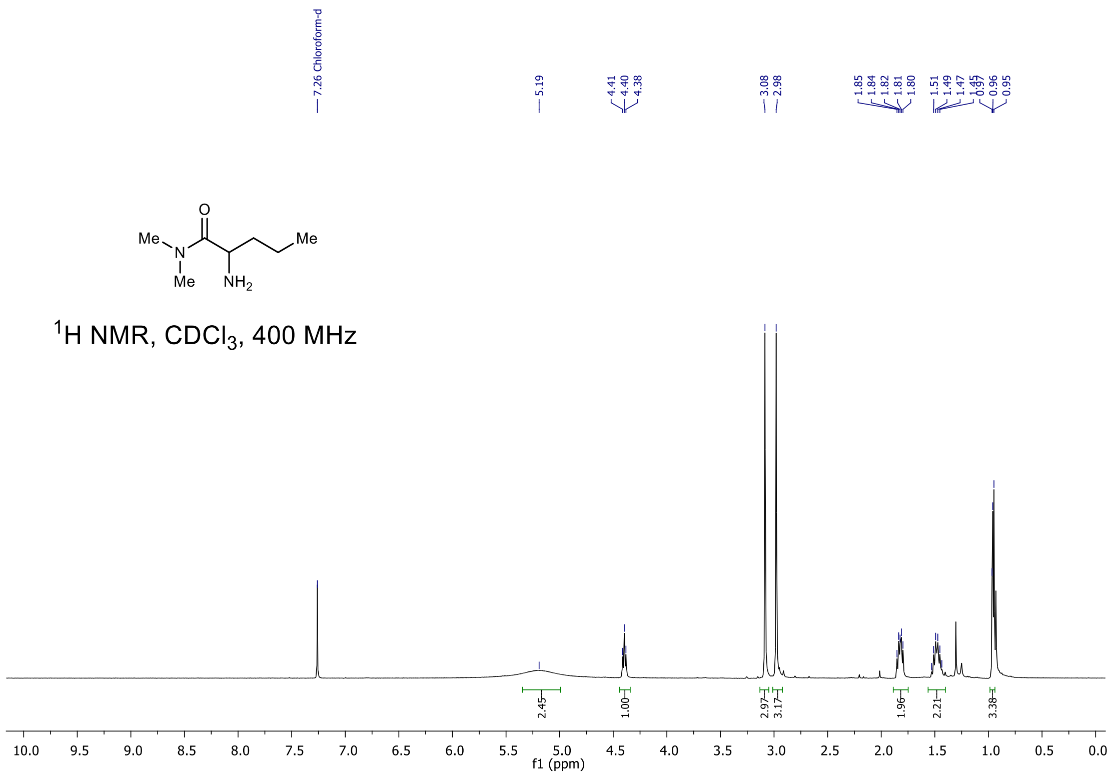

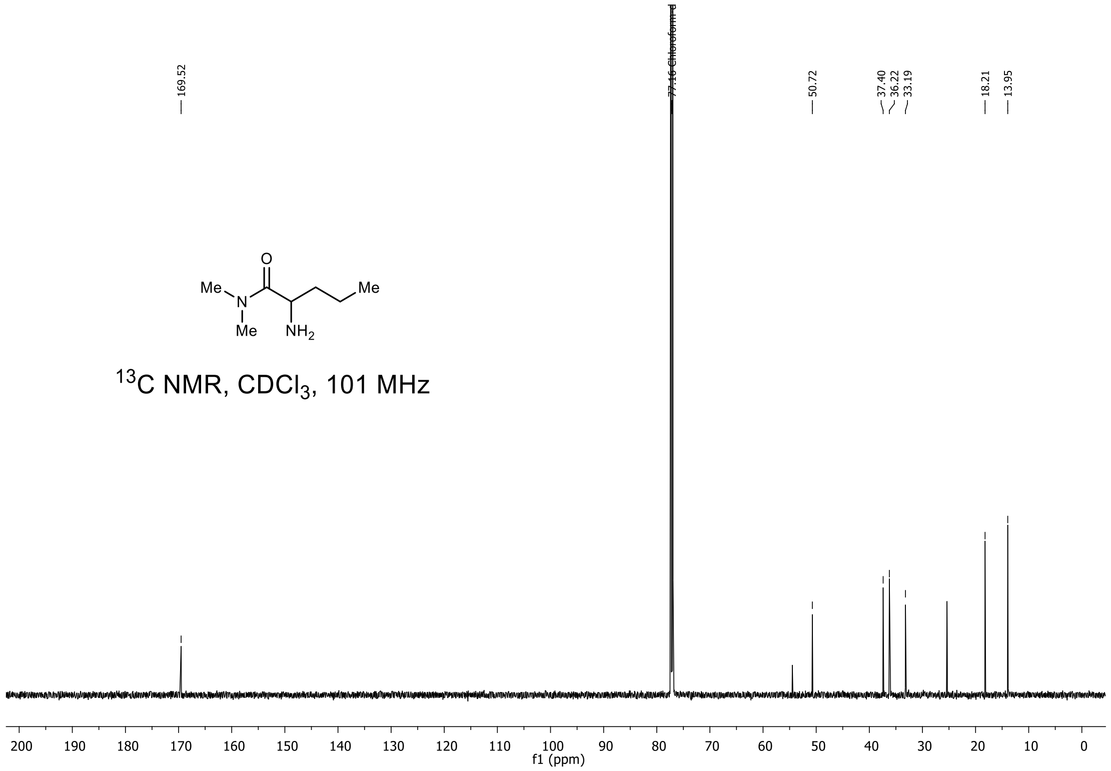

2-Amino-4,4,4-trifluoro-*N,N*-dimethylbutanamide (4h)

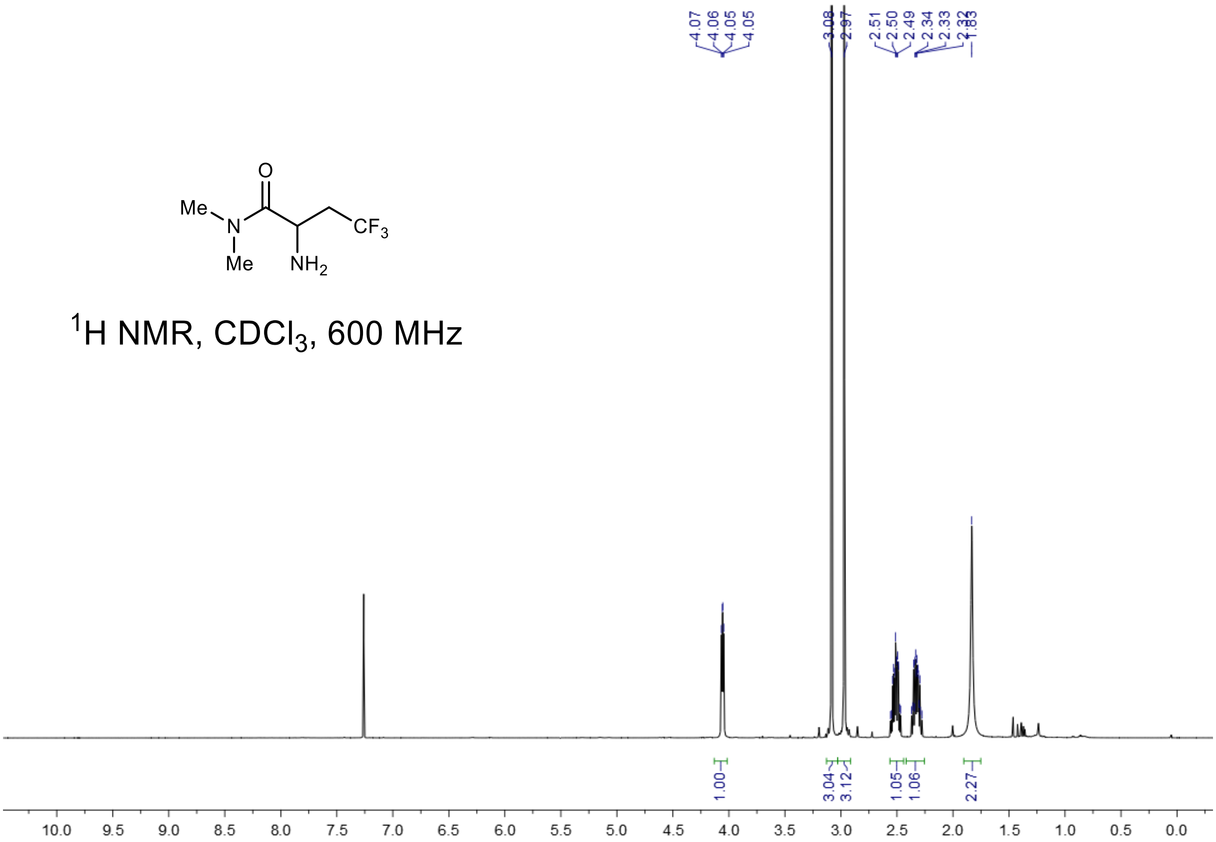

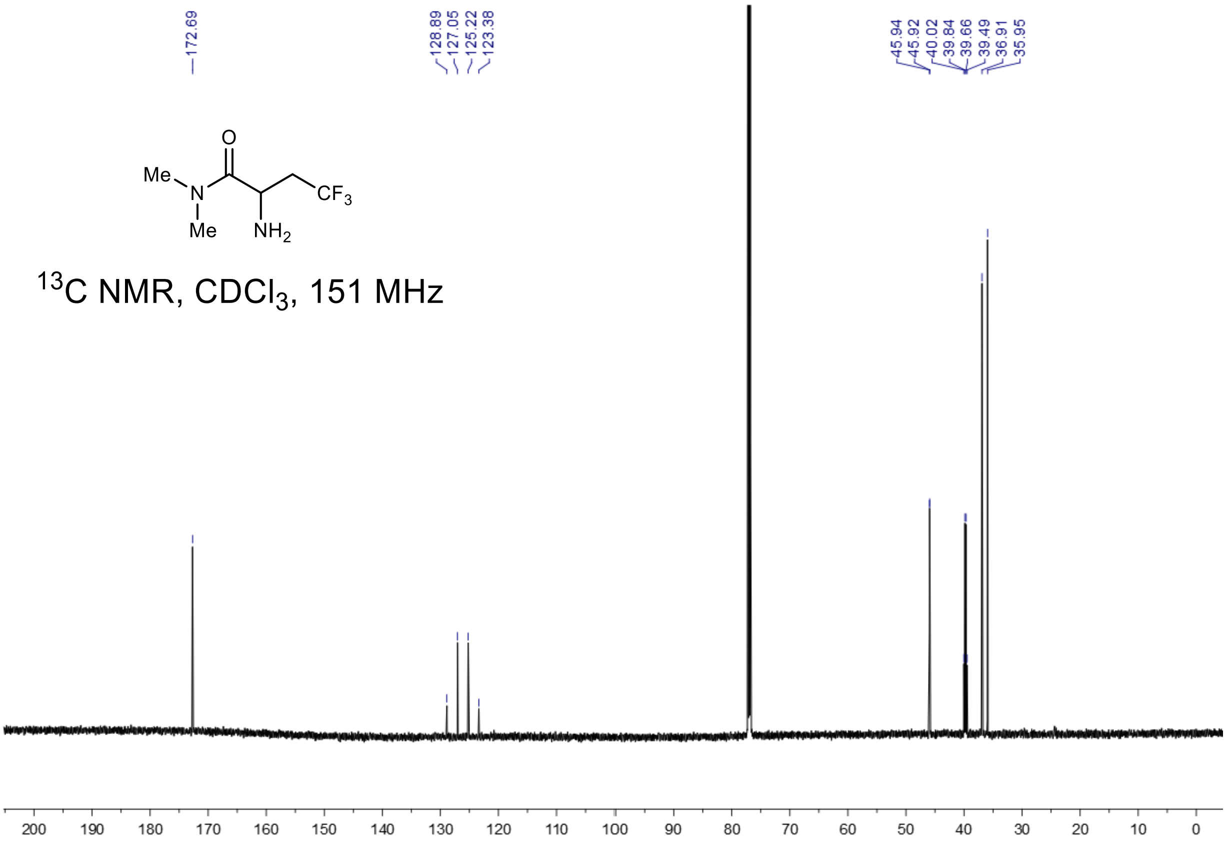

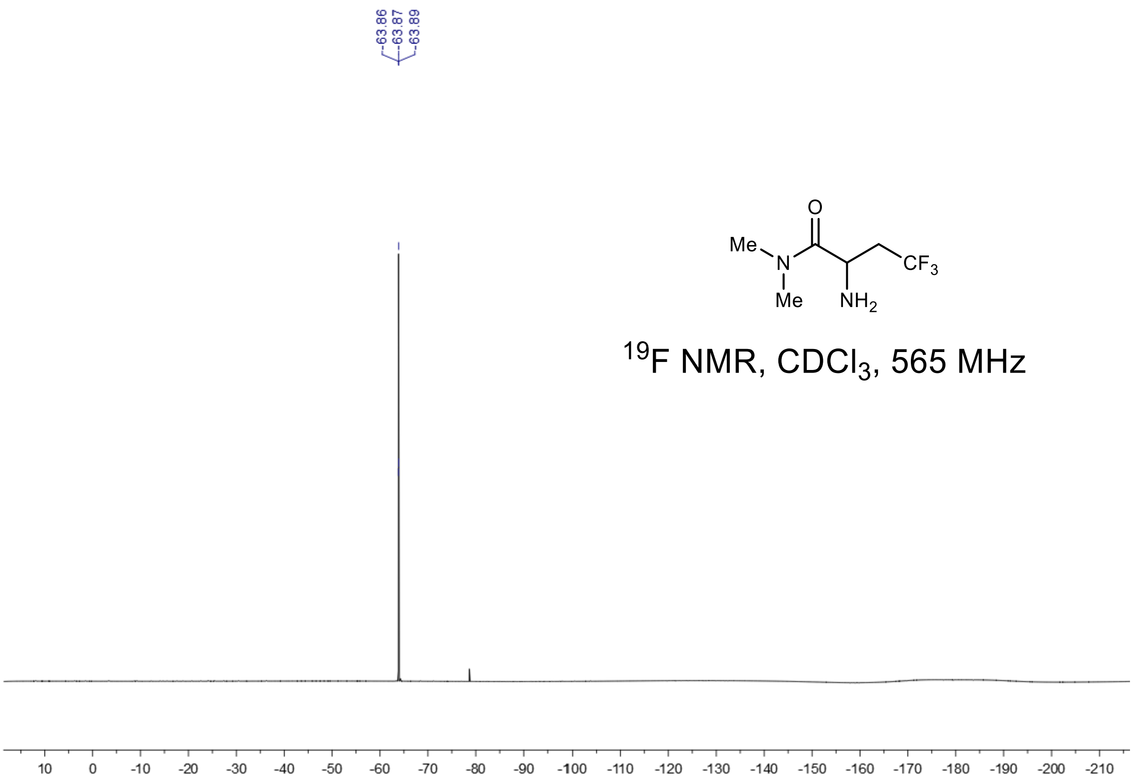
-63.86
-63.87
-63.89
O
Me
N
Me
NH2
CF3
19F NMR, CDCl3, 565 MHz
10 0 -10 -20 -30 -40 -50 -60 -70 -80 -90 -100 -110 -120 -130 -140 -150 -160 -170 -180 -190 -200 -210

2-Amino-*N,N*-dimethylundec-10-enamide (4i)

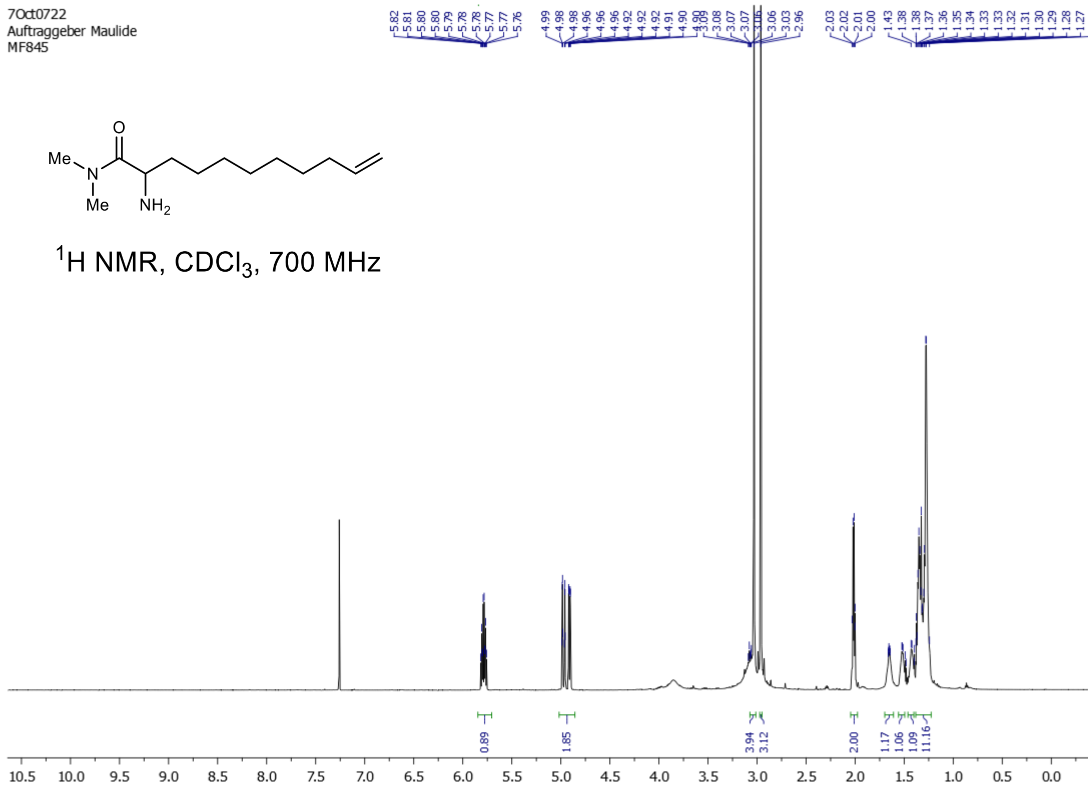

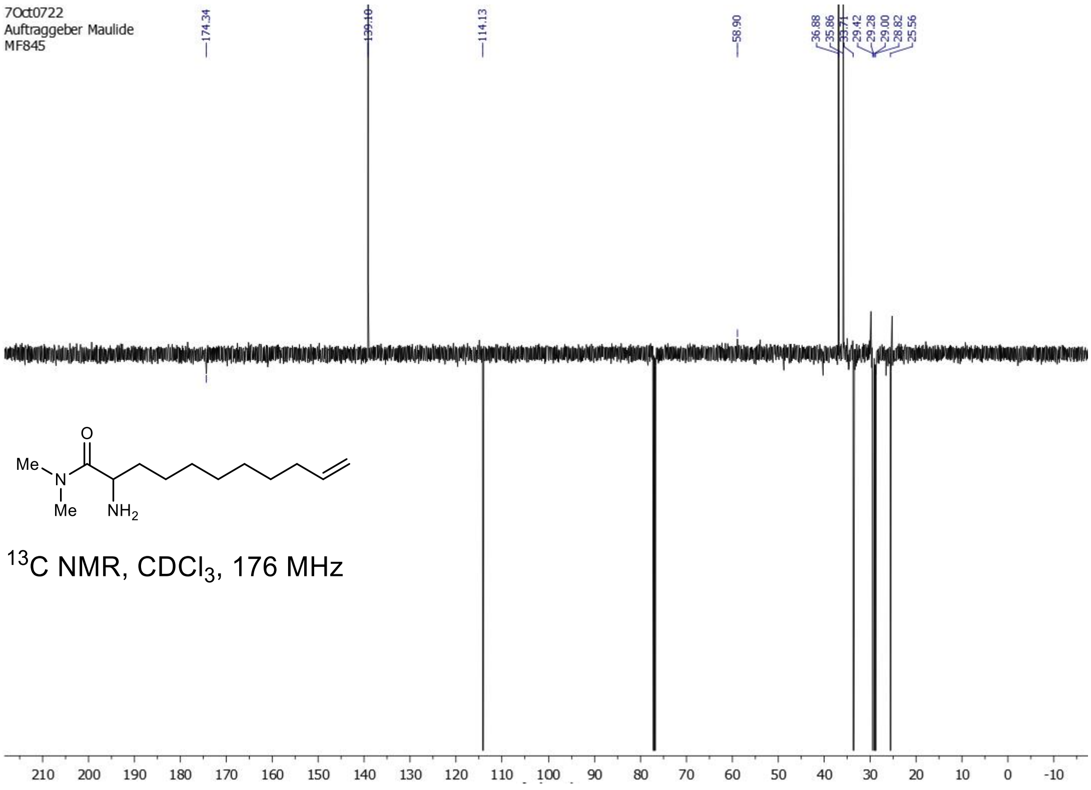

(*Z*)-2-Amino-1-(Pyrrolidin-1-yl)octadec-9-en-1-one (4j)

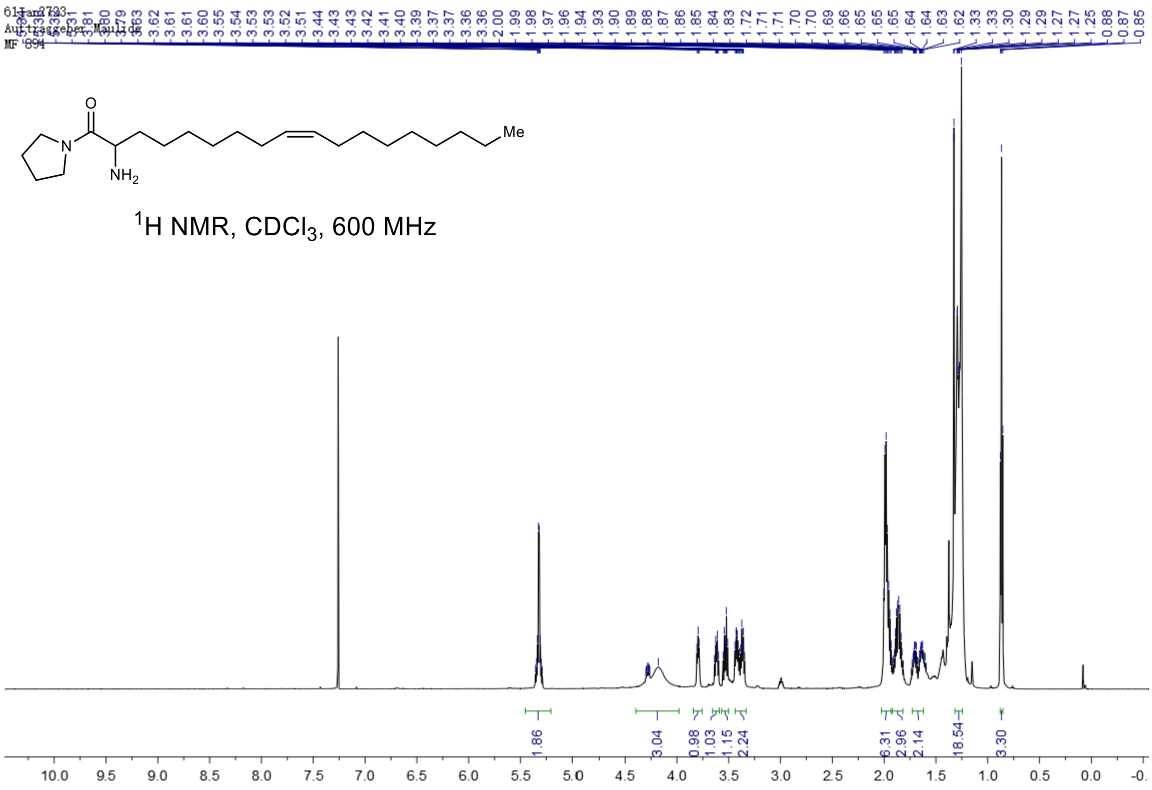

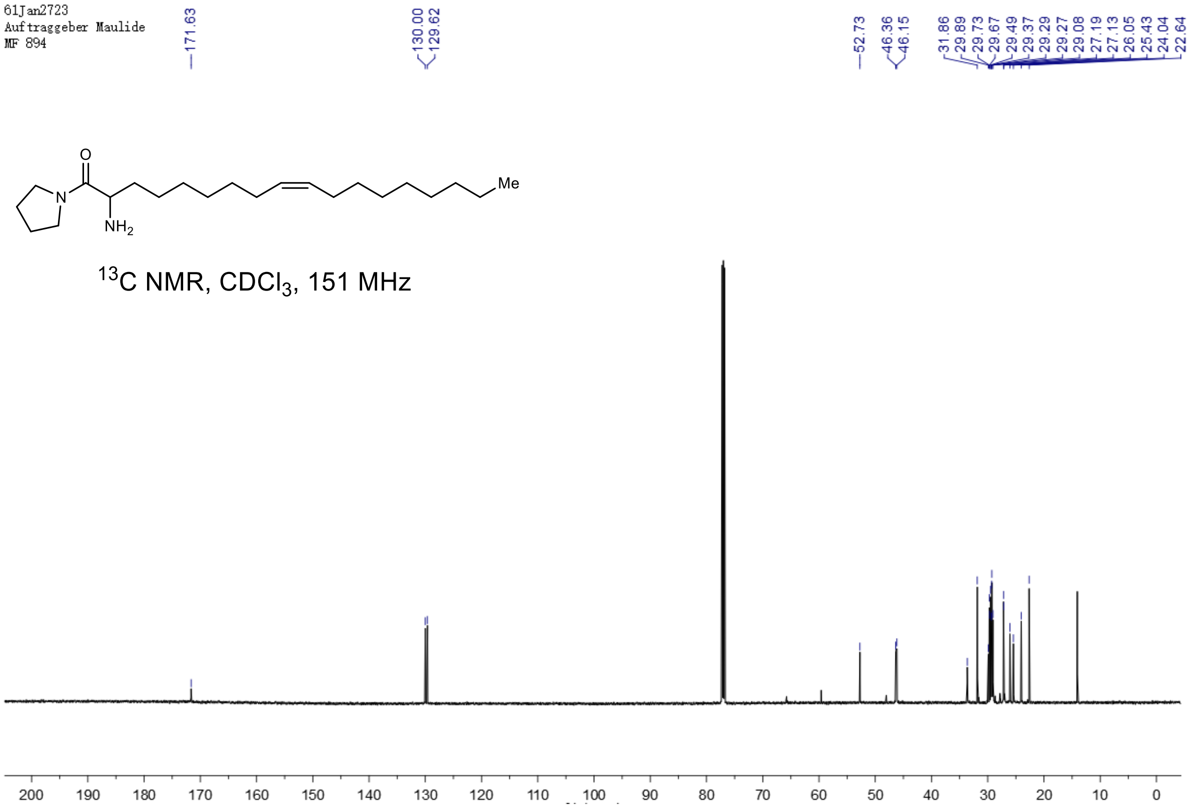

2-Amino-1-(indolyn-1-yl)hex-5-yn-1-one (4k)

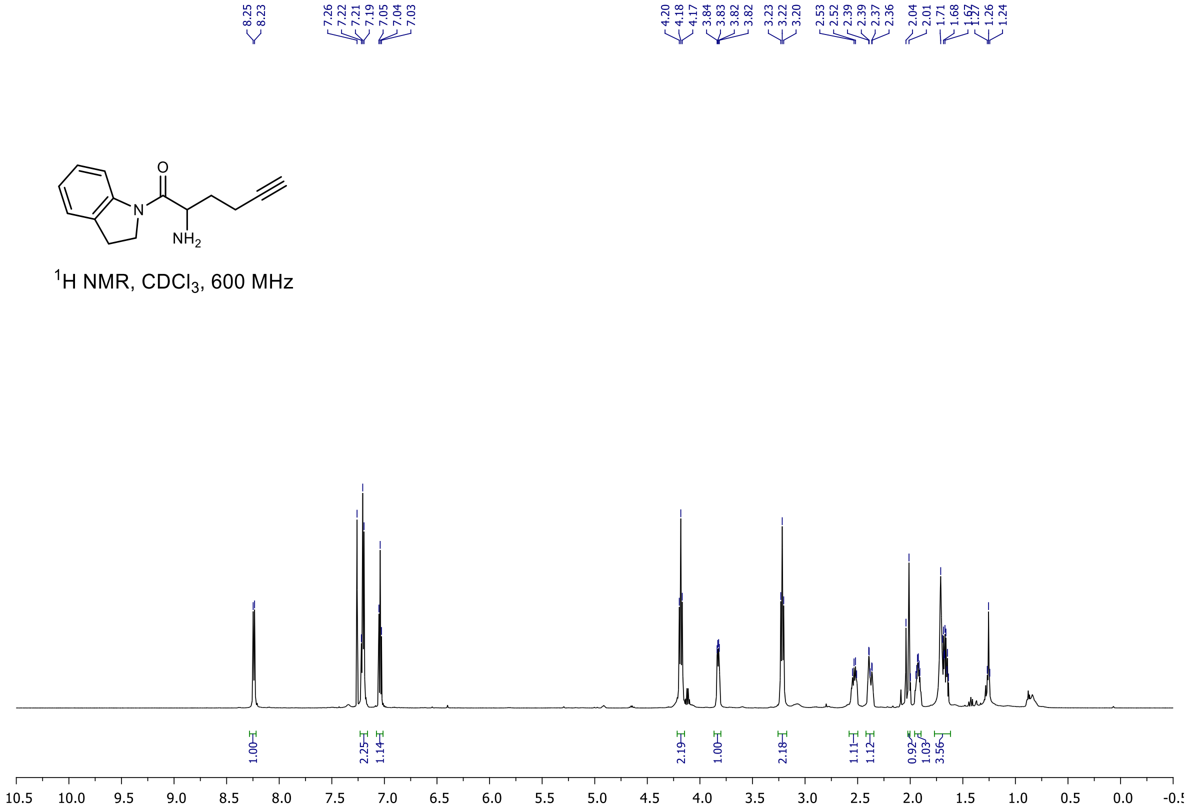

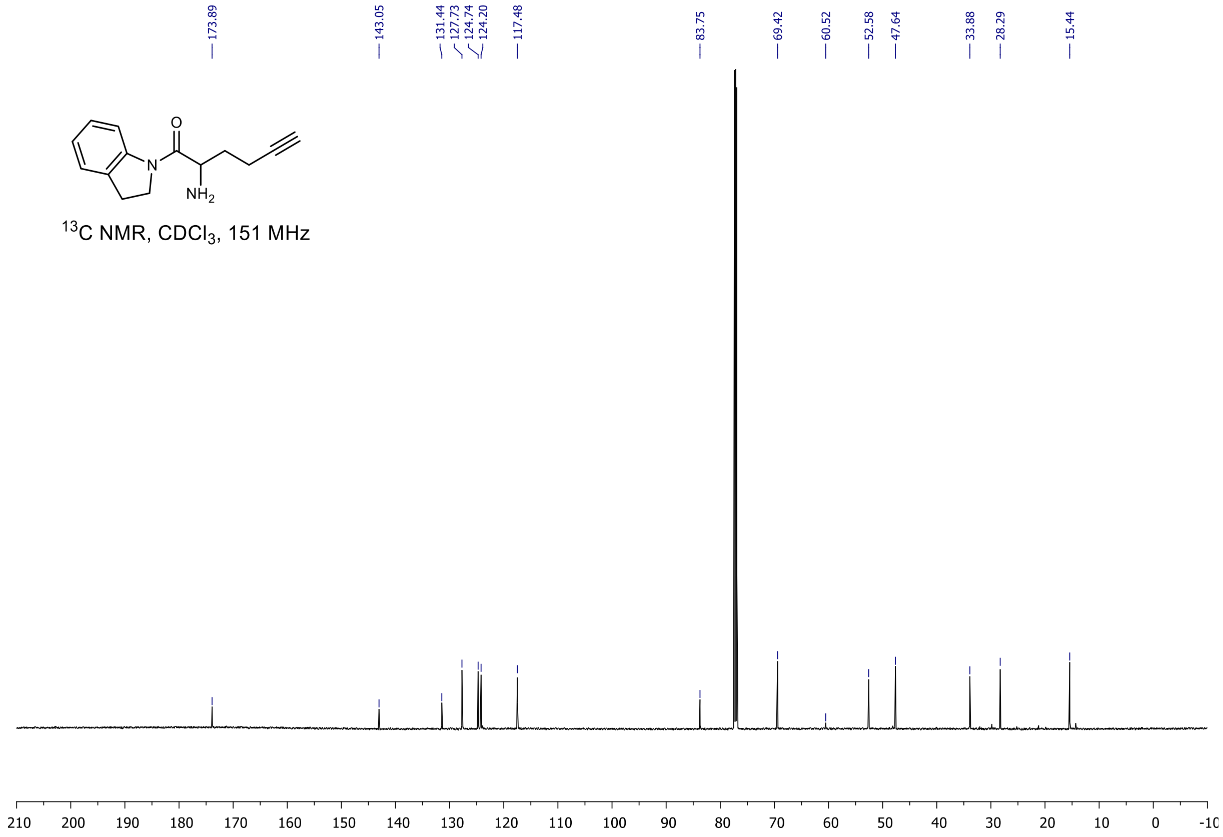

4-(Allyloxy)-2-amino-1-(pyrrolidin-1-yl)butan-1-one (4l)

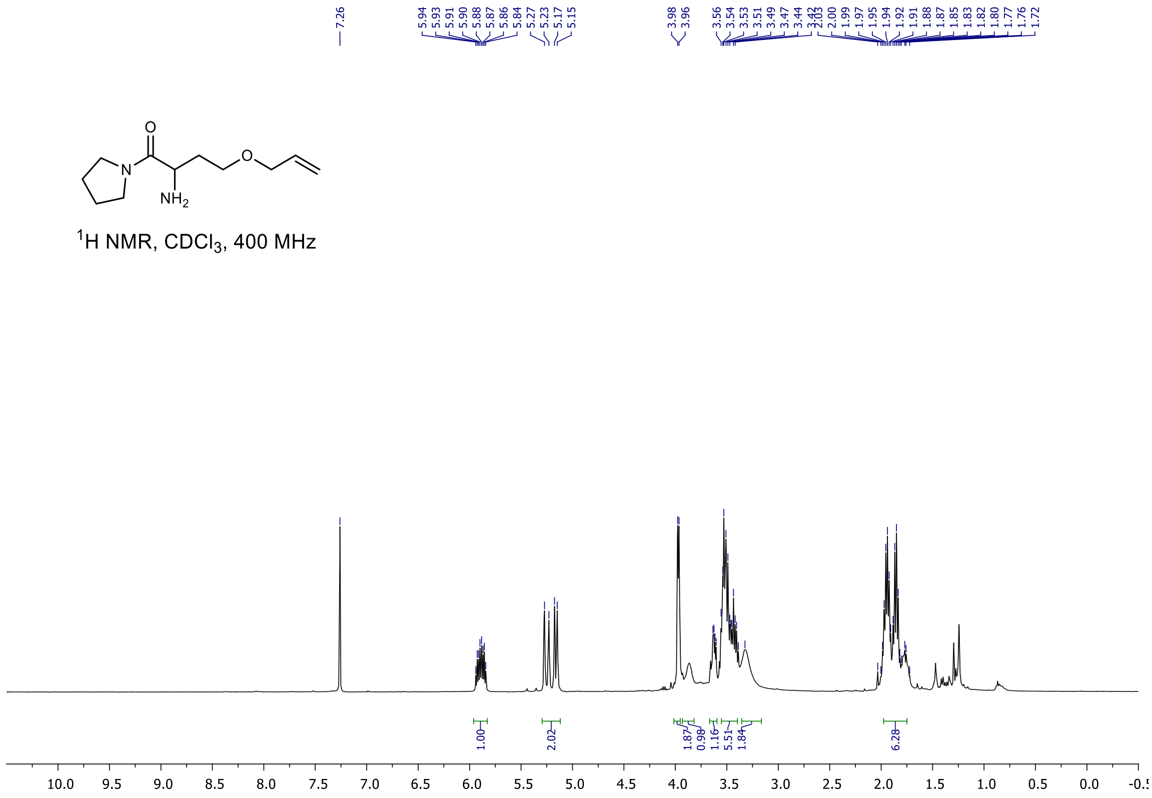

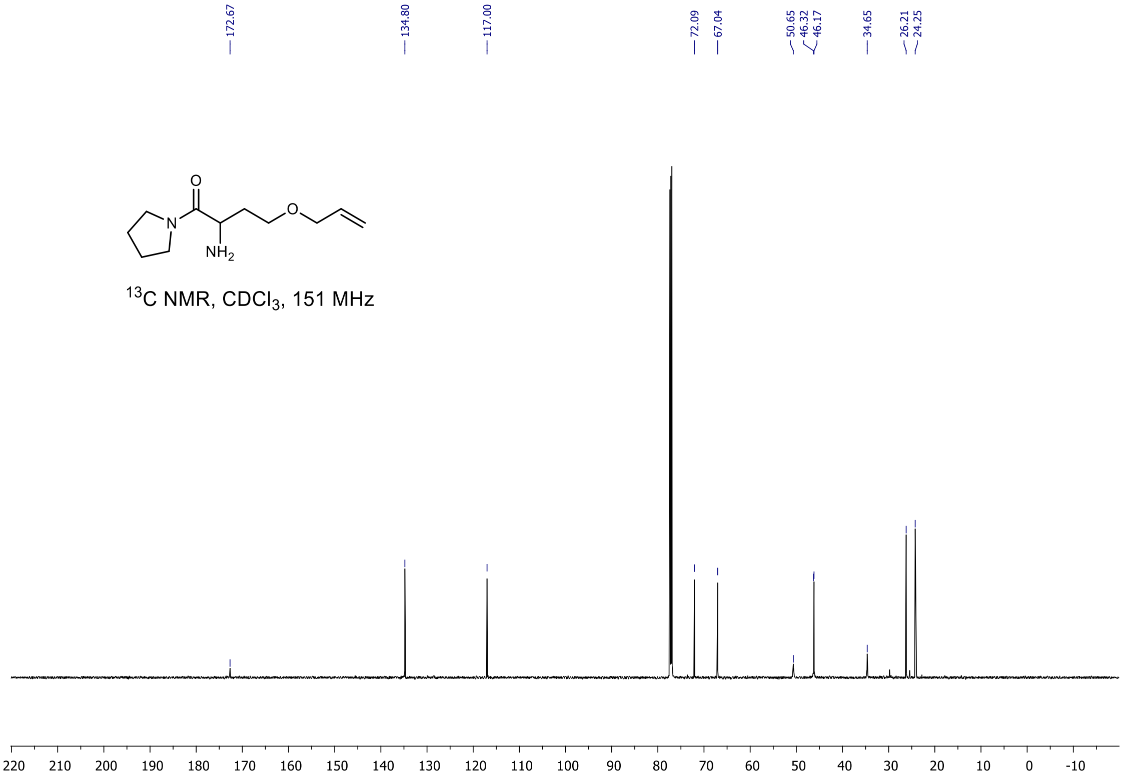

2-Amino-*N*,*N*-dimethyl-10-oxoundecanamide (4m)

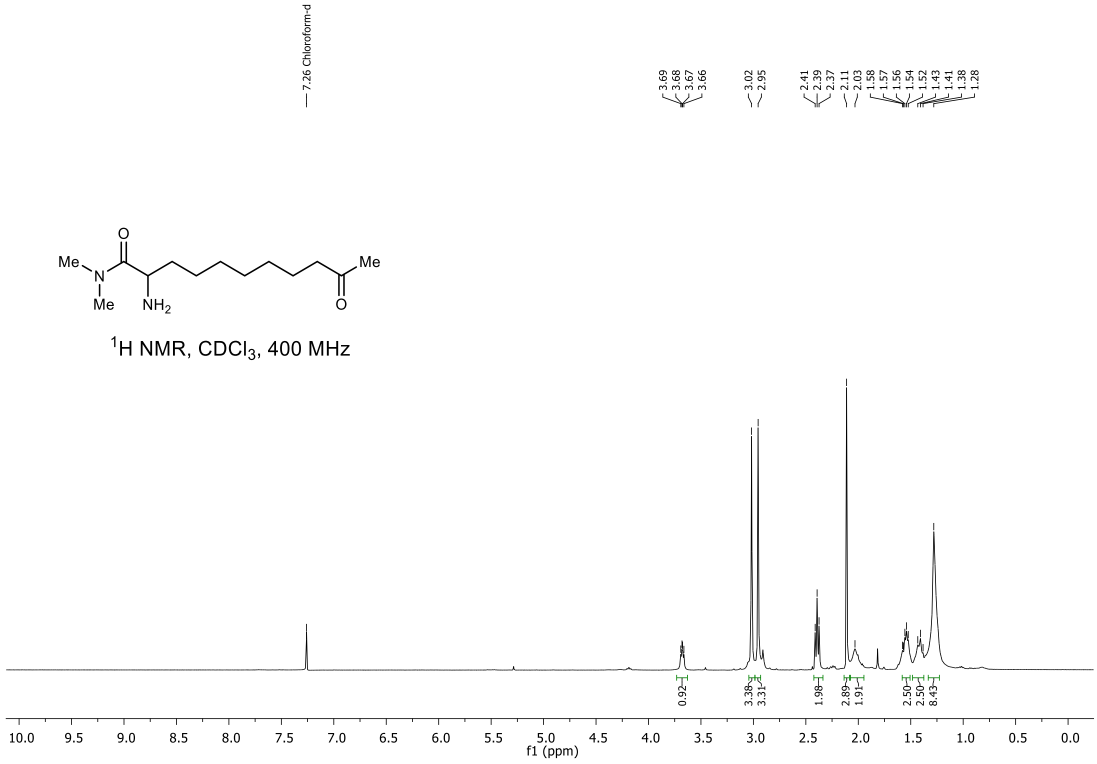

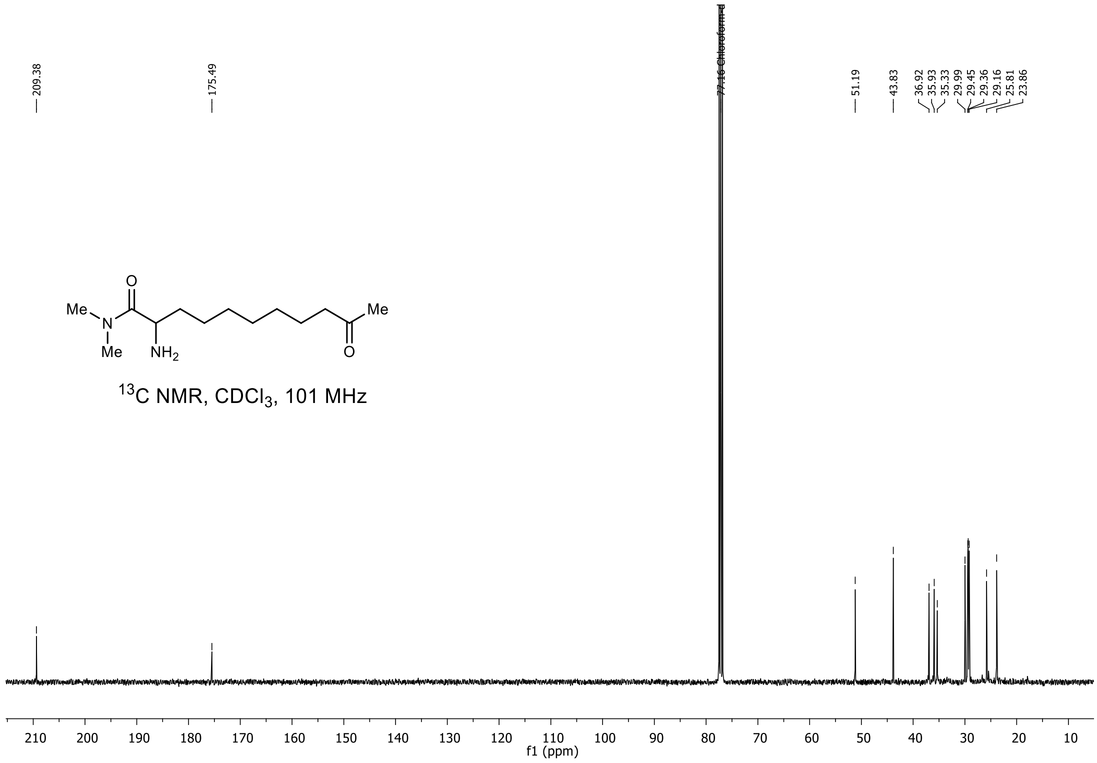

Methyl 8-amino-9-(indolin-1-yl)-9-oxononanoate (4n)

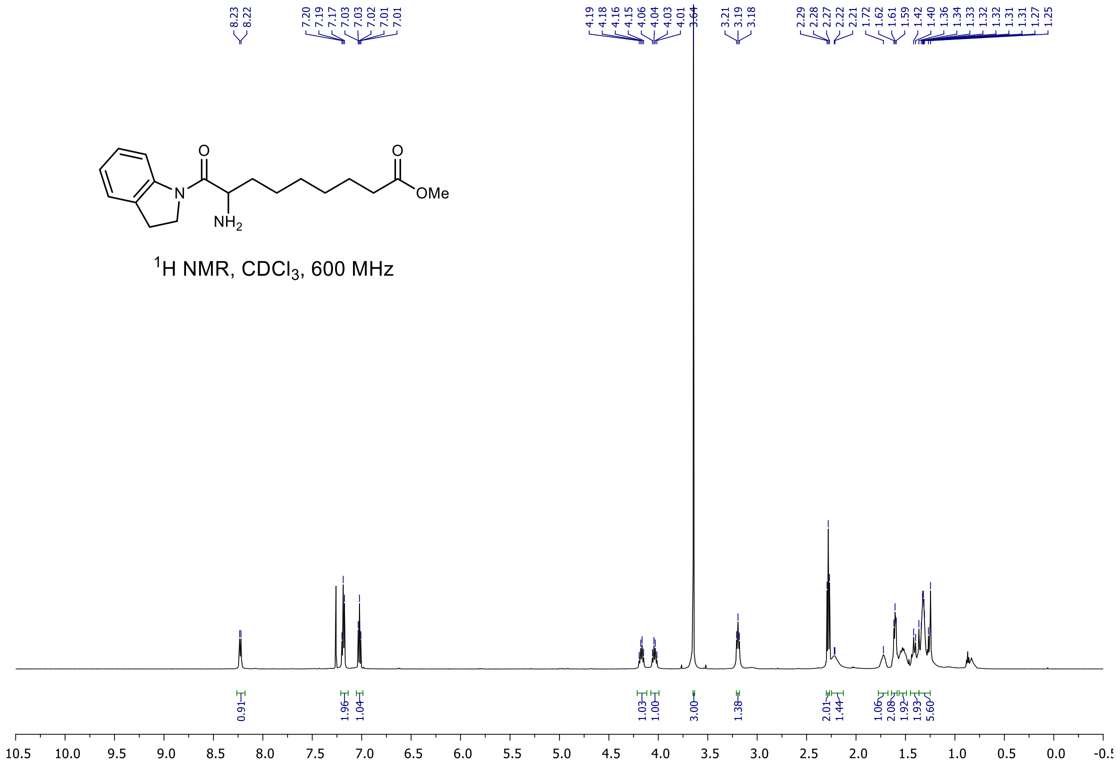

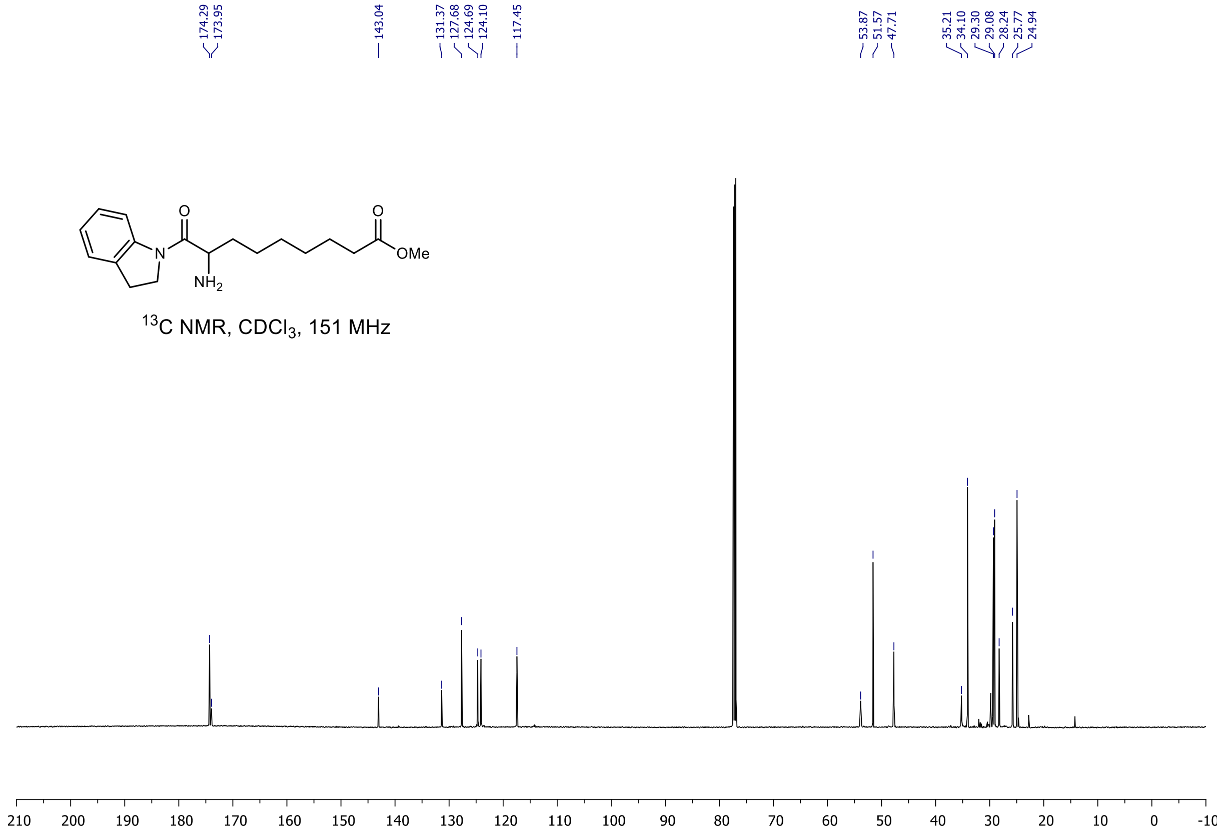

2-Amino-6-cyano-*N,N*-dimethylhexanamide (4.0)

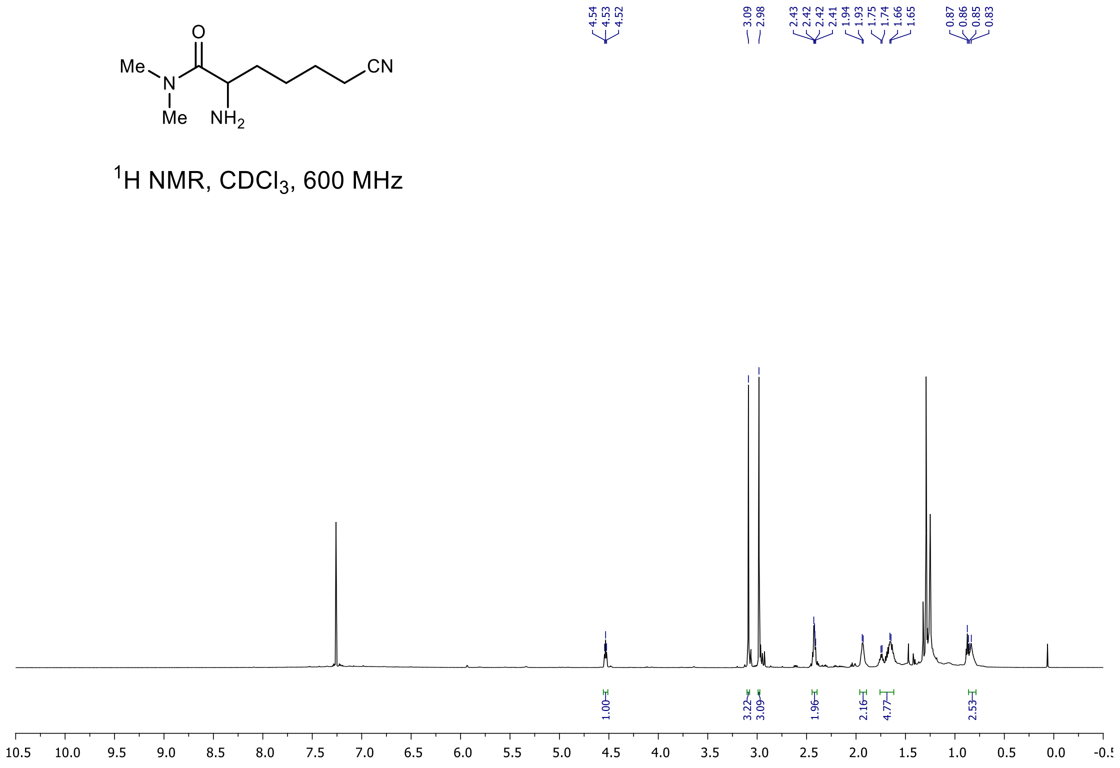

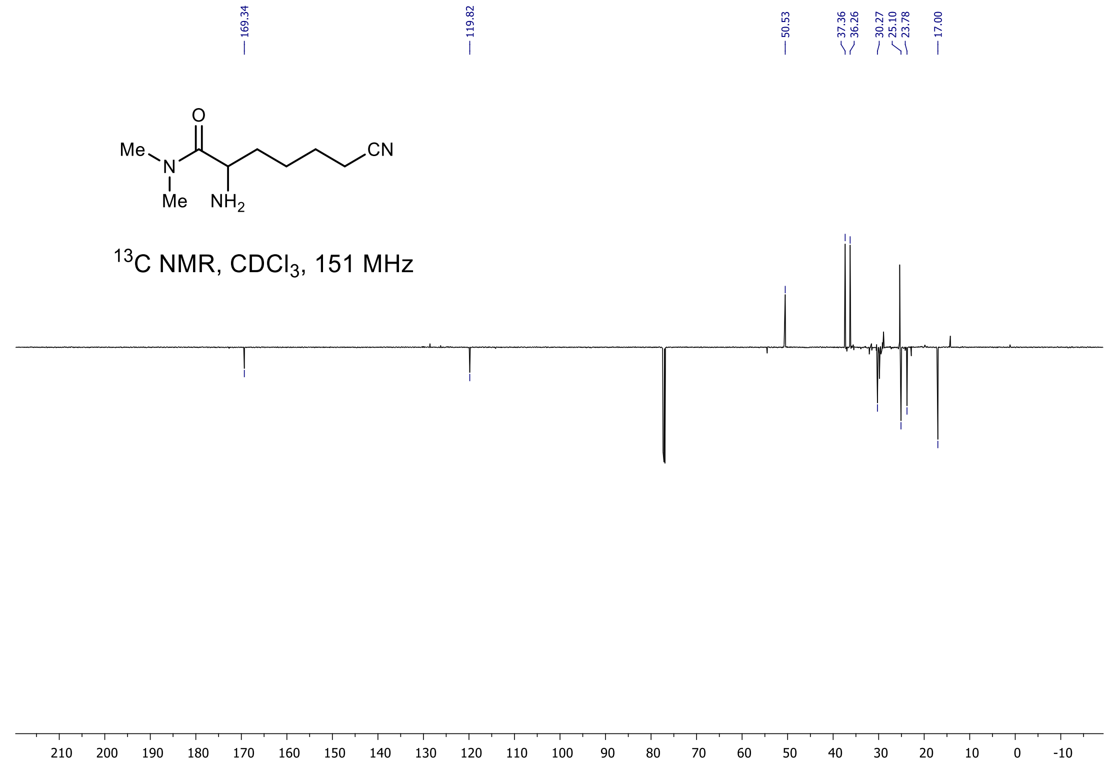

2-Amino-6-(1,3-dioxoisondolin-2-yl)-*N*,*N*-dimethylhexanamide (4p)

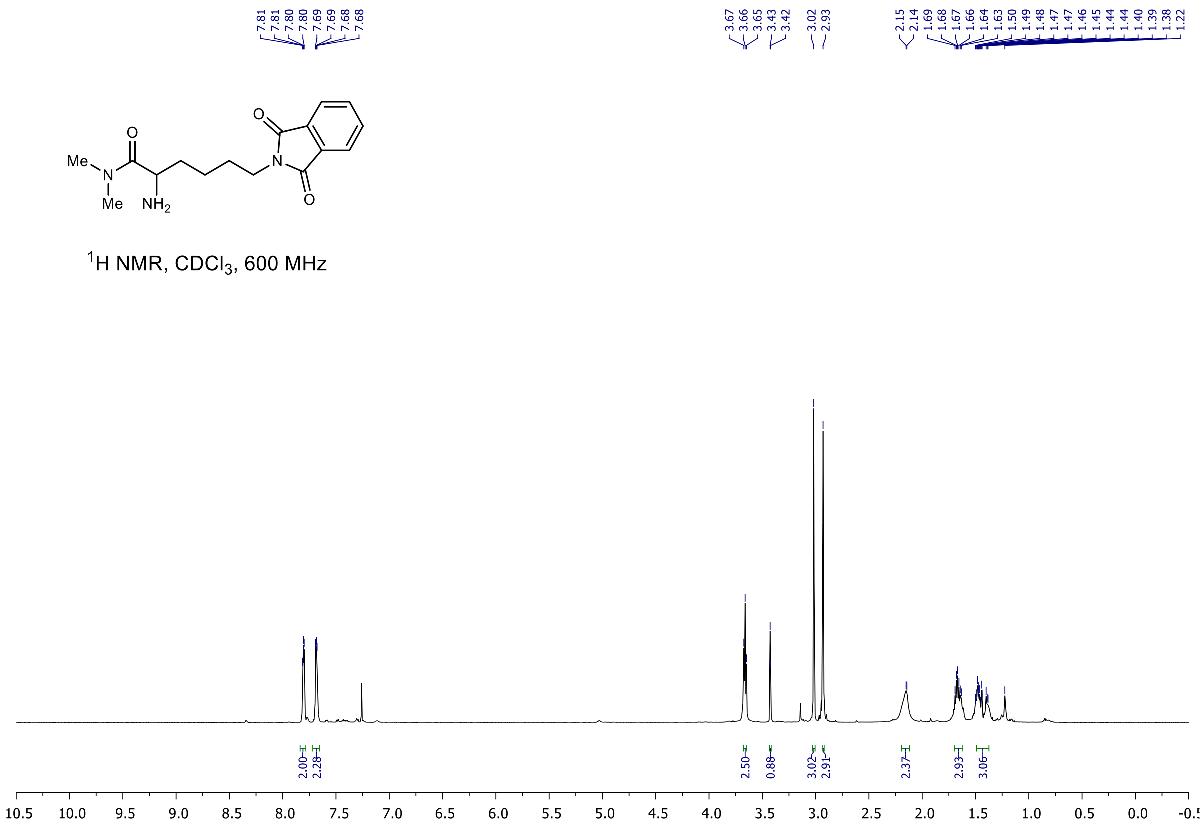

^{1}H NMR, $CDCl_3$, 600 MHz

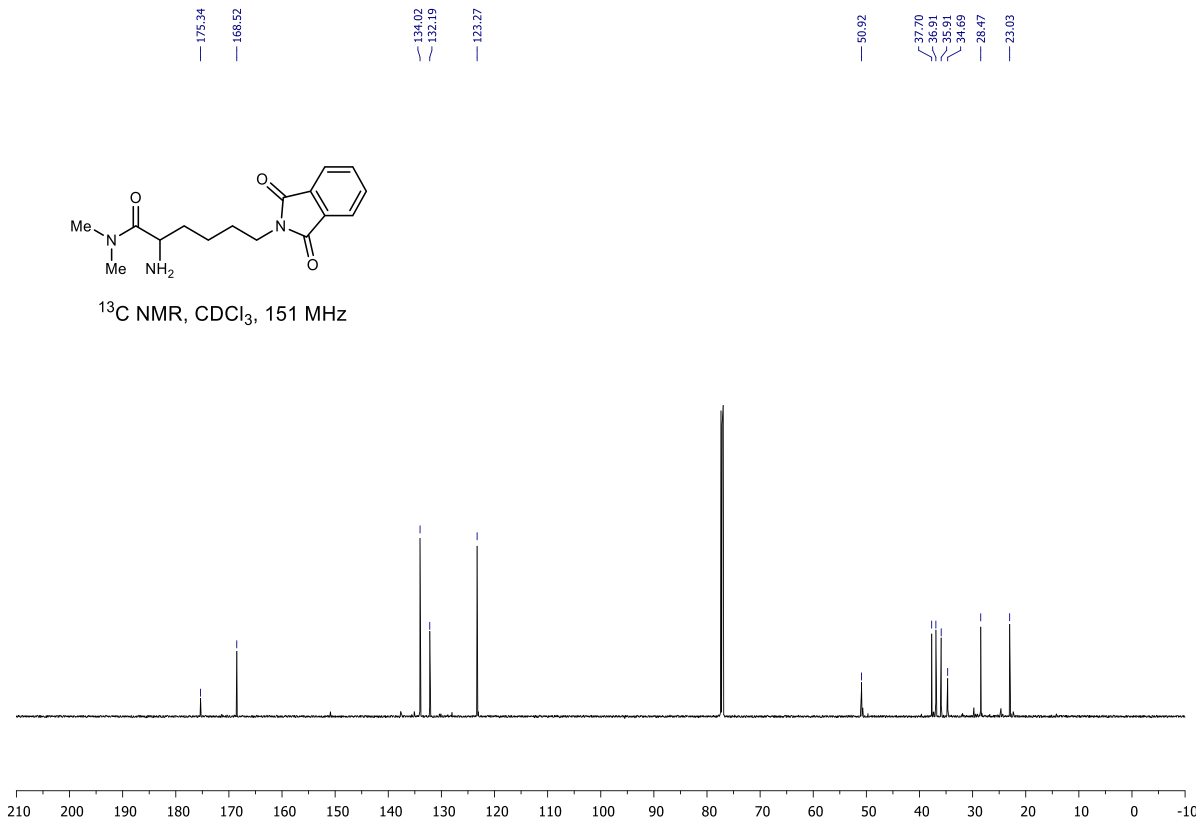

^{13}C NMR, $CDCl_3$, 151 MHz

2-Amino-*N*,*N*-dimethyl-3-(thiophen-2-yl)propanamide (4q)

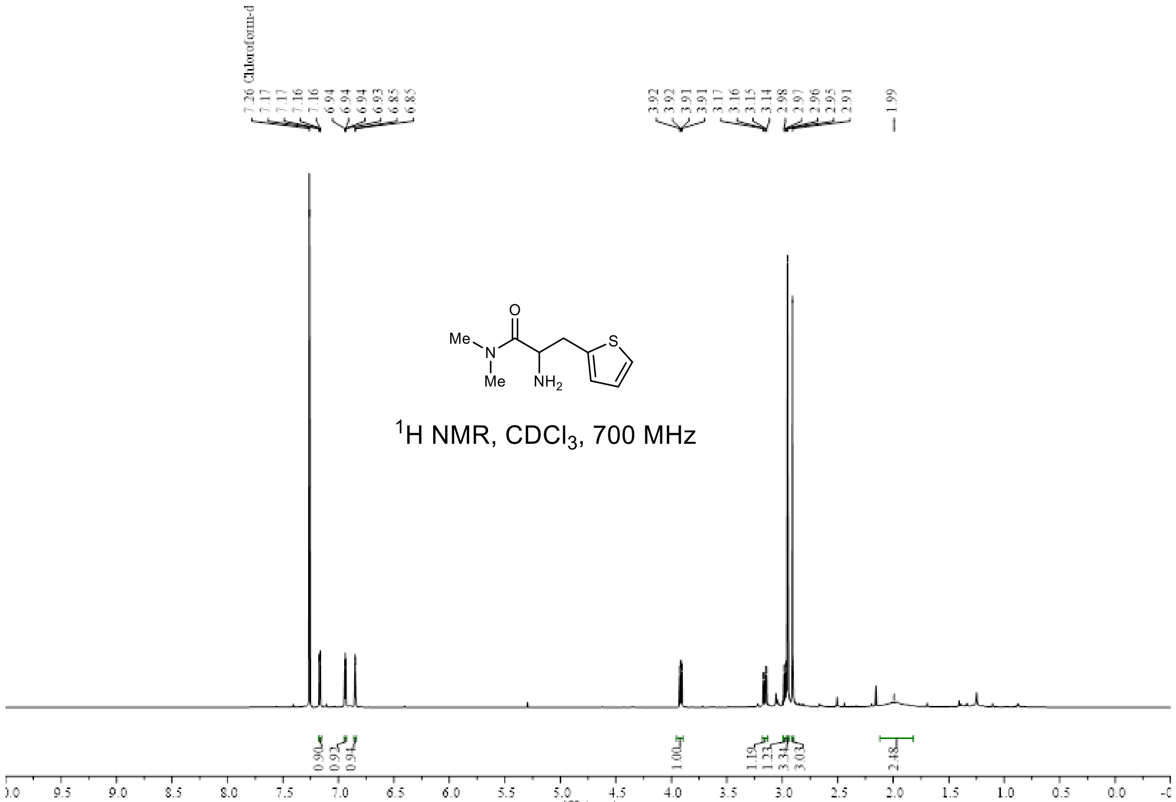

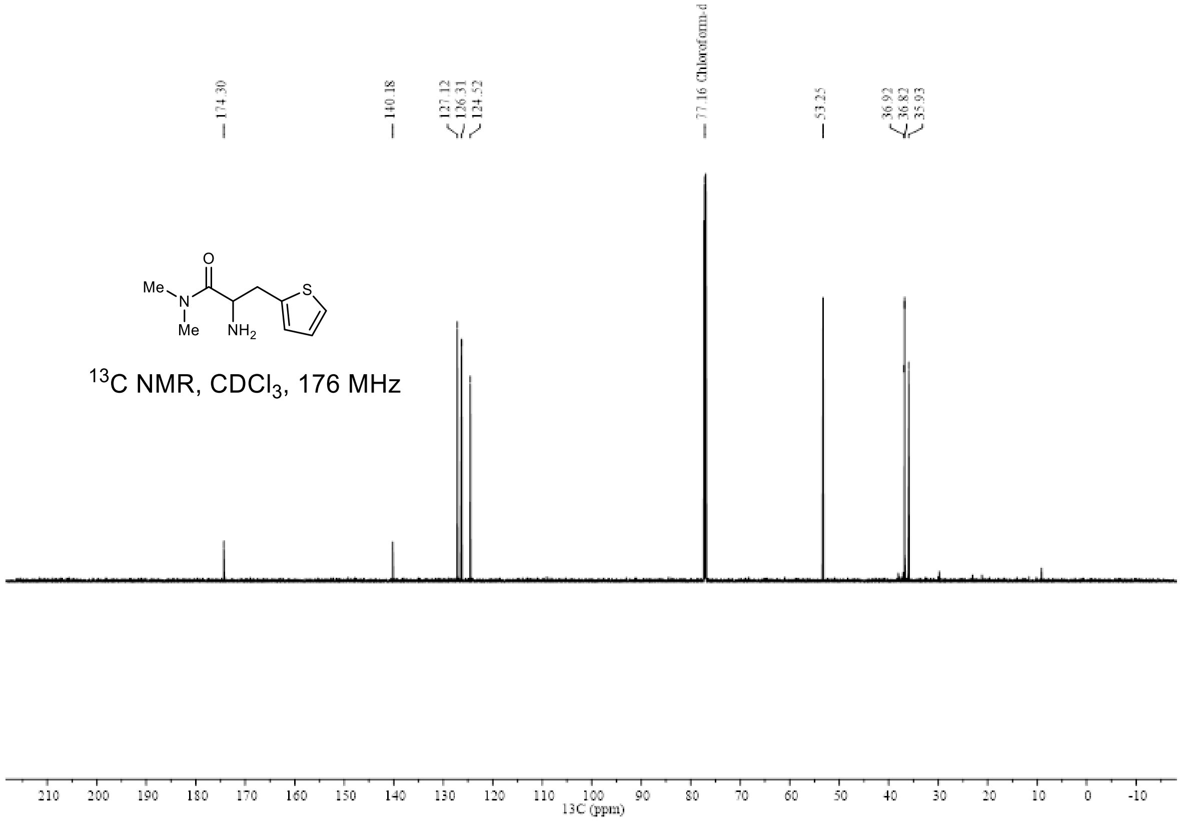

2-Amino-3-(benzo[*b*]thiophen-2-yl)-*N,N*-dimethylpropanamide (4r)

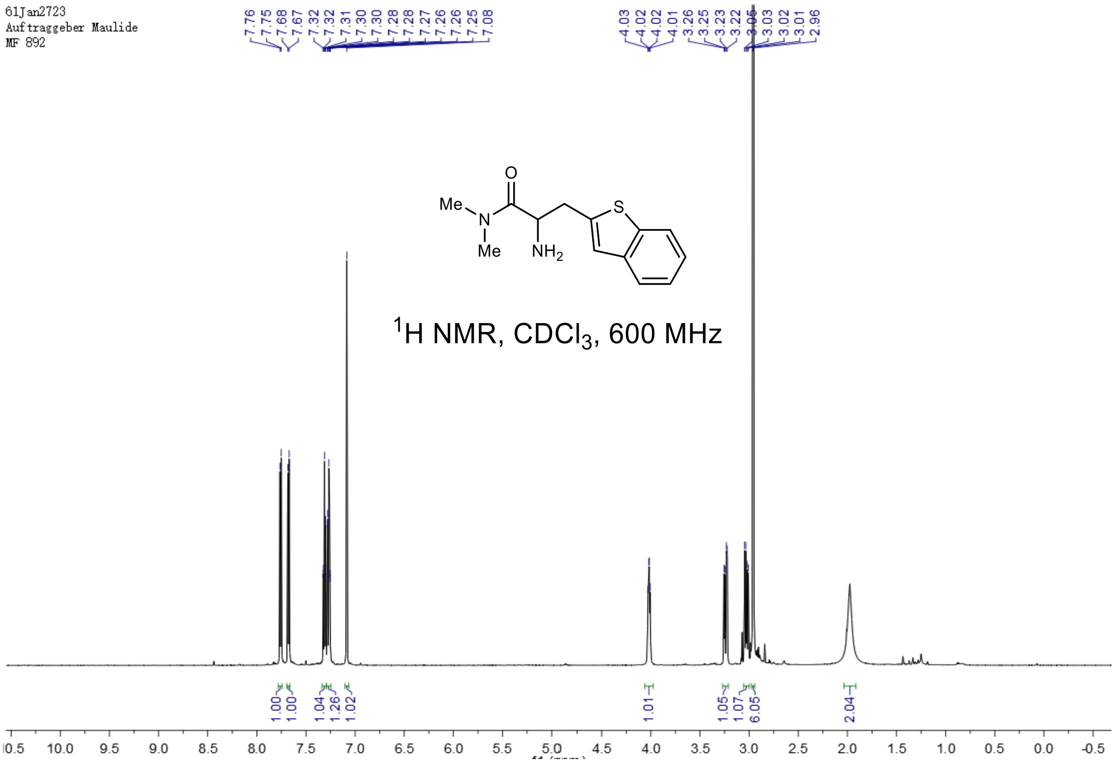

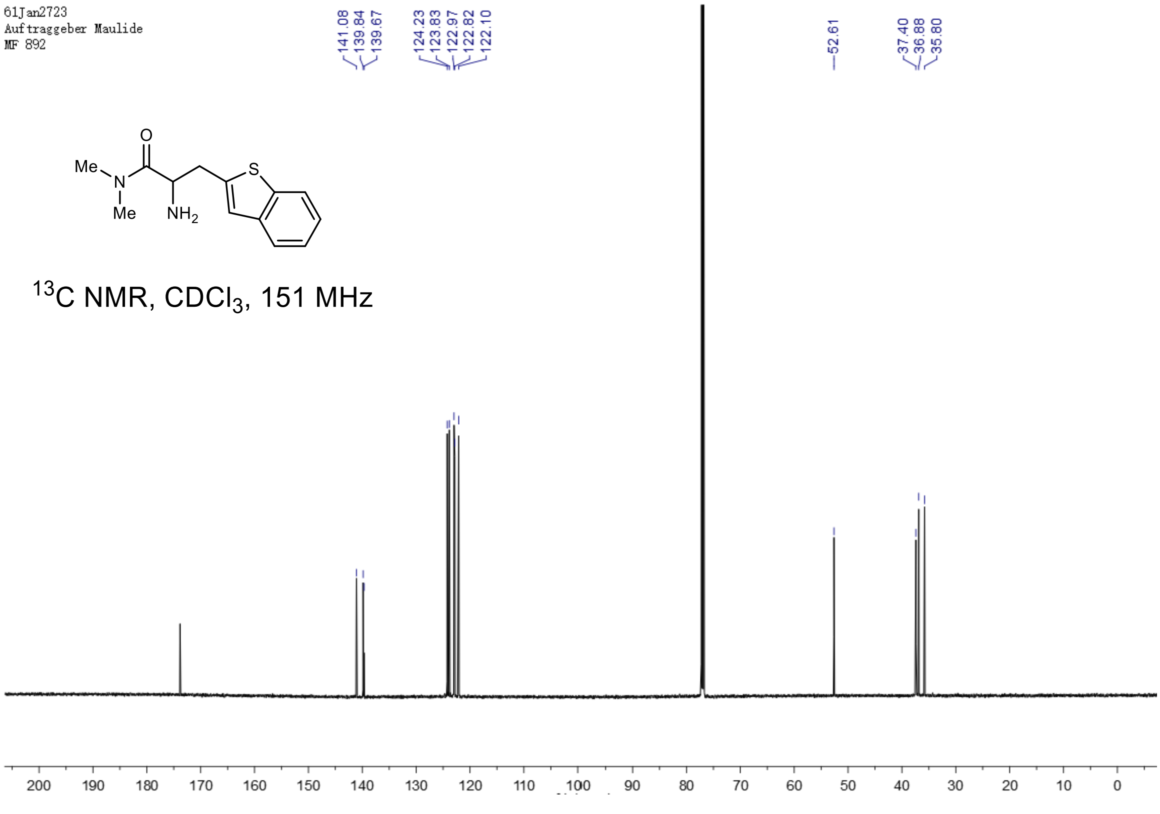

2-Amino-1-(5-methoxyindolin-1-yl)-3-phenylpropan-1-one (4s)

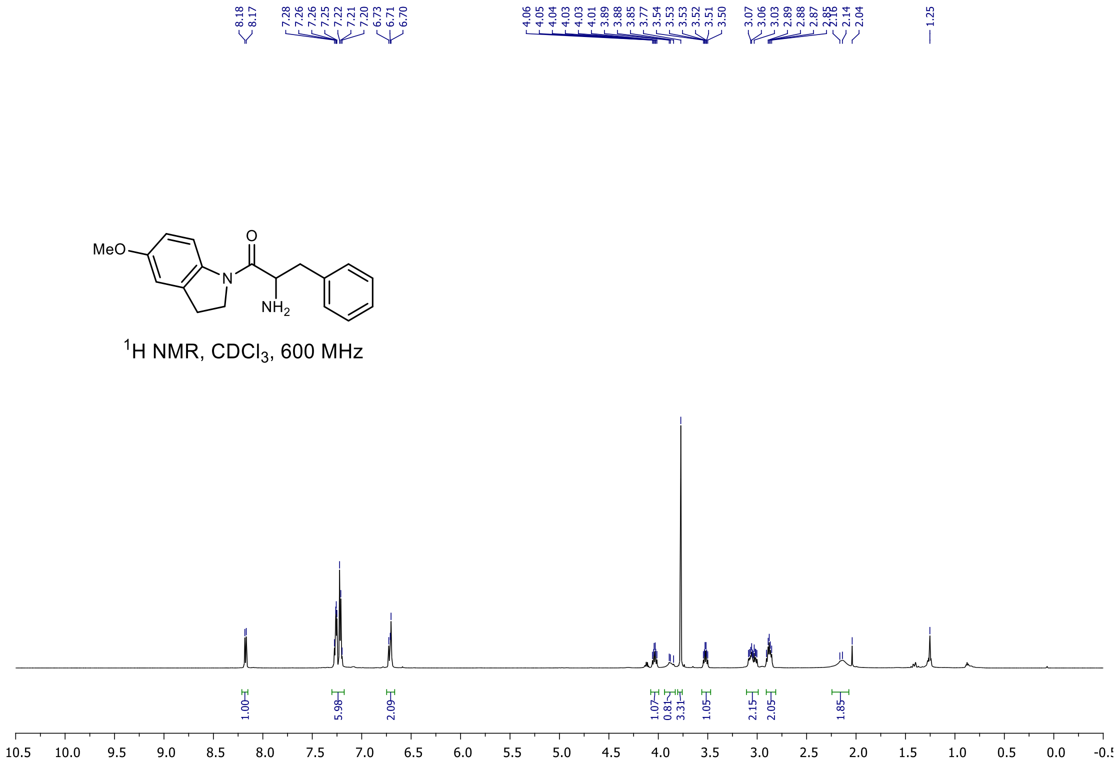

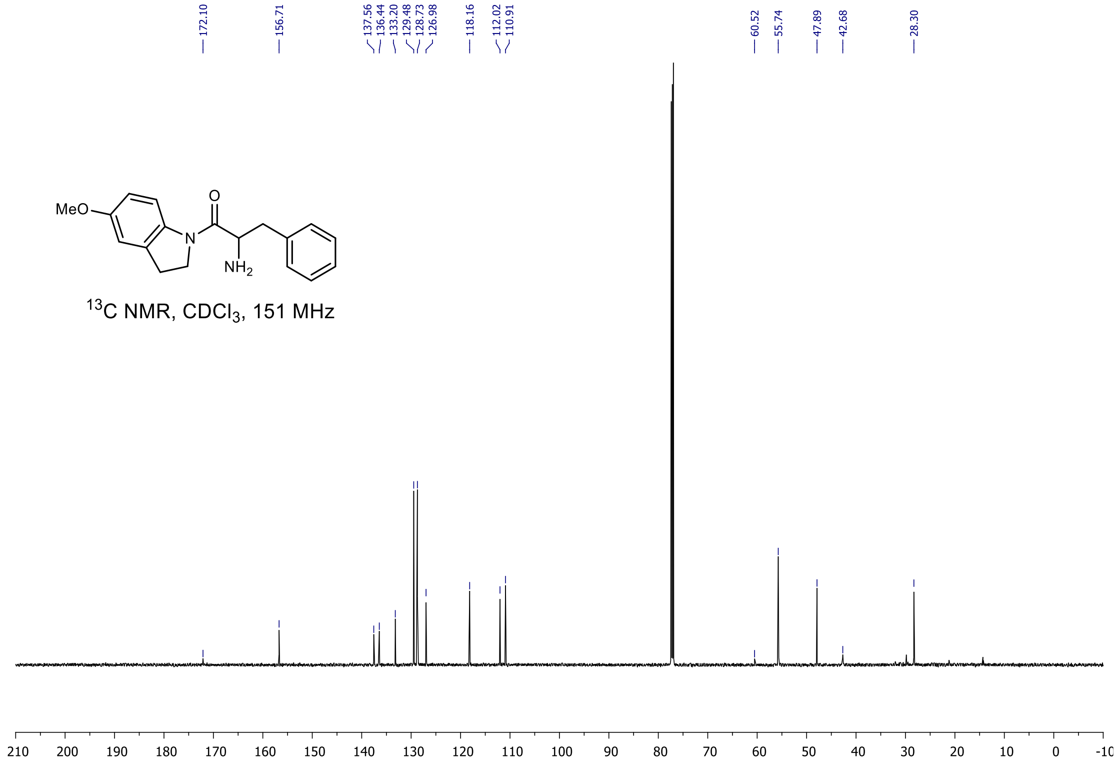

2-Amino-1-(indolin-1-yl)-3-(4-(trifluoromethyl)phenyl)propan-1-one (4t)

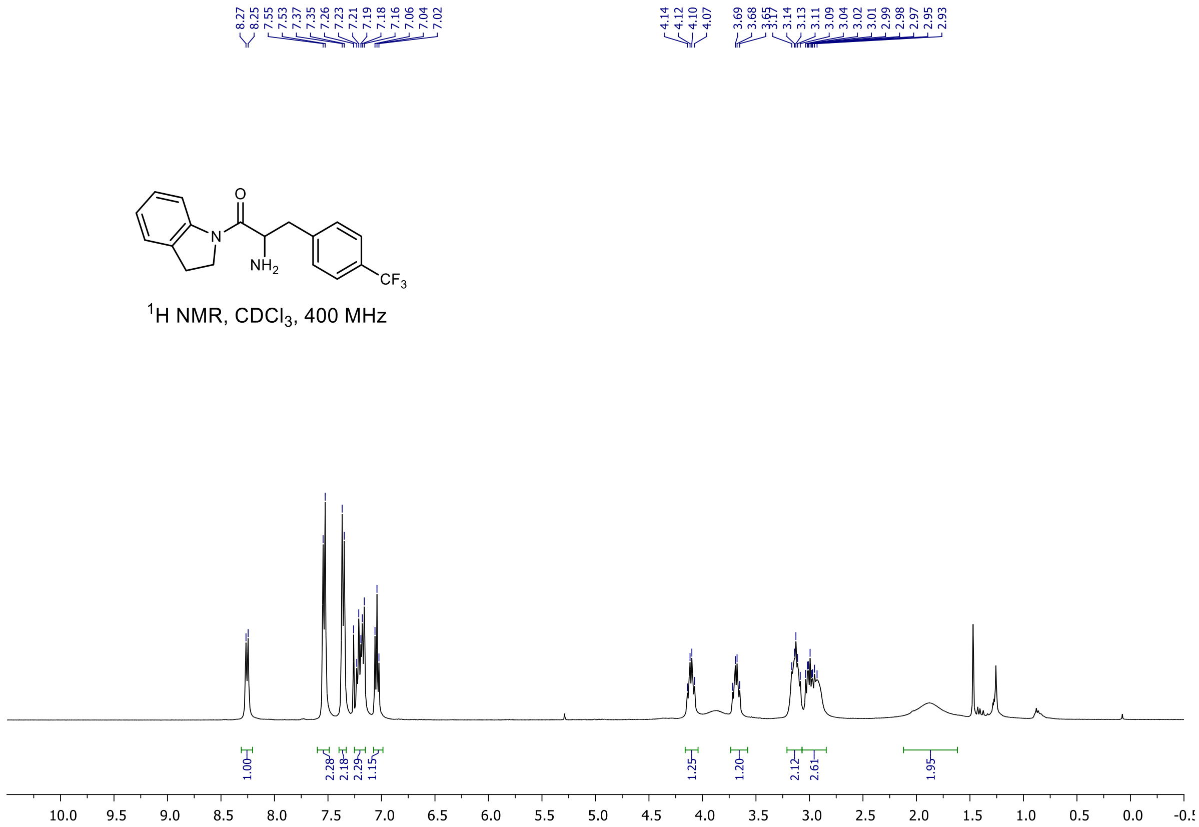

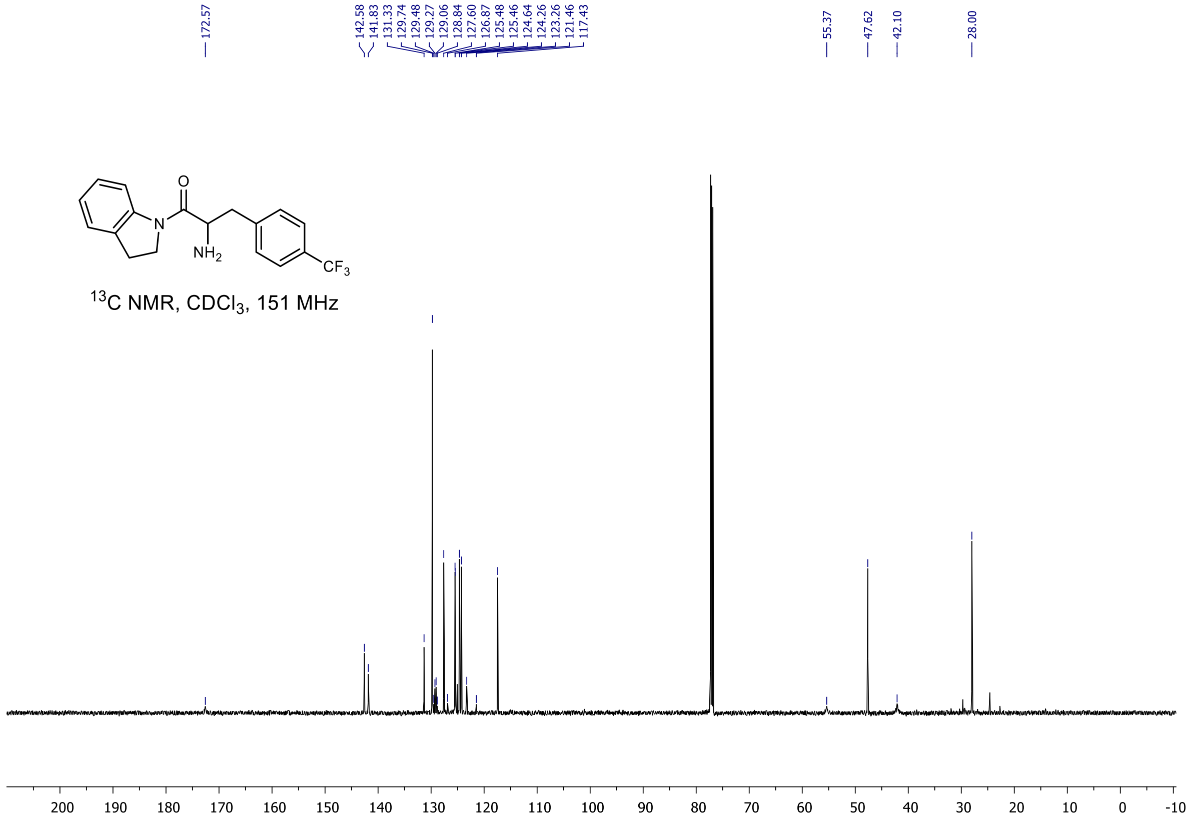

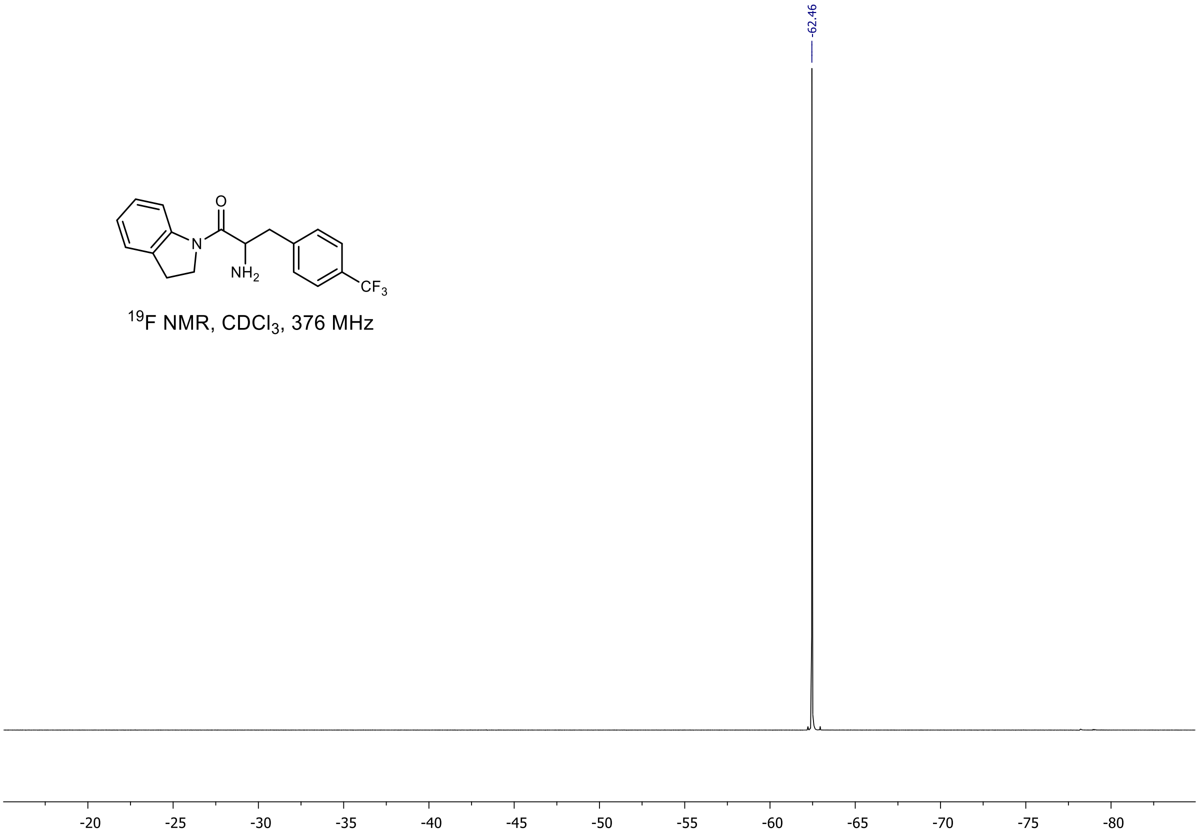
-62.46
O
N
NH2
CF3
19F NMR, CDCl3, 376 MHz
-20
-25
-30
-35
-40
-45
-50
-55
-60
-65
-70
-75
-80

2-Amino-2-cyclopropyl-1-(indolin-1-yl)ethan-1-one (4u)

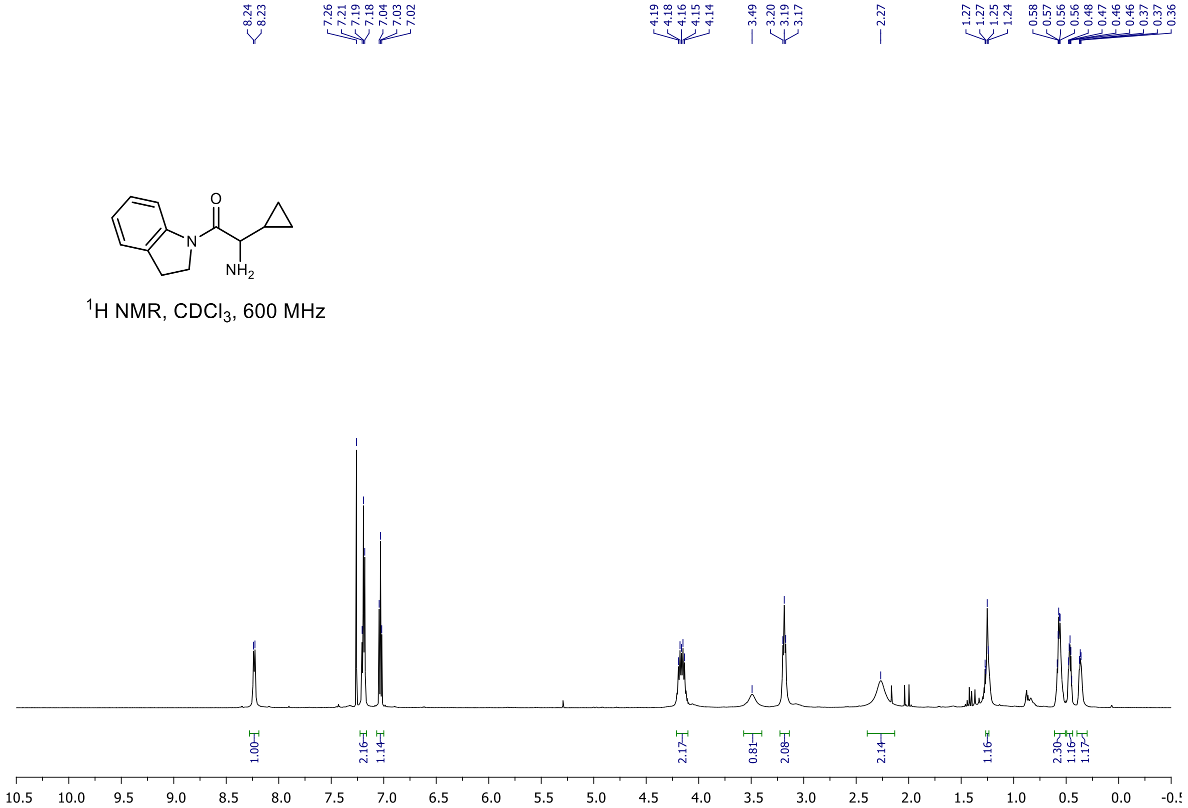

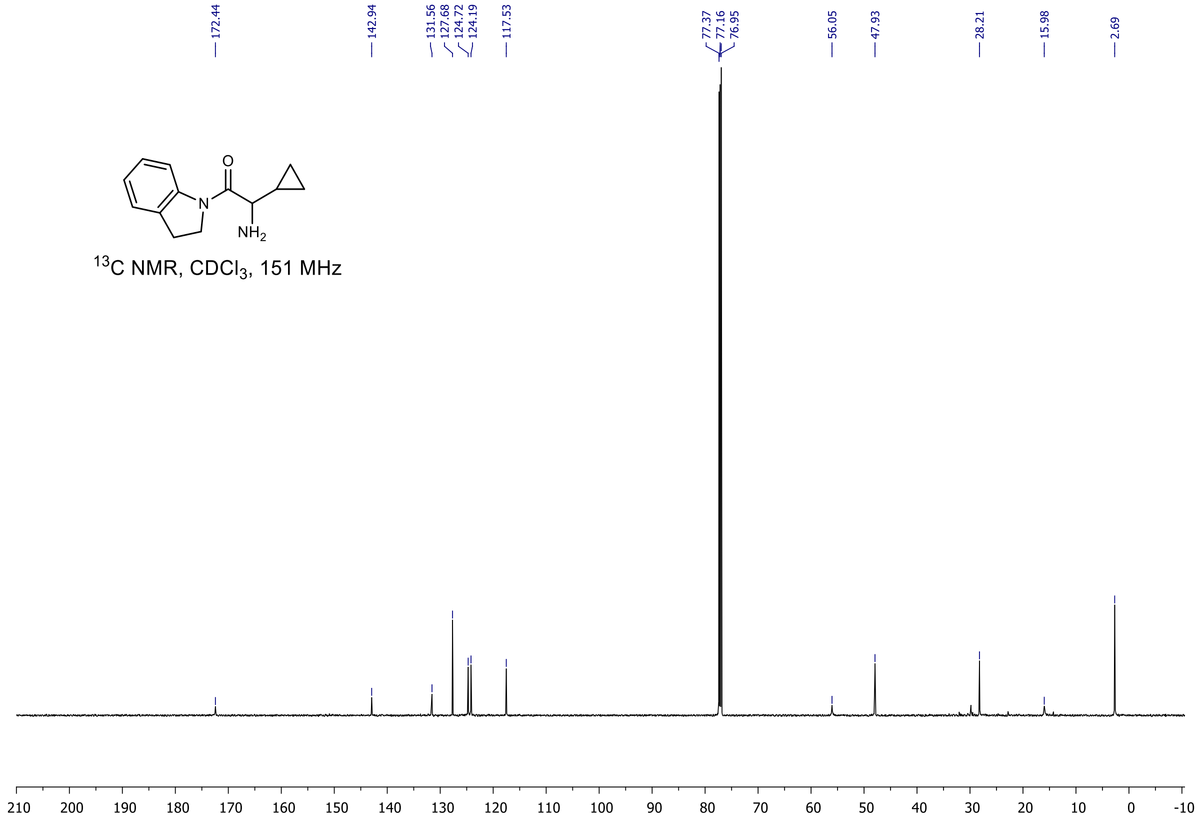

2-Amino-1-methylazacyclotridecan-2-one (4v)

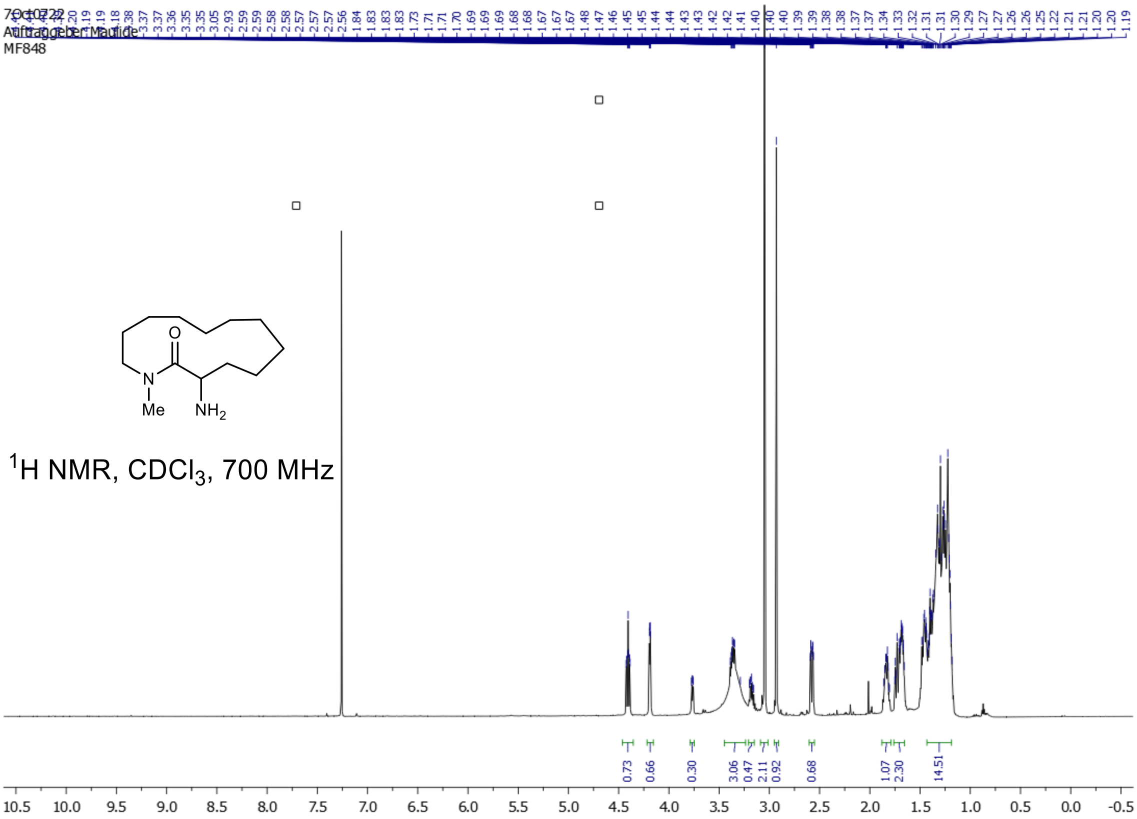

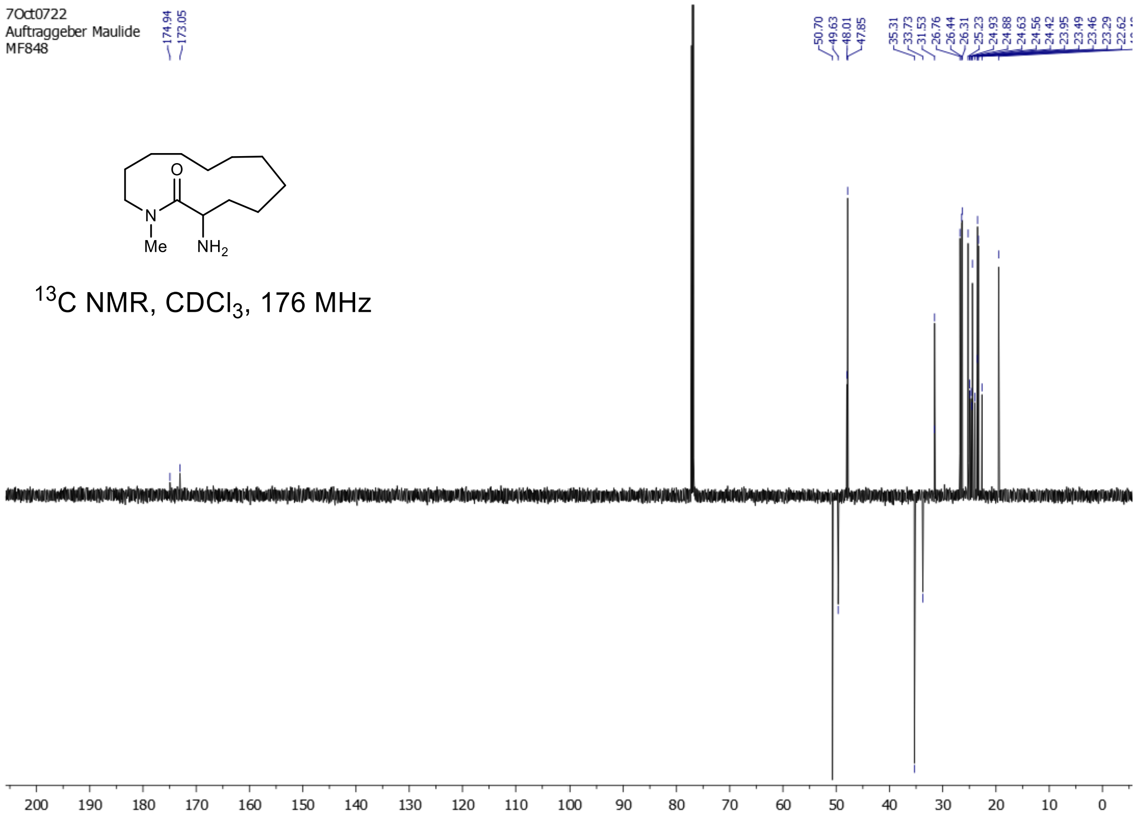

***N*-(2-(4-Methoxyphenyl)-2-oxoethyl)benzamide (5a)**

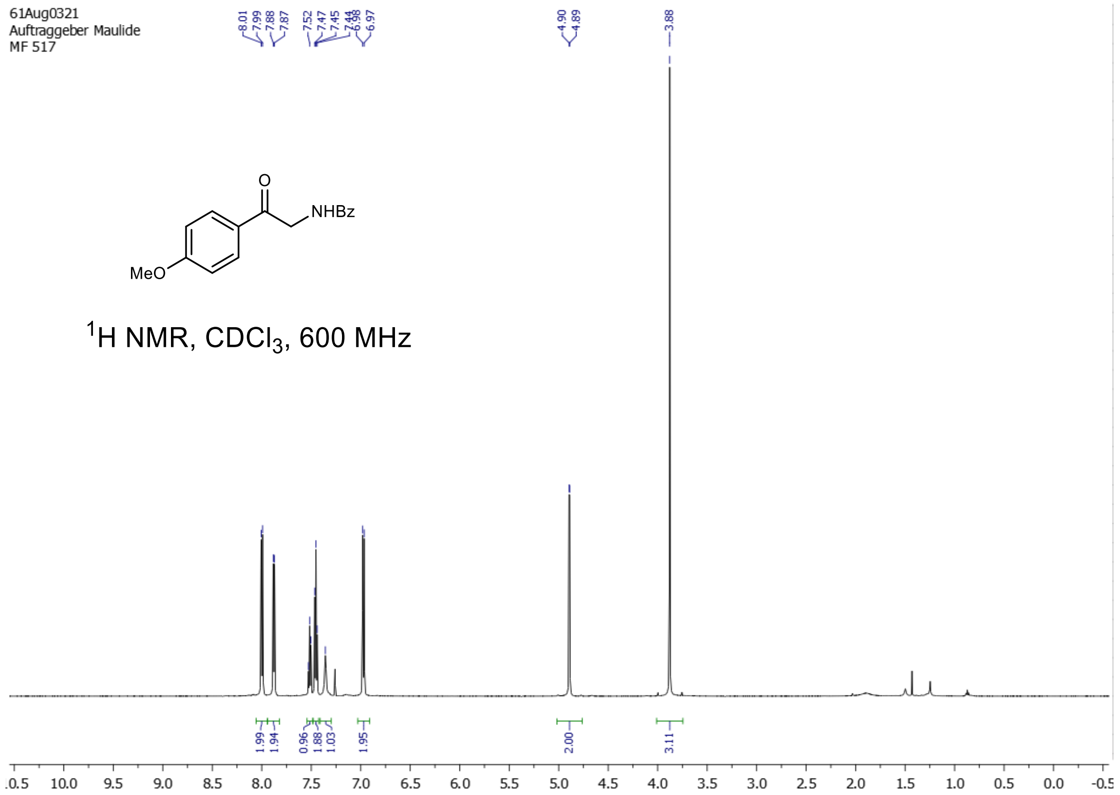

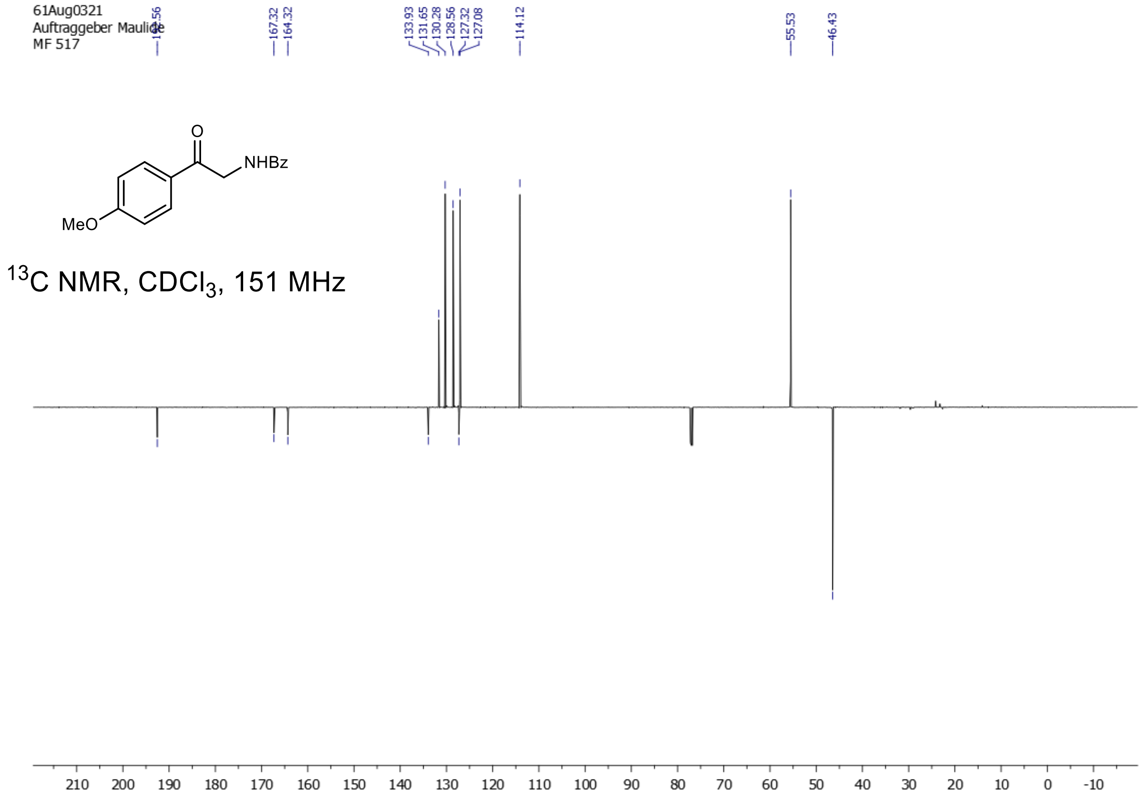

N-(2-(3,4-Dimethoxyphenyl)-2-oxoethyl)benzamide (5b)

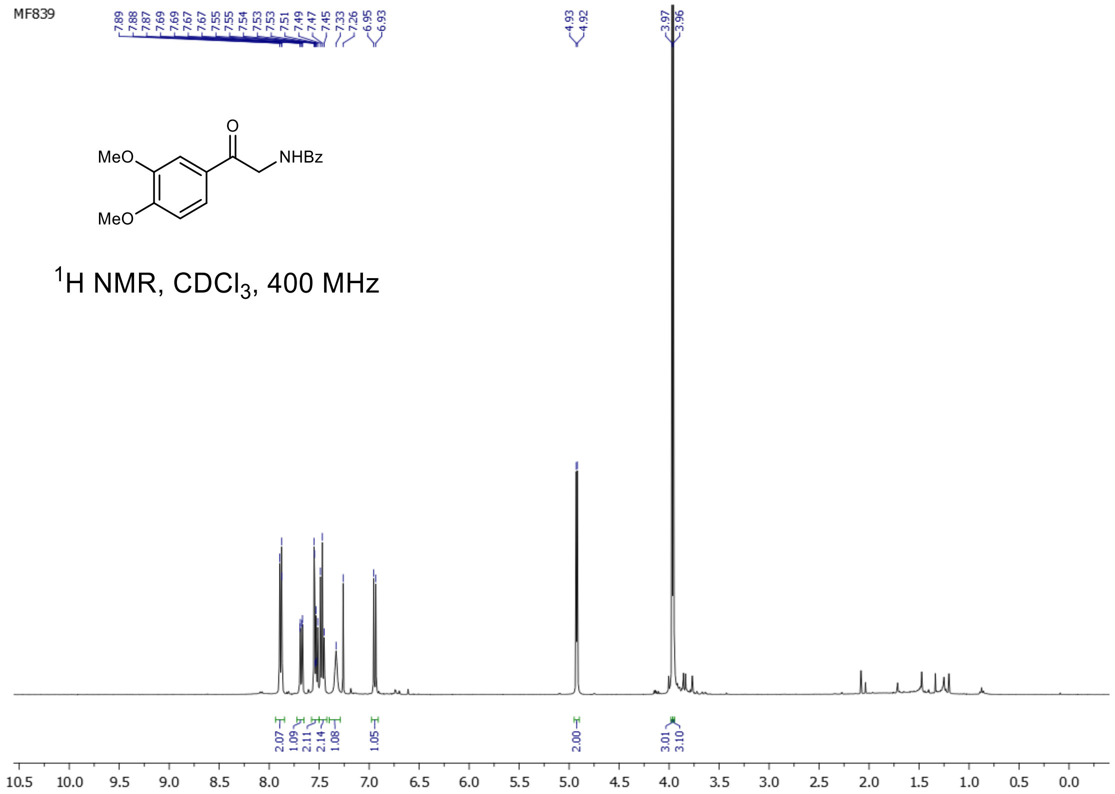

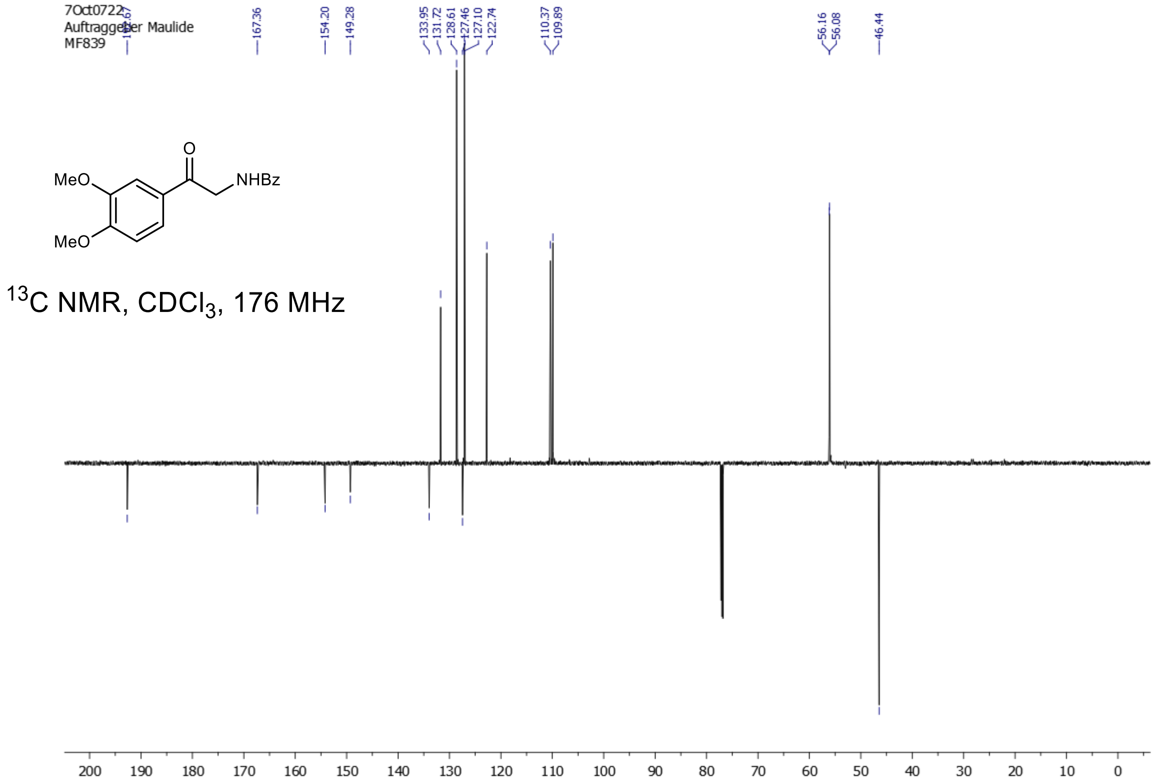

N-(2-(2,3-Dihydrobenzo[b][1,4]dioxin-6-yl)-2-oxoethyl)benzamide (5c)

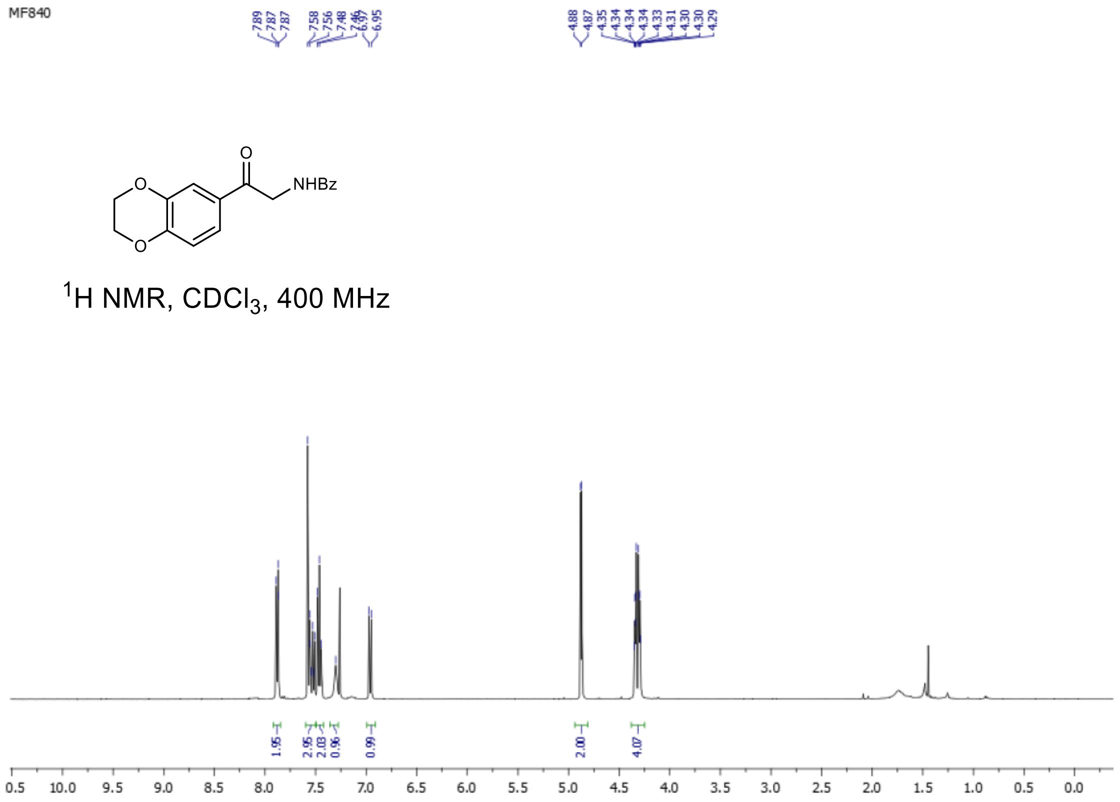

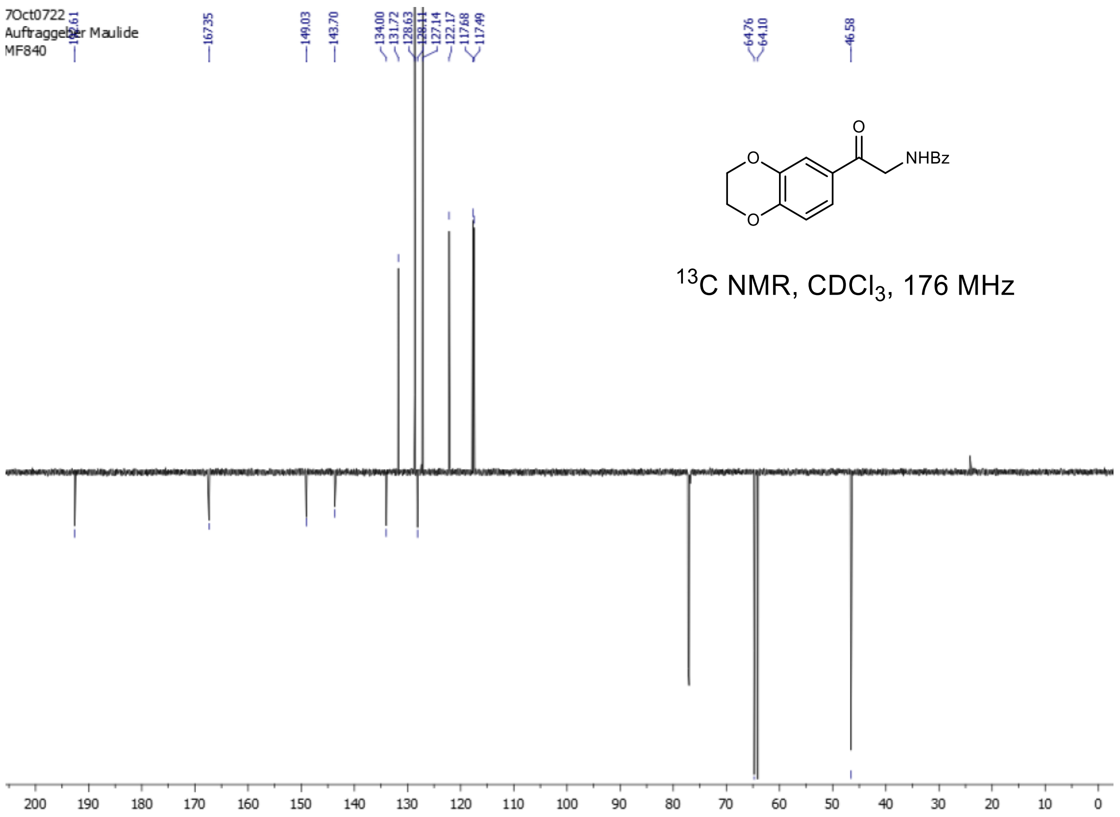

N-(2-Oxo-2-(3,4,5-trimethoxyphenyl)ethyl)benzamide (5d)

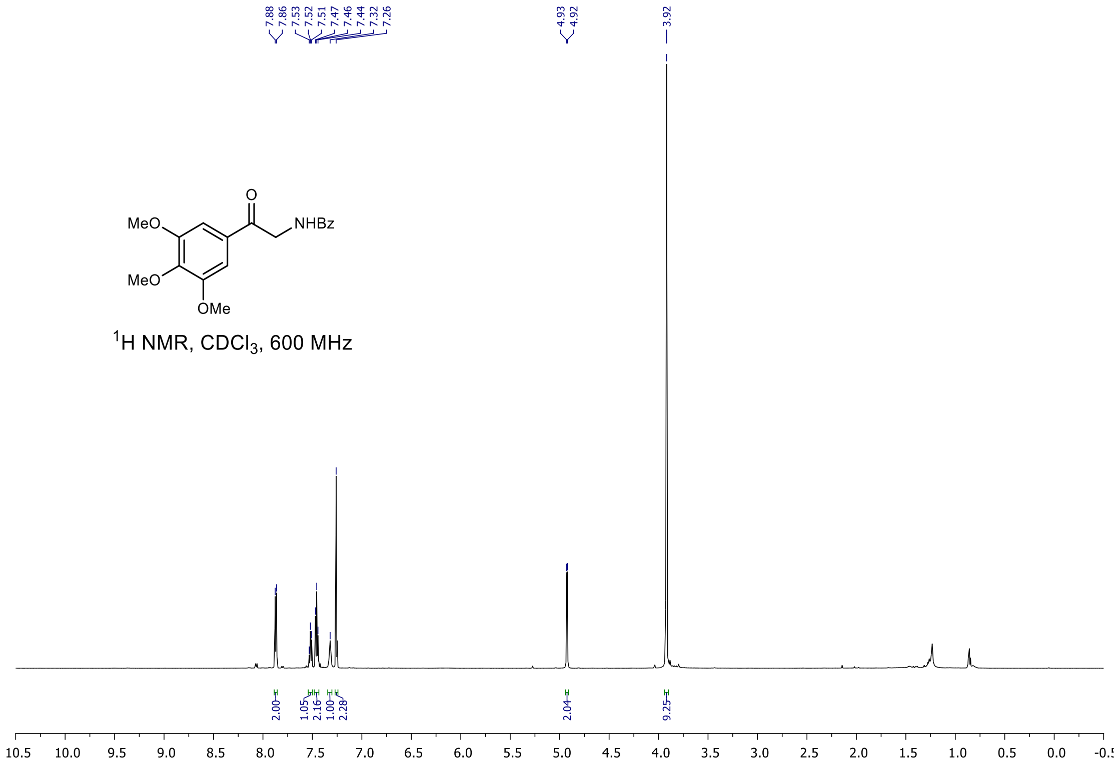

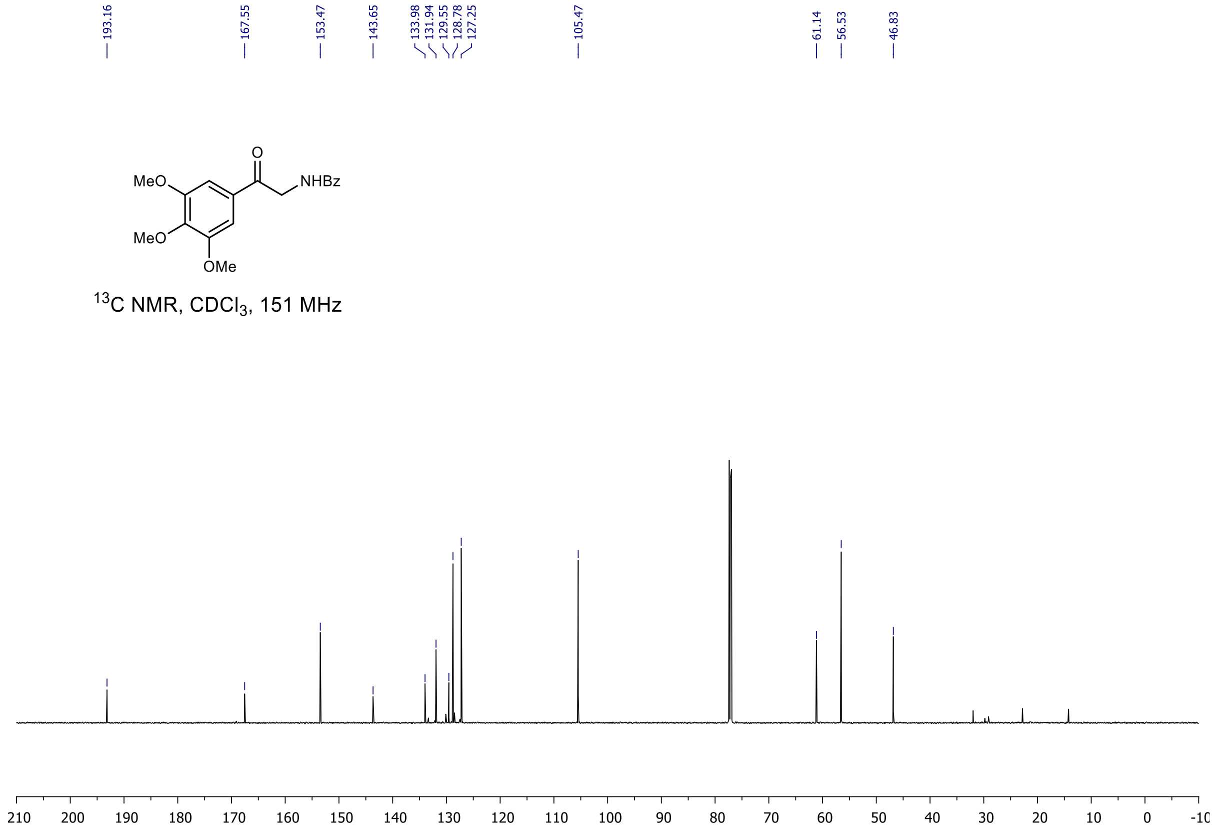

_N_-(2-(3-Fluoro-4-methoxyphenyl)-2-oxoethyl)benzamide (5e)

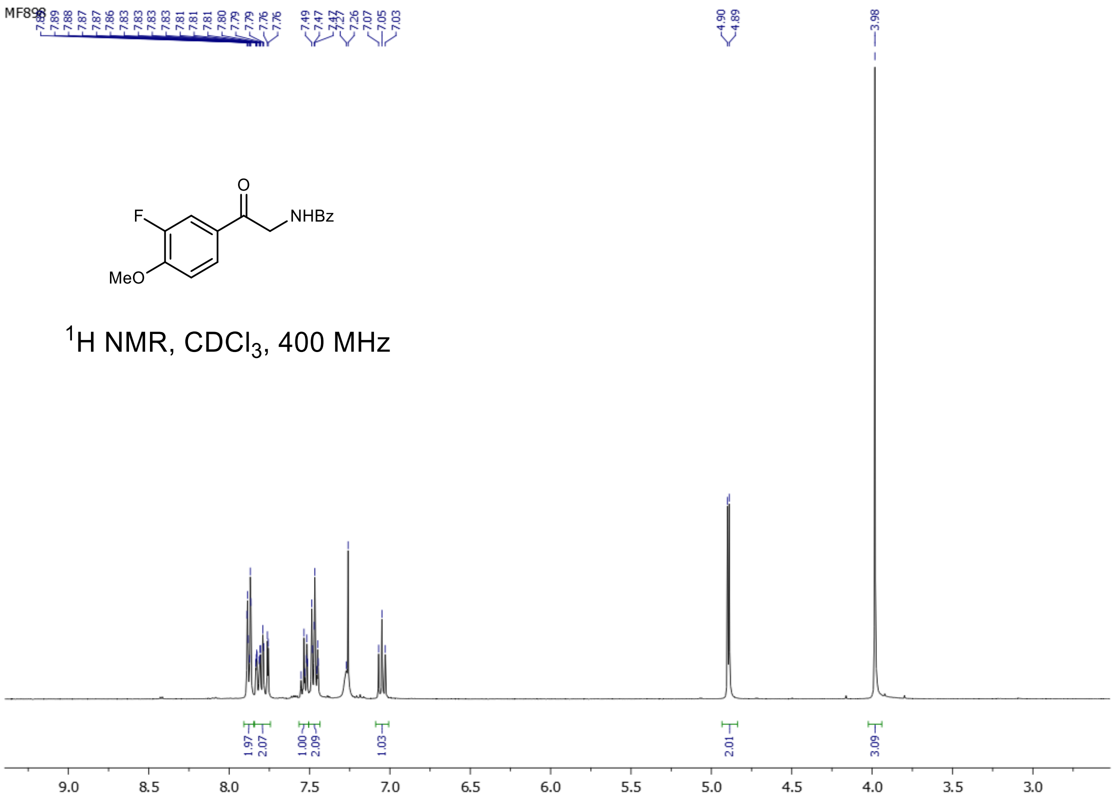

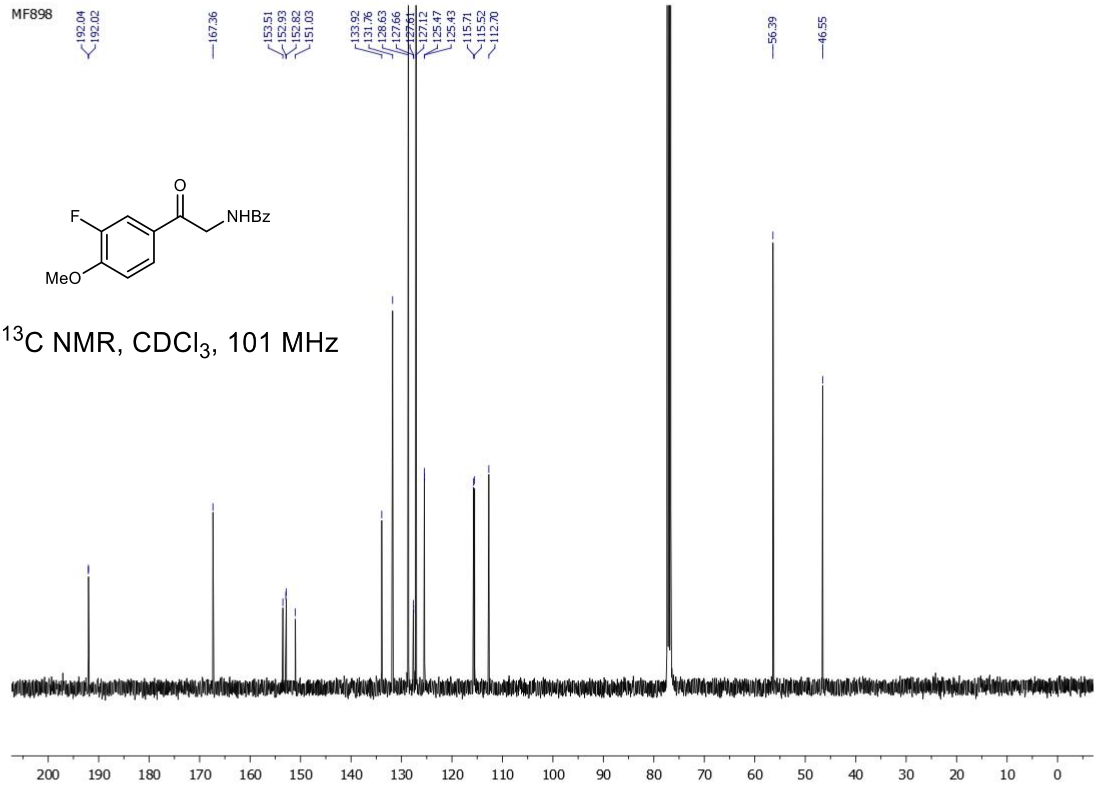

MF898

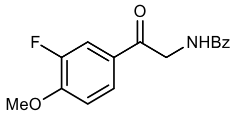

^{19}F NMR, $CDCl_3$, 377 MHz

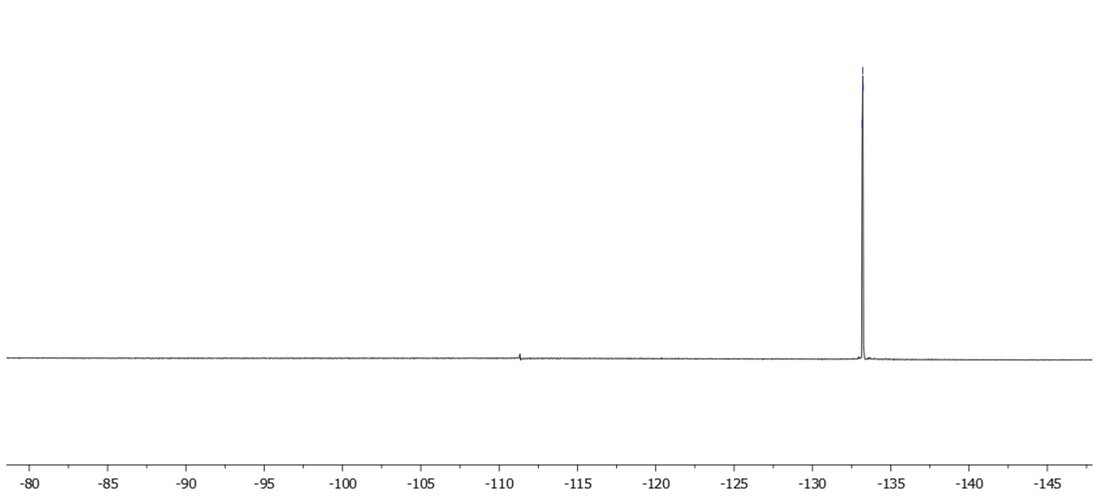

N-(2-(3-Chloro-4-methoxyphenyl)-2-oxoethyl)benzamide (5f)

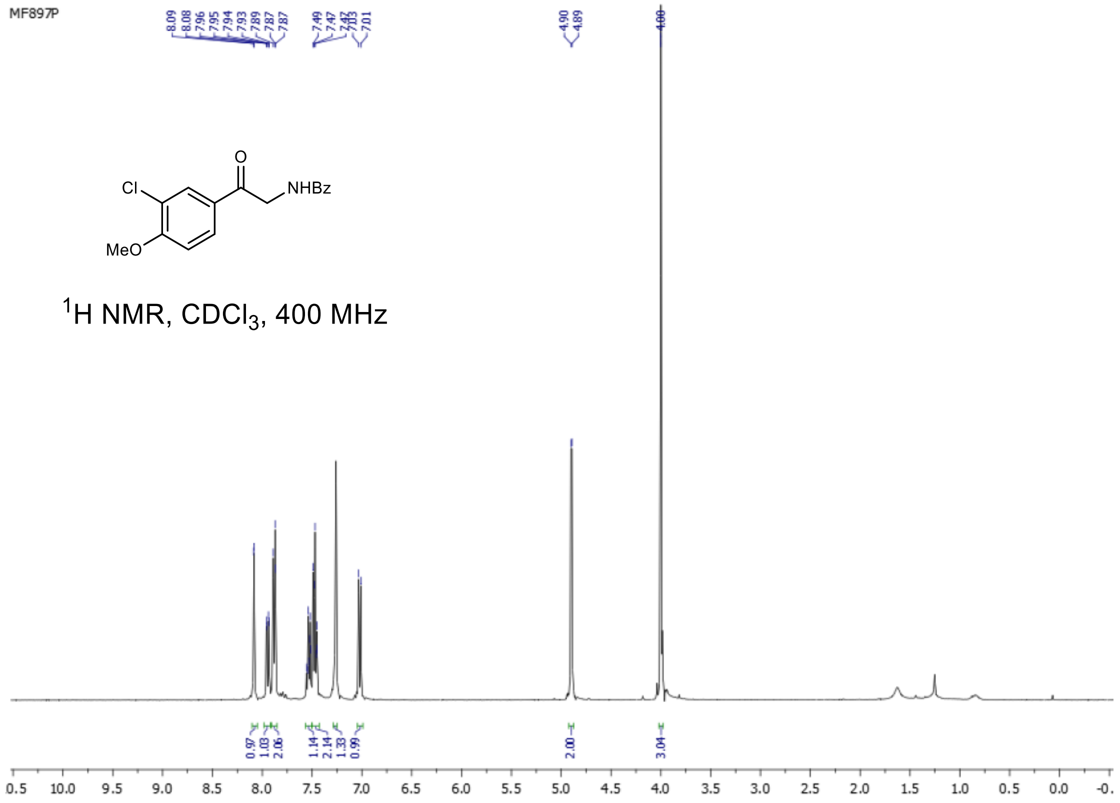

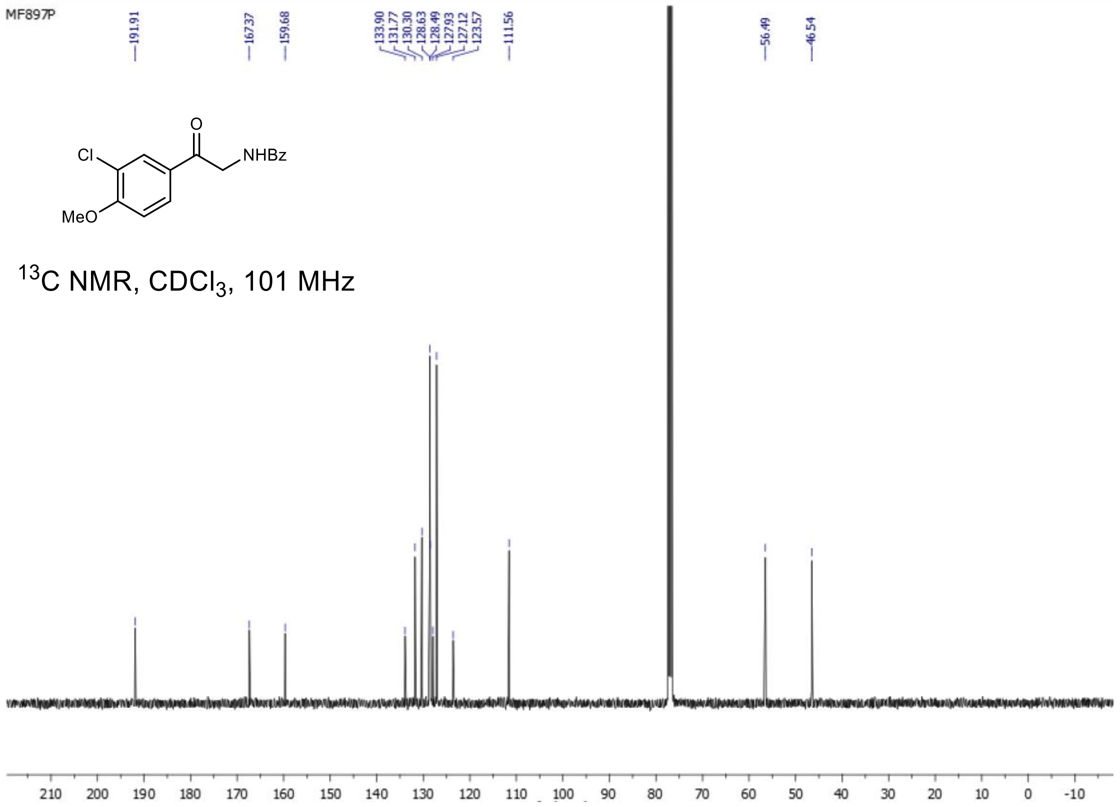

***tert*-Butyl ((2*R*)-1-((1-(dimethylamino)-1-oxo-4-phenylbutan-2-yl)amino)-1-oxo-3-phenylpropan-2-yl)carbamate (6a)**

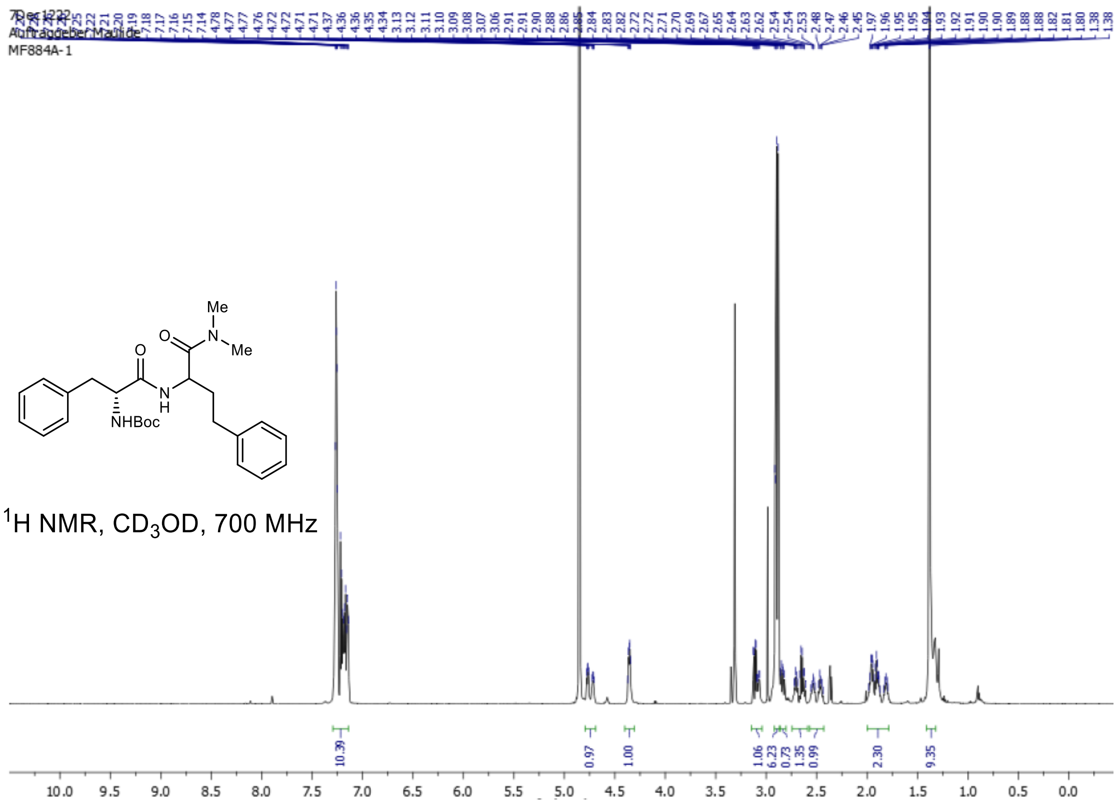

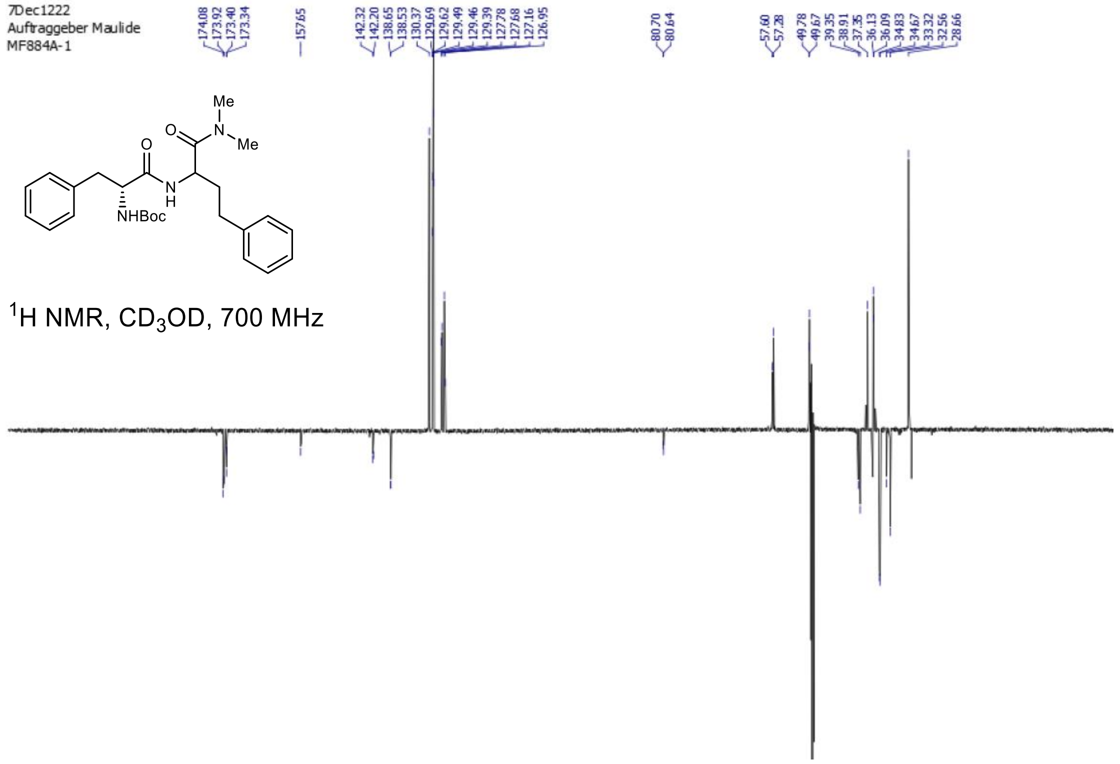

(9H-Fluoren-9-yl)methyl ((2*S*)-1-(((2*S*)-1-((1-(dimethylamino)-1-oxo-4-phenylbutan-2-yl)amino)-1-oxo-3-phenylpropan-2-yl)amino)-1-oxo-3-phenylpropan-2-yl)carbamate (6b)

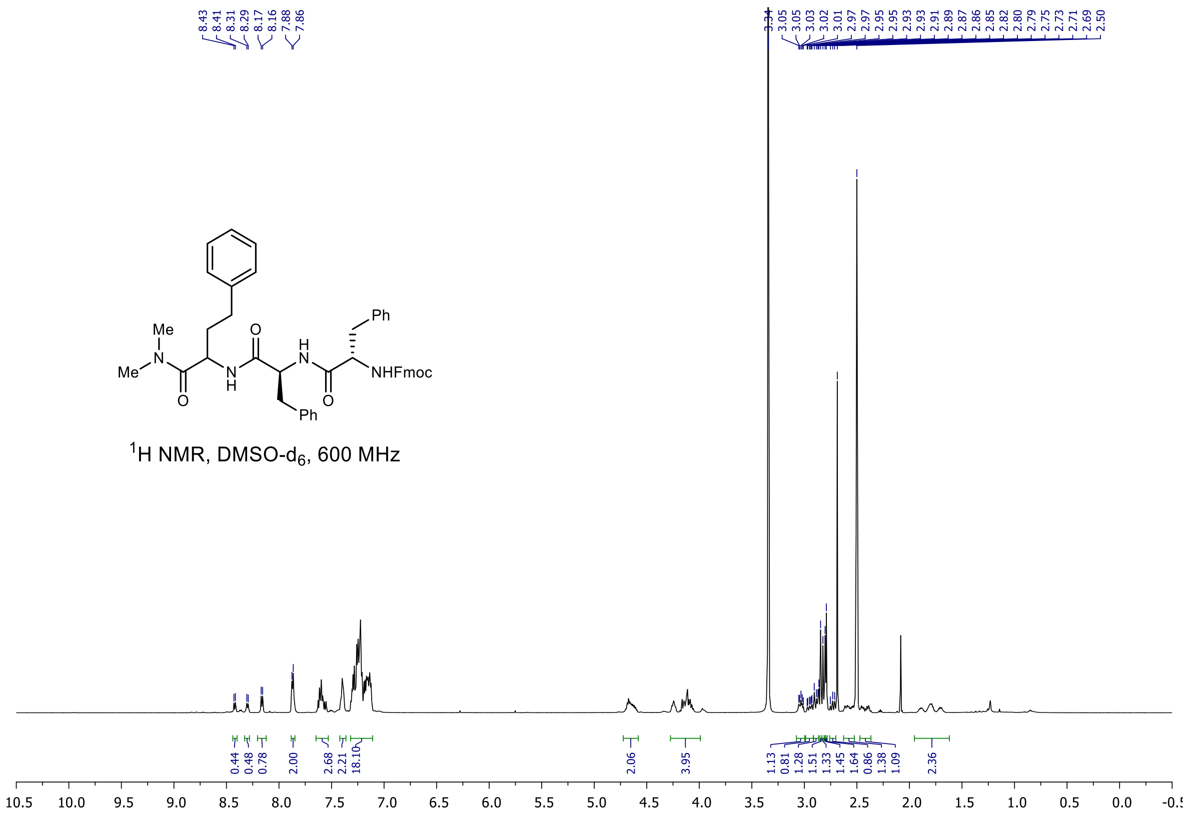

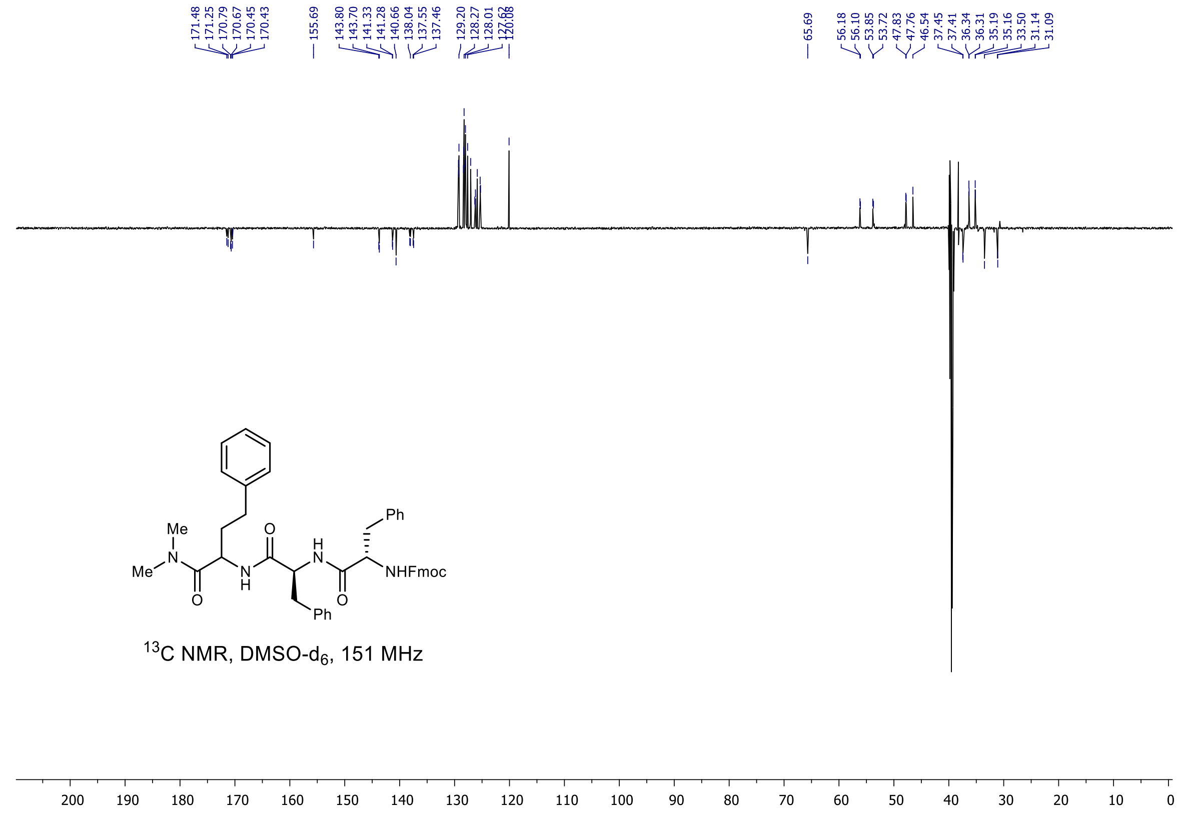

(9*H*-Fluoren-9-yl)methyl ((2*R*)-3-(*tert*-butylthio)-1-((1-cyclopropyl-2-(dimethylamino)-2-oxoethyl)amino)-1-oxopropan-2-yl)carbamate (6c)

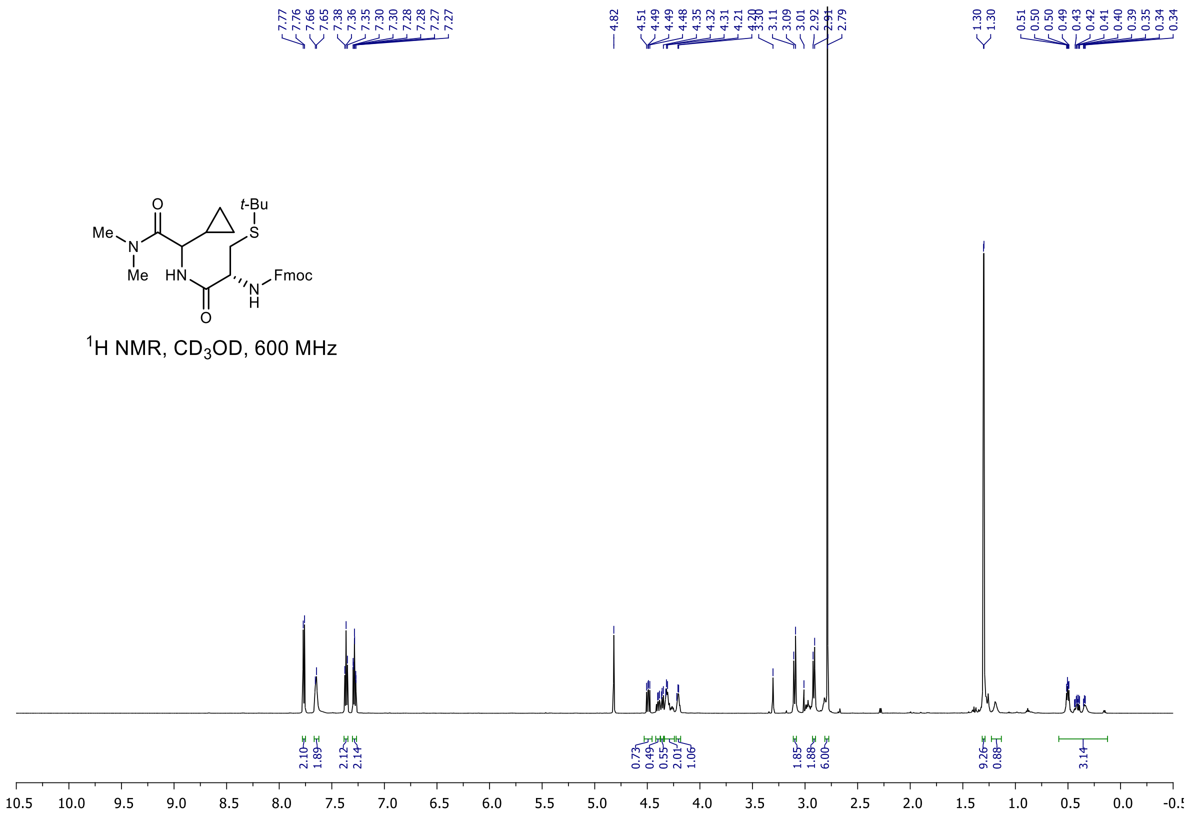

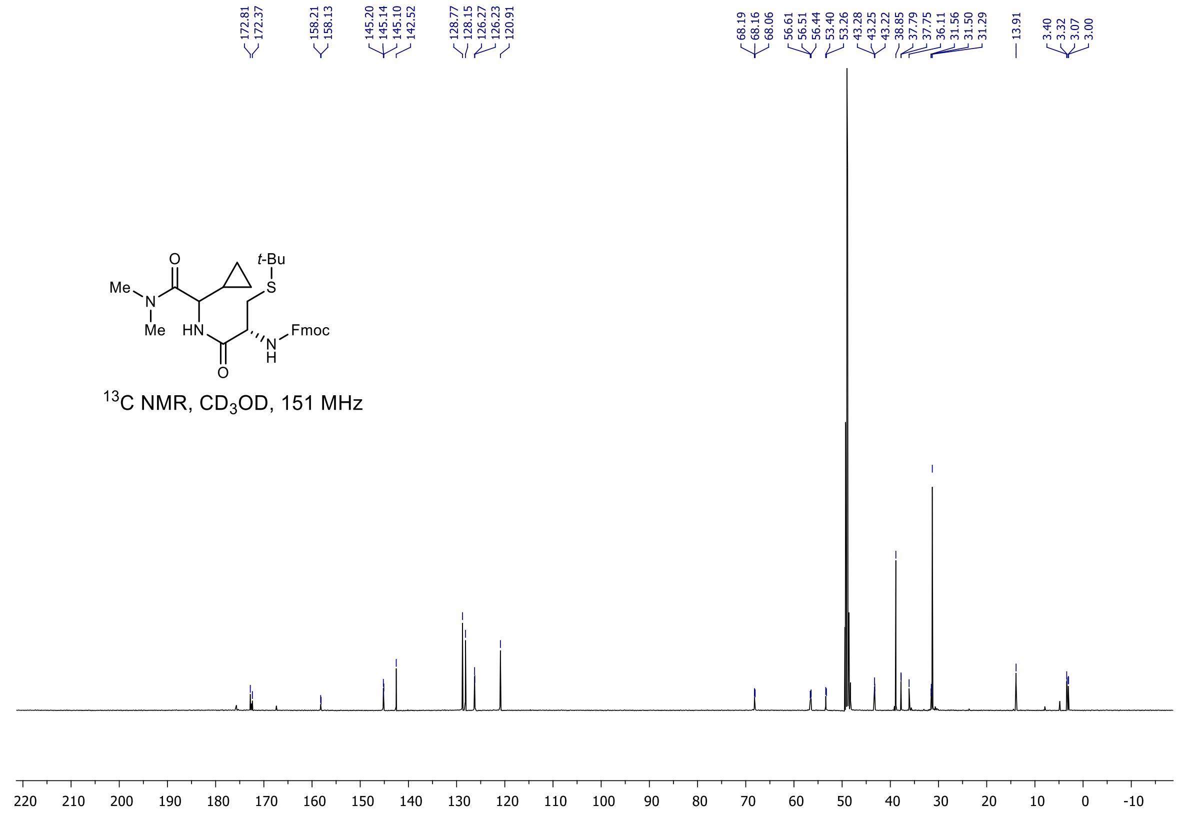

(9*H*-Fluorenyl-9-yl)methyl ((2*S*)-1-(((2*S*)-1-((2*S*)-2-((1-(dimethylamino)-1-oxo-3-(thiophen-2-yl)propan-2-yl)carbamoyl)pyrrolidin-1-yl)-1-oxopropan-2-yl)amino)-1-oxopropan-2-yl)carbamate (6d)

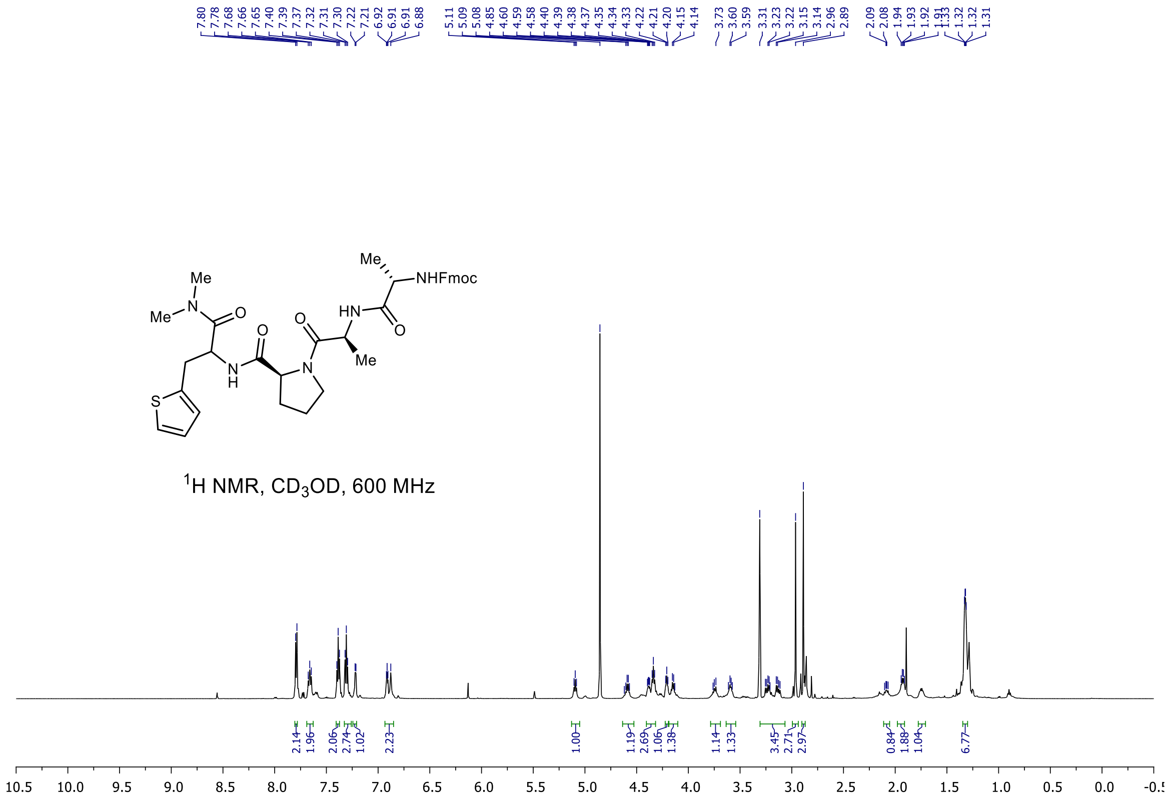

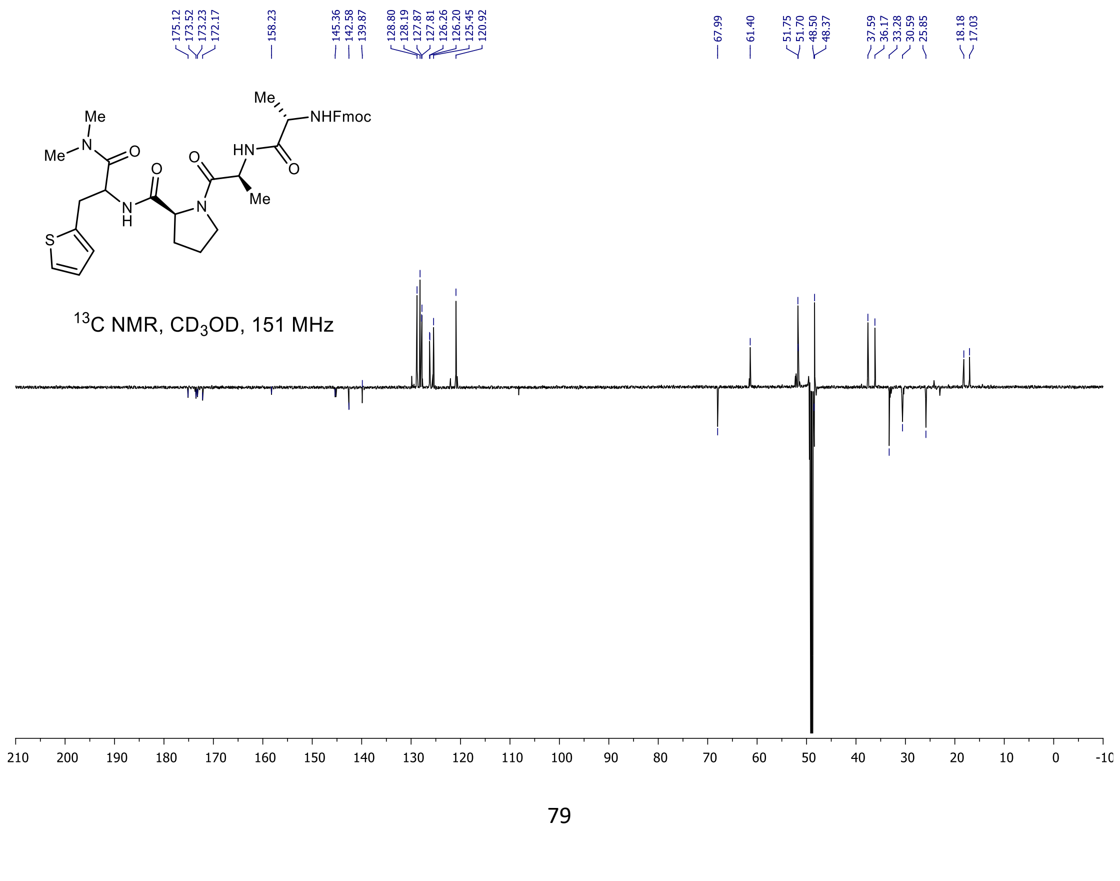

***N*,*N*-Dimethyl-1,2,3,4-tetrahydrobenzo[4,5]thieno[3,2-*c*]pyridine-3-carboxamide (7)**

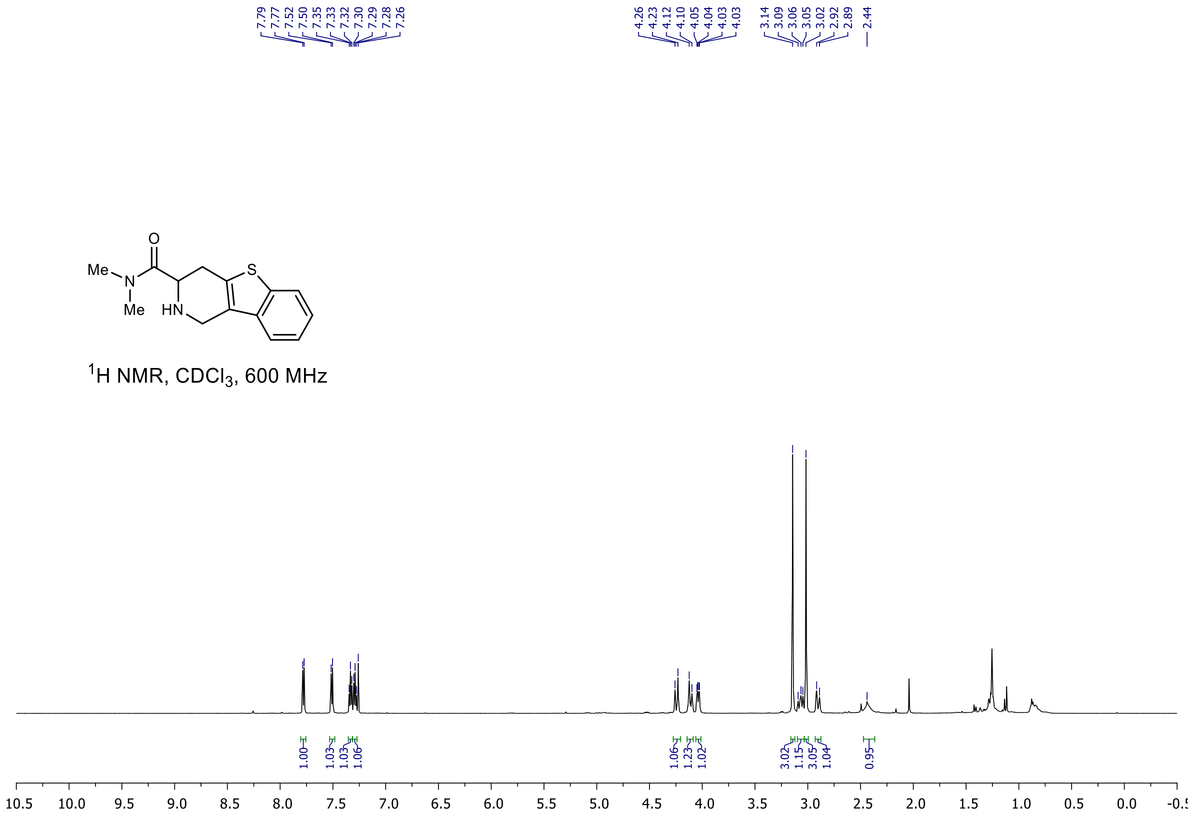

^{1}H NMR, $CDCl_3$, 600 MHz

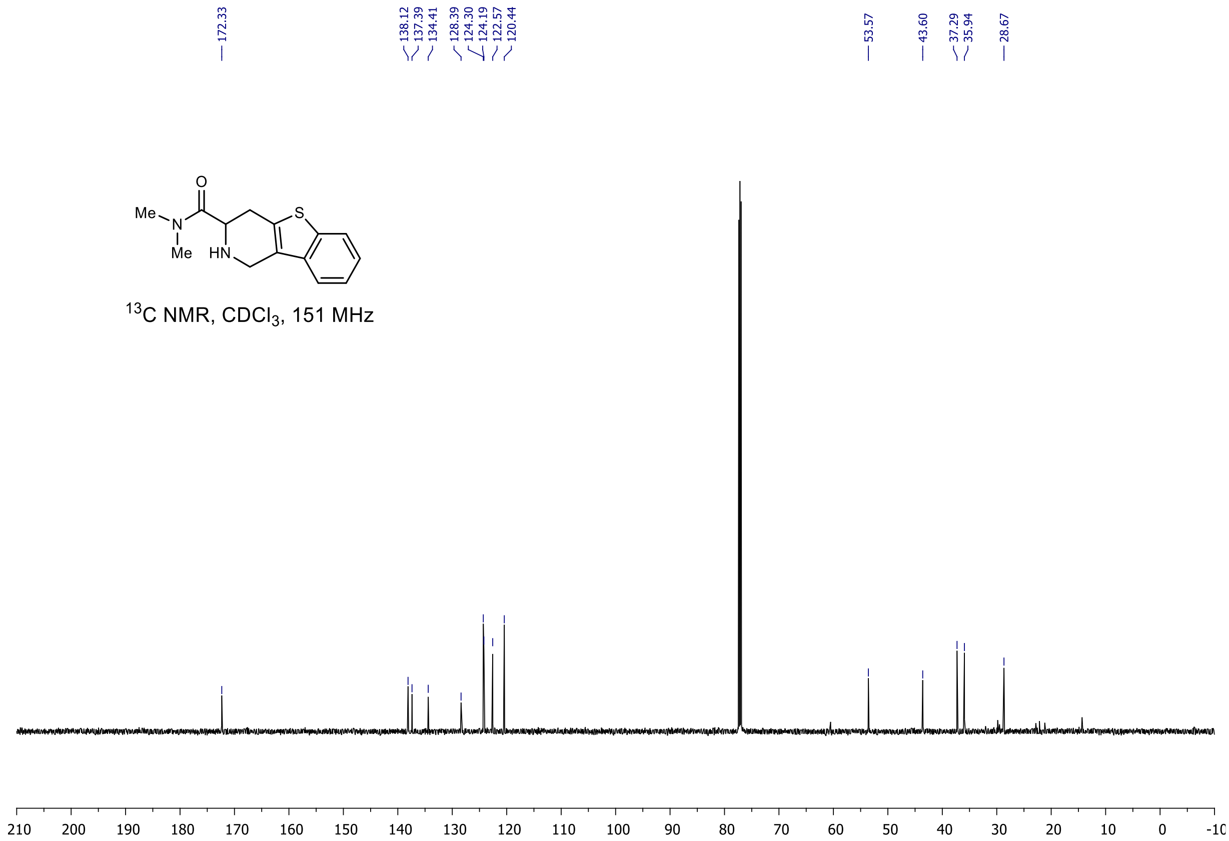

^{13}C NMR, $CDCl_3$, 151 MHz

***trans*-*N*,*N*-Dimethyl-1-phenyl-1,2,3,4-tetrahydrobenzo[4,5]thieno[3,2-*c*]pyridine-3-carboxamide (*trans*-8)**

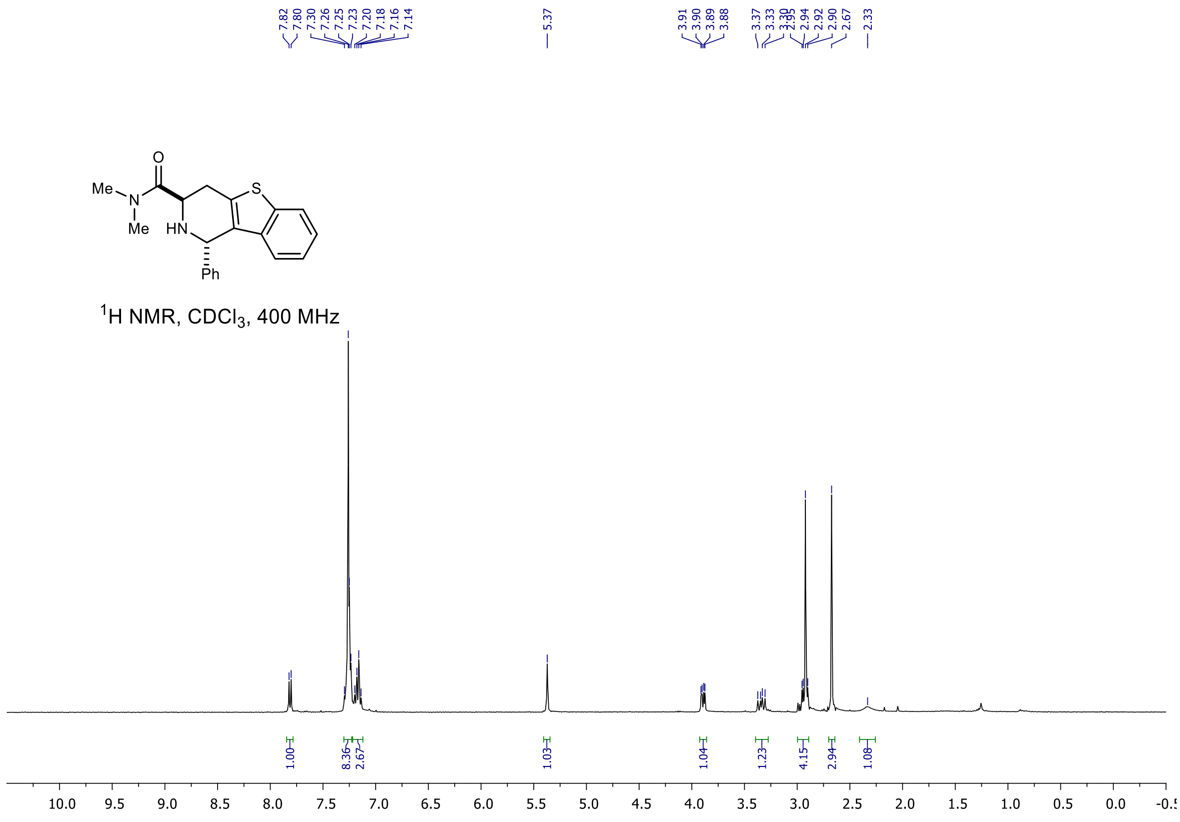

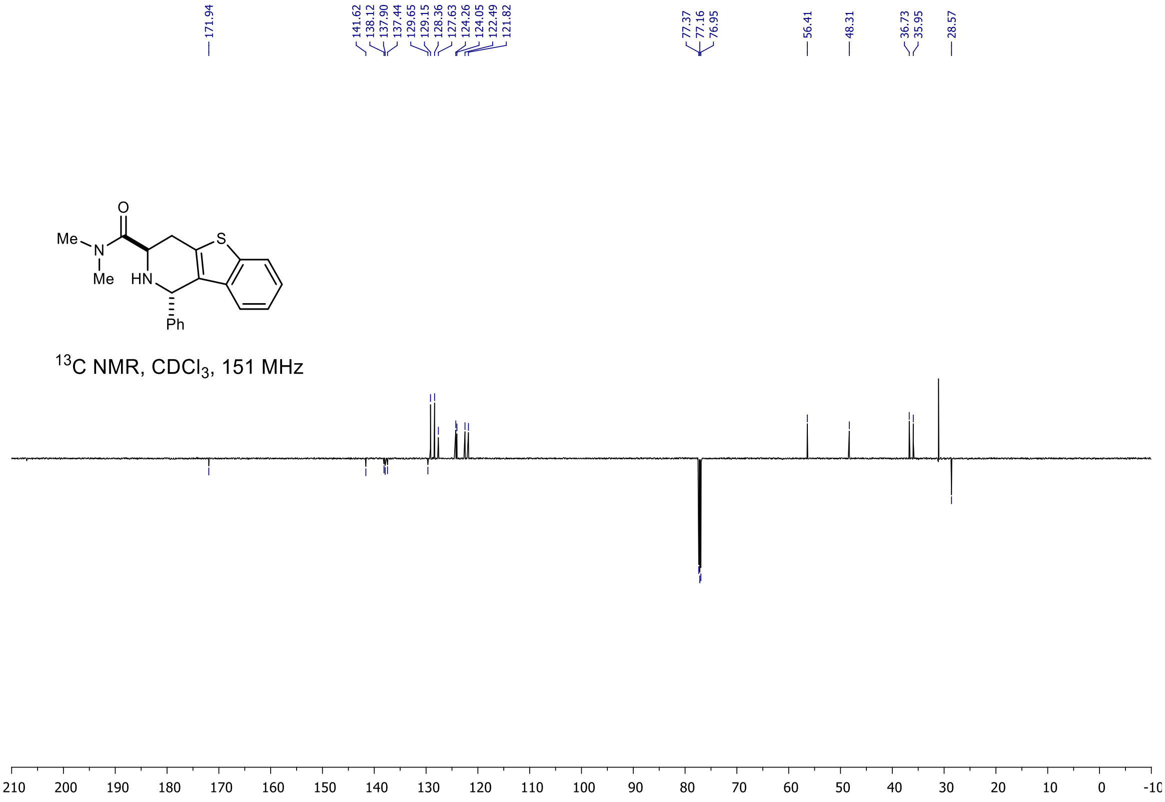

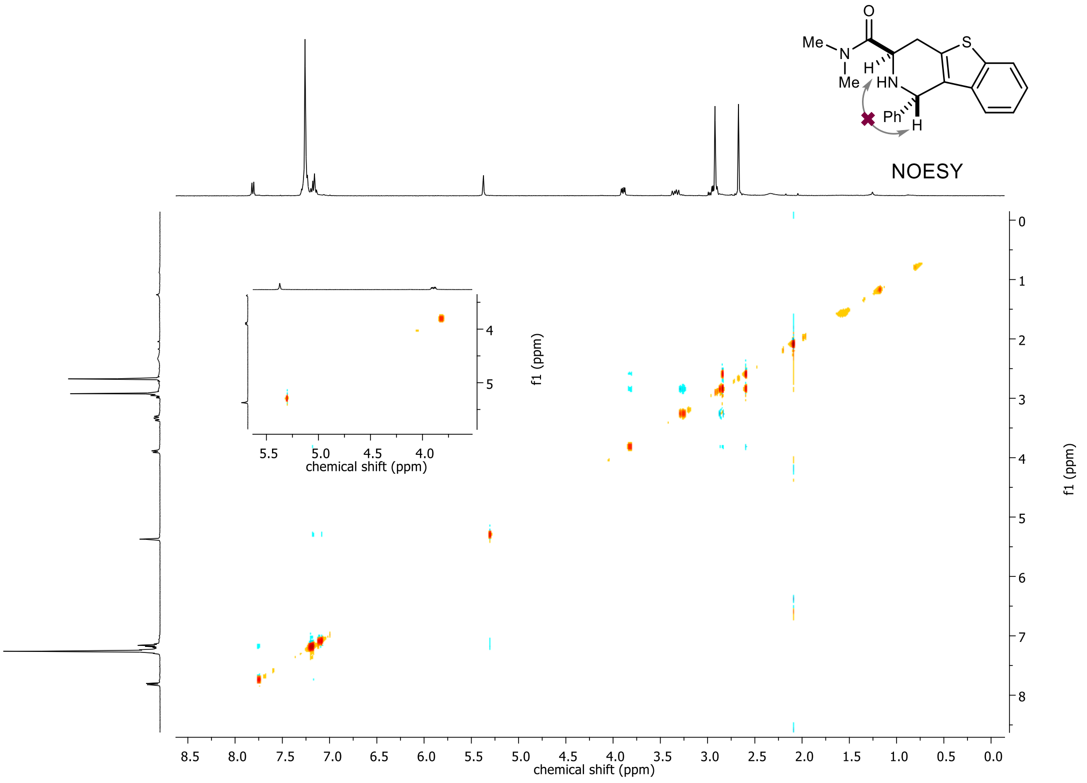
NOESY
Me
N
Me
O
H
HN
S
Ph
H
f1 (ppm)
chemical shift (ppm)

***cis*-*N*,*N*-Dimethyl-1-phenyl-1,2,3,4-tetrahydrobenzo[4,5]thieno[3,2-*c*]pyridine-3-carboxamide (*cis*-8)**

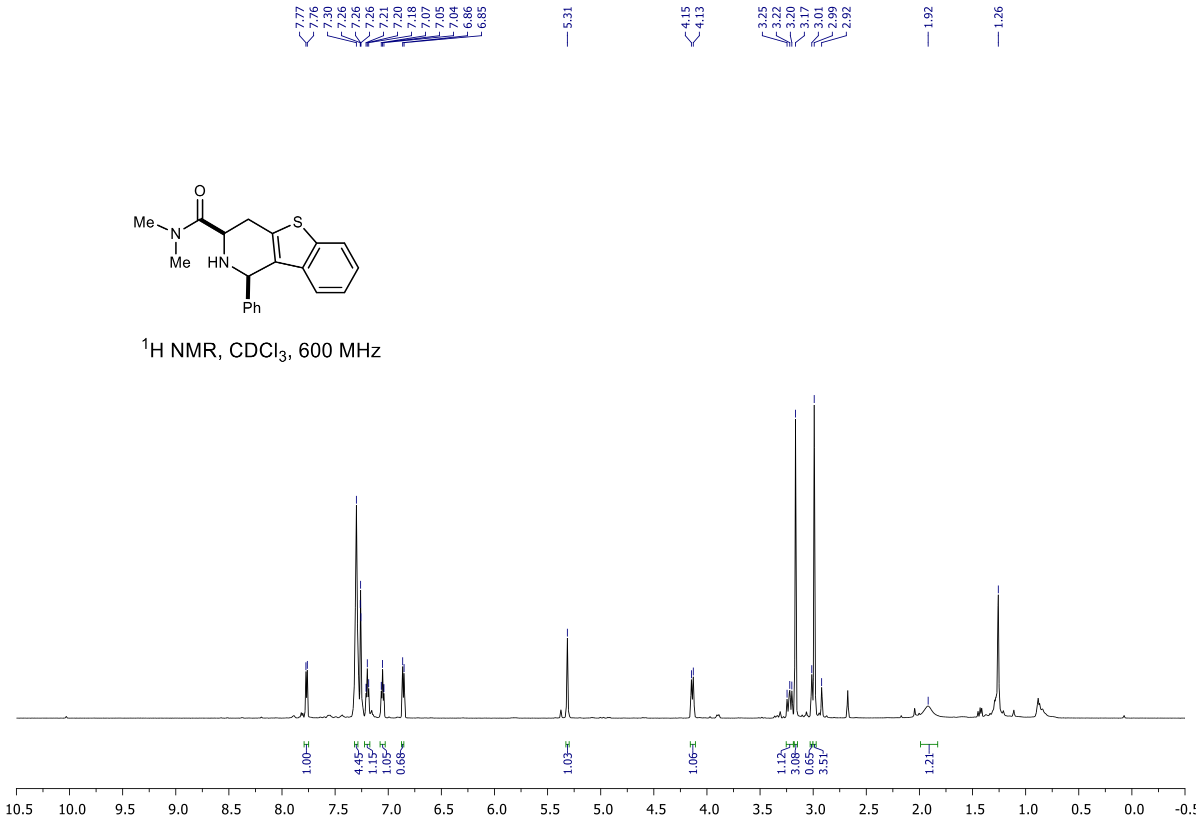

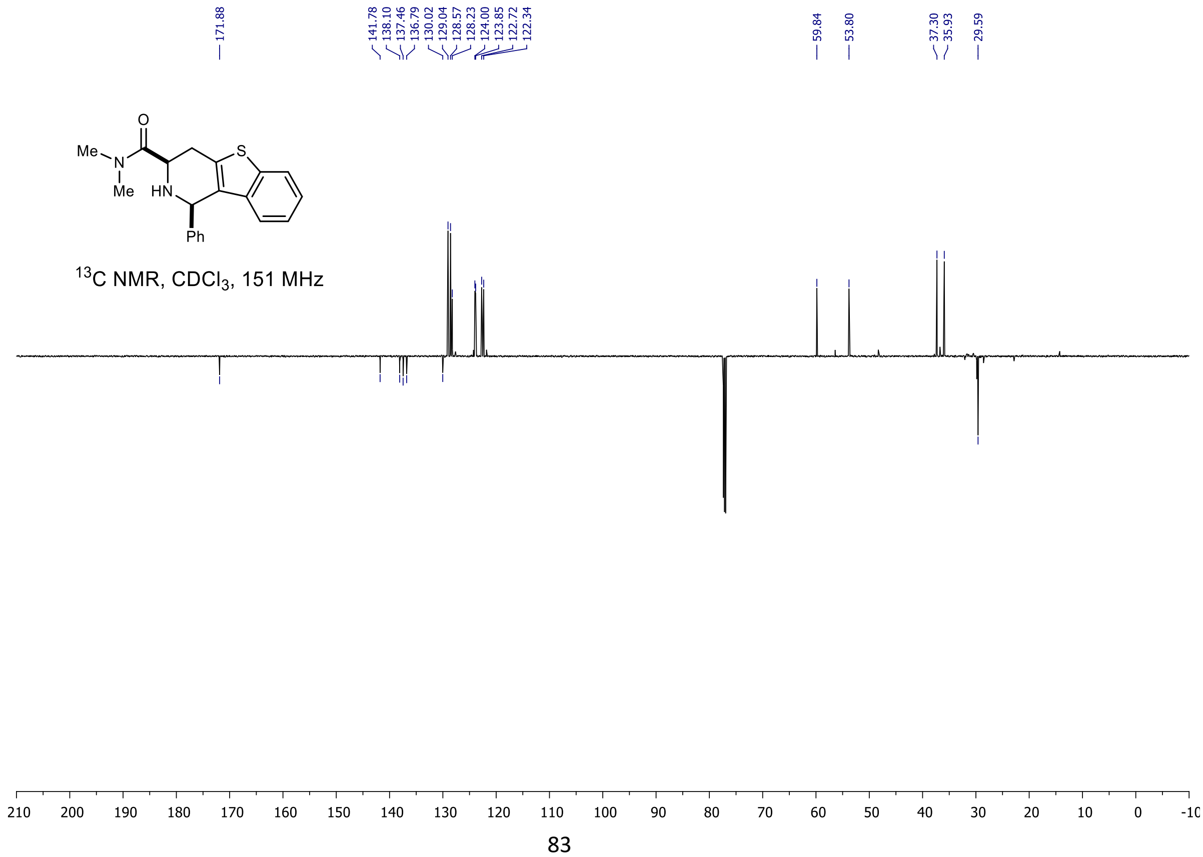

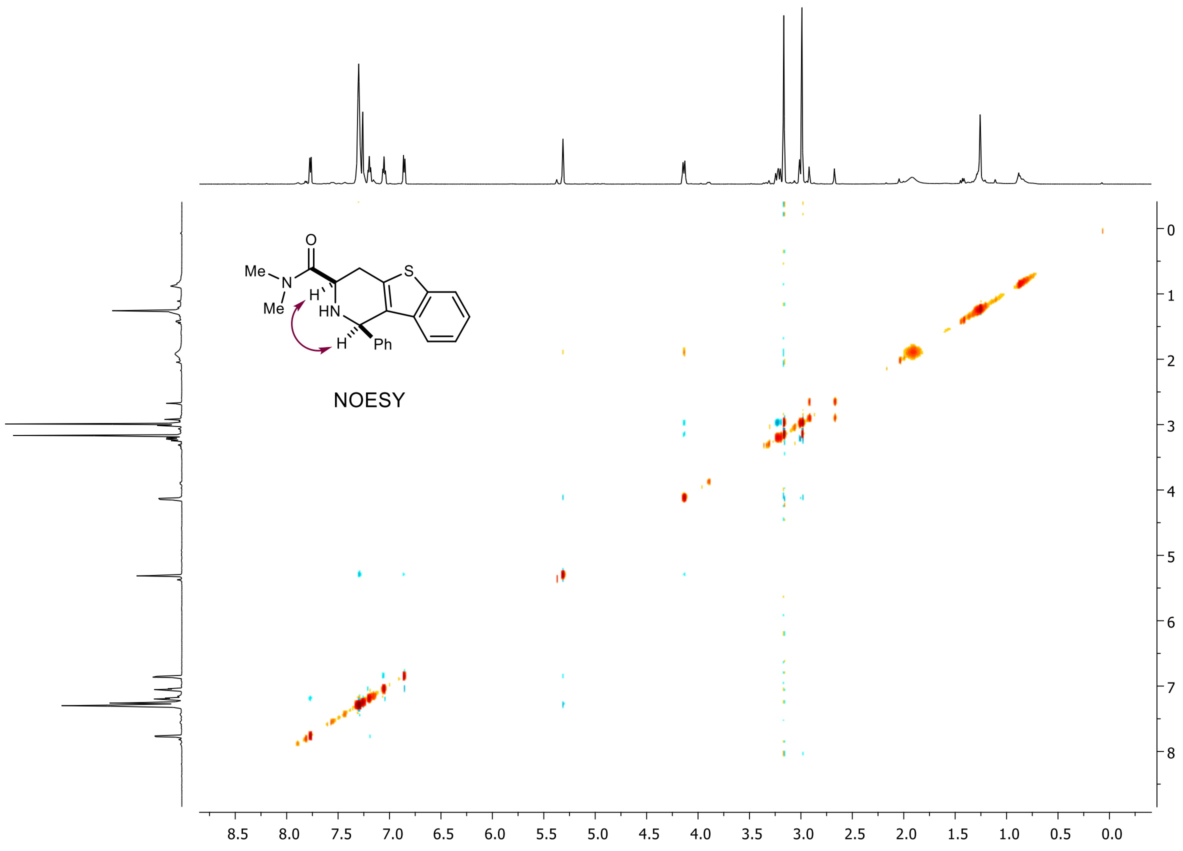

Me
N
Me
O
H
HN
H
Ph
S
NOESY
8.5
8.0
7.5
7.0
6.5
6.0
5.5
5.0
4.5
4.0
3.5
3.0
2.5
2.0
1.5
1.0
0.5
0.0
0
1
2
3
4
5
6
7
8

Indolin-1-yl(6-methylpiperidin-2-yl)methanone (9)

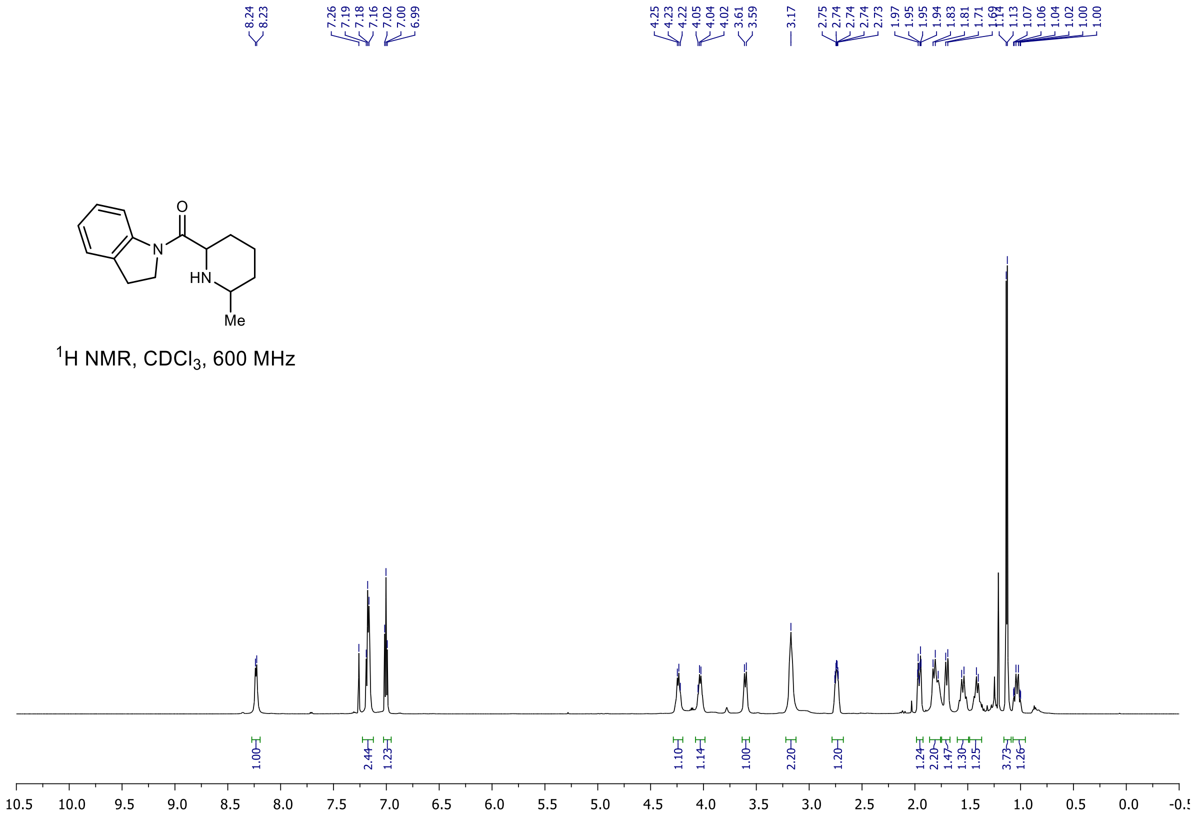

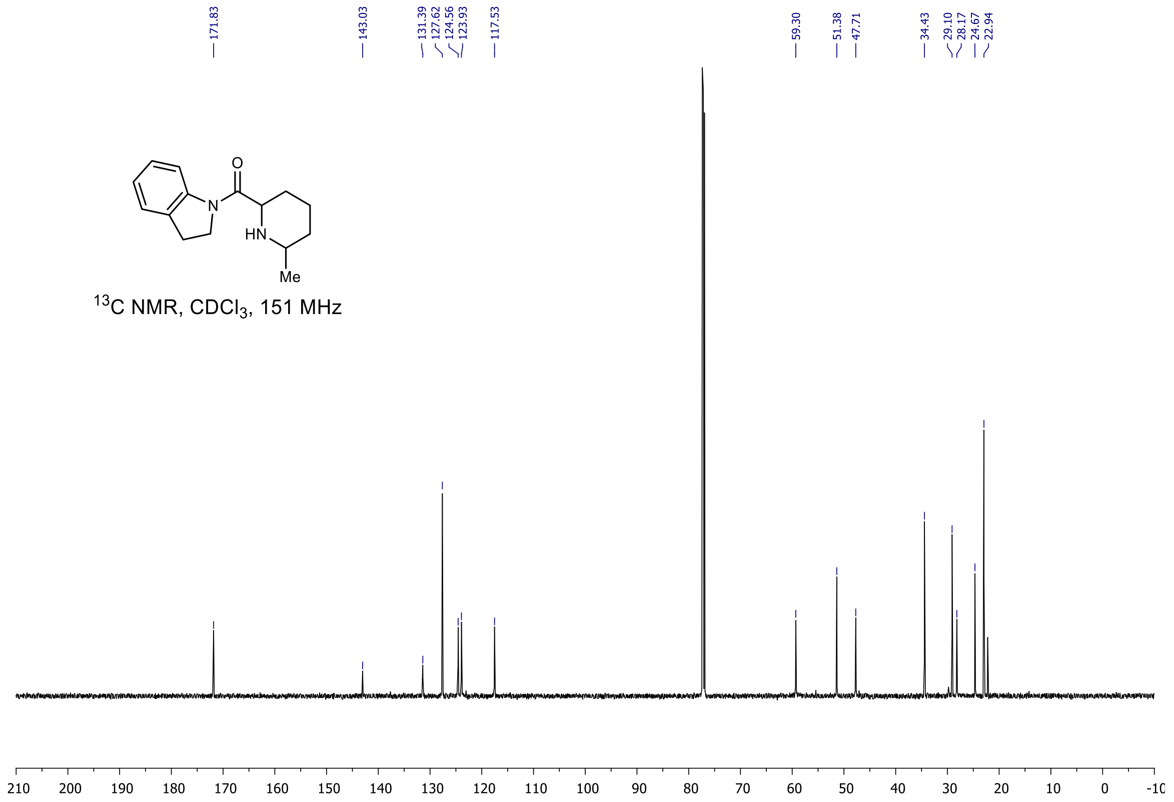

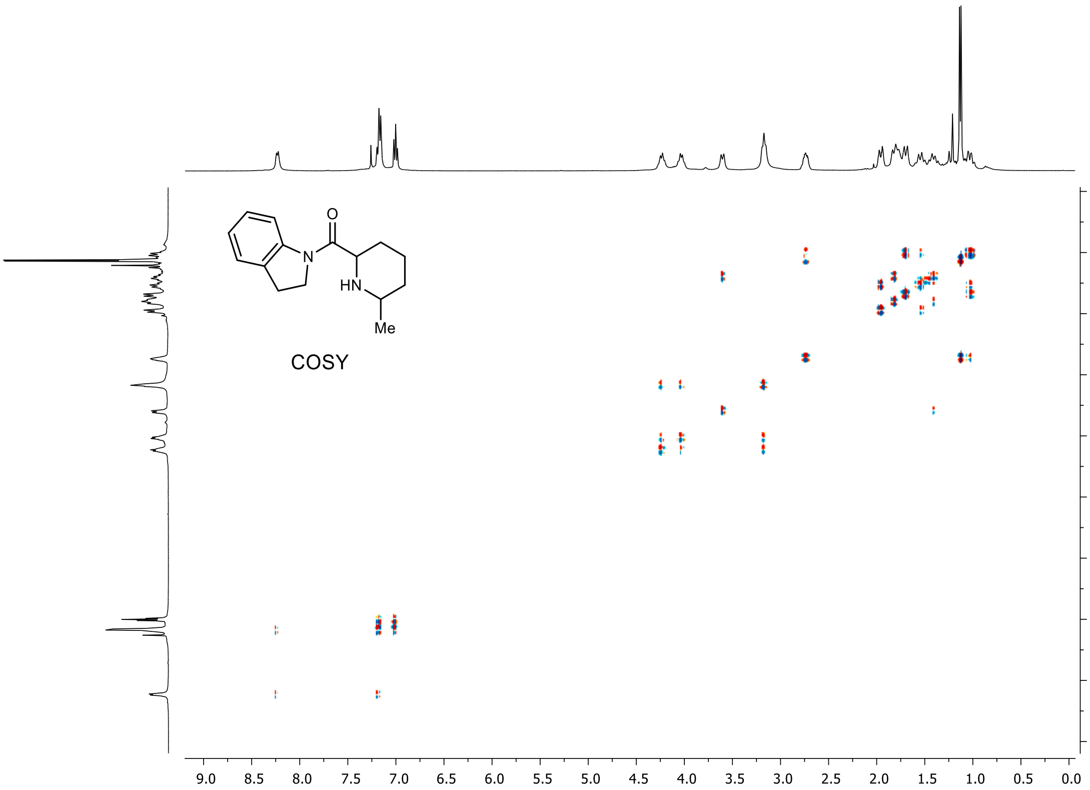
O
N
HN
Me
COSY

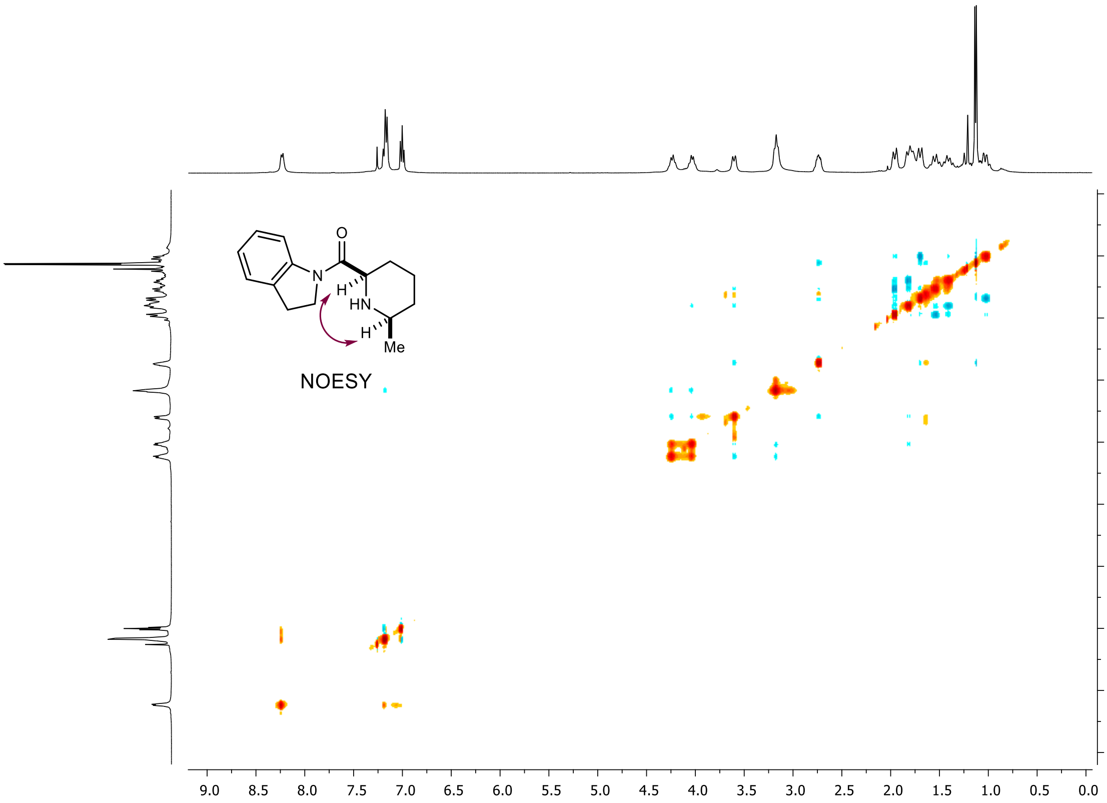
O
N
H
HN
H
Me
NOESY

***N,N*-Dimethylpiperidine-2-carboxamide (10)**

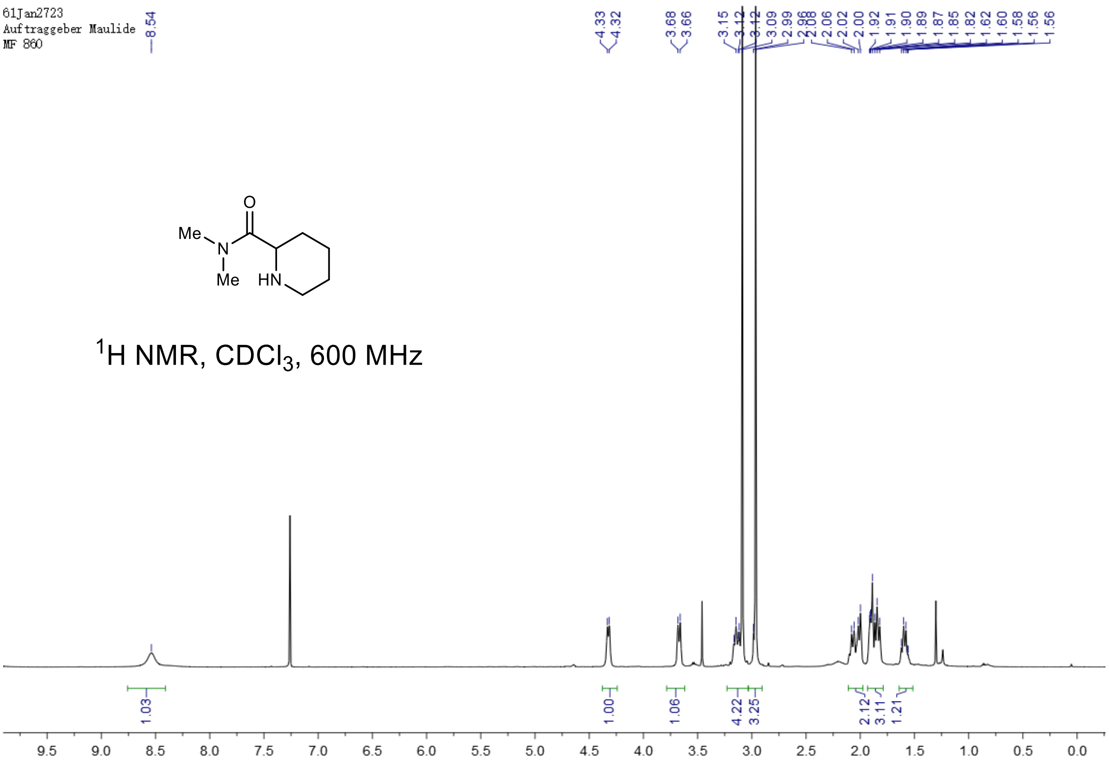

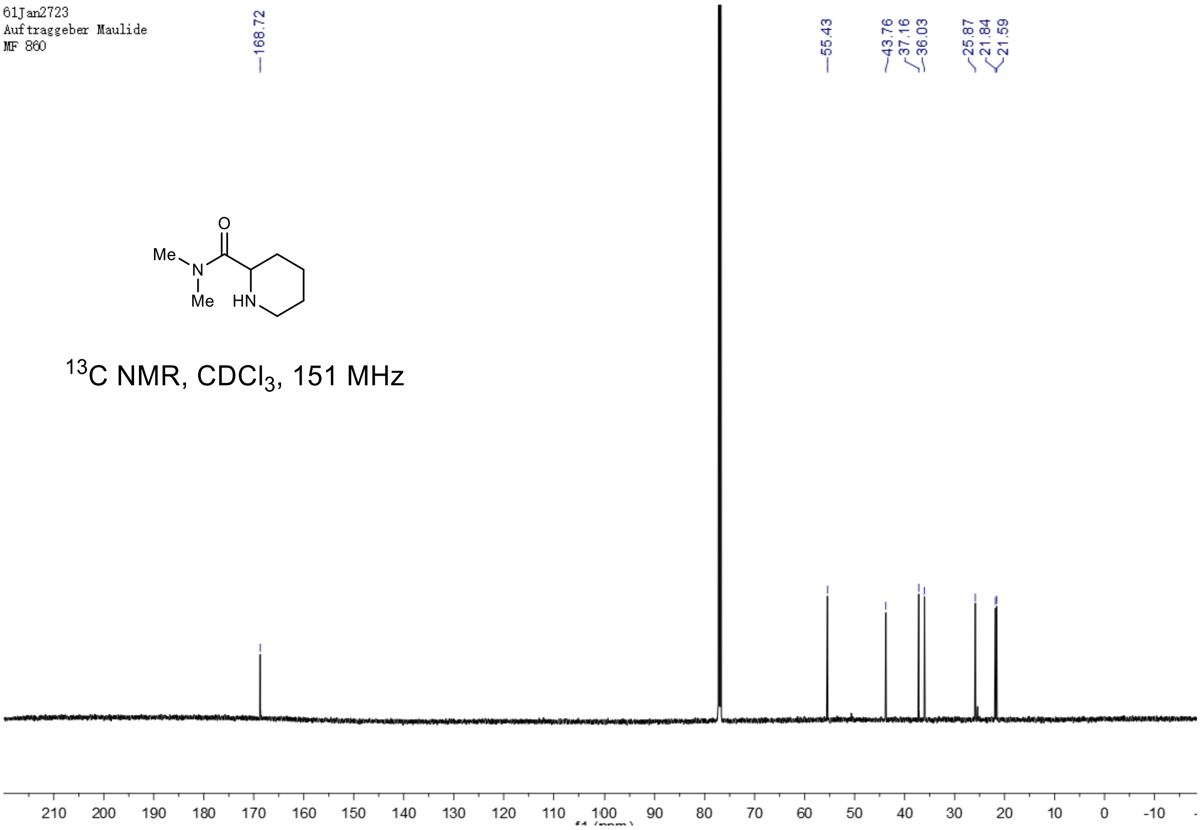

7. X-ray data

***N,N*-Dimethylpiperidine-2-carboxamide (10)**

CCDC n° 2252280

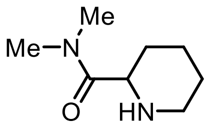

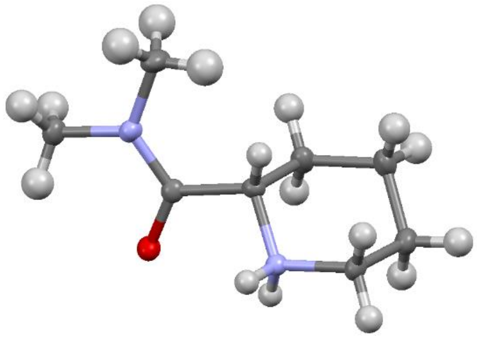

Identification code	MF860_a	
Empirical formula	$C_{30} H_{57} Cl_{21} N_6 O_3$	
Formula weight	1294.26	
Temperature	100 K	
Wavelength	0.71073 Å	
Crystal system	Triclinic	
Space group	$P2_1/n$	
Unit cell dimensions	a = 9.3970(2) Å	α= 90.0000(10)°.
	b = 30.4678(5) Å	β= 90.671(2)°.
	c = 20.1862(4) Å	γ = 90.0000(10)°.
Volume	5779.02(18) $Å^3$	
Z	4	
Density (calculated)	1.488 Mg/m^3	
Absorption coefficient	1.027 mm^{-1}	
F(000)	2640	
Crystal size	0.240 x 0.140 x 0.120 mm^3	
Theta range for data collection	1.675 to 29.593°.	
Index ranges	-13<=h<=3, -42<=k<=40, -27<=l<=28	
Reflections collected	258062	
Independent reflections	16179 [R(int) = 0.0599]	
Completeness to theta = 25.242°	100.0 %	
Absorption correction	Semi-empirical from equivalents	
Max. and min. transmission	0.5740 and 0.4539	
Refinement method	Full-matrix least-squares on F^2	
Data / restraints / parameters	16179 / 315 / 547	
Goodness-of-fit on F^2	1.051	
Final R indices [I>2sigma(I)]	R_1 = 0.0470, wR_2 = 0.1194	
R indices (all data)	R_1 = 0.0712, wR_2 = 0.1242	
Extinction coefficient	n/a	
Largest diff. peak and hole	1.974 and -1.298 e.$Å^{-3}$	

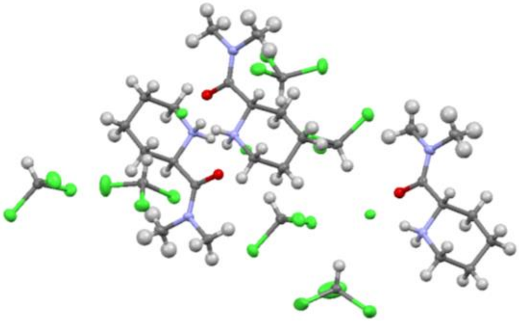

8. References

[1] P. J. Stang, T. E. Dueber, *Org. Synth.* **2003**, 79–79.

[2] H. Zhang, M. Riomet, A. Roller, N. Maulide, *Org. Lett.* **2020**, *22*, 2376–2380.

[3] V. Tona, A. de la Torre, M. Padmanaban, S. Ruider, L. González, N. Maulide, *J. Am. Chem. Soc.* **2016**, *138*, 8348–8351.

[4] S. Das, D. Addis, S. Zhou, K. Junge, M. Beller, *J. Am. Chem. Soc.* **2010**, *132*, 1770–1771.

[5] M. Shi, N. Ye, W. Chen, H. Wang, C. Cheung, M. Parmentier, F. Gallou, B. Wu, *Org. Process Res. Dev.* **2020**, *24*, 1543–1548.

[6] S. B. Lang, R. J. Wiles, C. B. Kelly, G. A. Molander, *Angew. Chem. Int. Ed.* **2017**, *56*, 15073–15077.

[7] J. Xue, Y.-S. Zhang, Z. Huan, J.-D. Yang, J.-P. Cheng, *J. Org. Chem.* **2022**, *87*, 15539–15546.

[8] S. Tortoioli, L. Bannwart, S. Abele, *Synthesis* **2016**, *48*, 2069–2078.

[9] C. Madelaine, V. Valerio, N. Maulide, *Angew. Chem. Int. Ed.* **2010**, *49*, 1583–1586.

[10] C. J. Teskey, P. Adler, C. R. Gonçalves, N. Maulide, *Angew. Chem. Int. Ed.* **2019**, *58*, 447–451.

[11] Y. K. Jang, T. Krückel, M. Rueping, O. El-Sepelgy, *Org. Lett.* **2018**, *20*, 7779–7783.

[12] T. J. Montavon, J. Li, J. R. Cabrera-Pardo, M. Mrksich, S. A. Kozmin, *Nat. Chem.* **2011**, *4*, 45–51.

[13] V. Porte, G. Di Mauro, M. Schupp, D. Kaiser, N. Maulide, *Chem. Eur. J.* **2020**, *26*, 15509–15512.

[14] Y.-L. Zhang, R.-T. Guo, J.-H. He, X.-C. Wang, *Org. Lett.* **2019**, *21*, 4239–4244.

[15] A. Citarella, D. Gentile, A. Rescifina, A. Piperno, B. Mognetti, G. Gribaudo, M. T. Sciortino, W. Holzer, V. Pace, N. Micale, *Int. J. Mol. Sci.* **2021**, *22*, 1398.
